# Intron retention and transcript chimerism conserved across mammals: Ly6g5b and Csnk2b-Ly6g5b as examples

**DOI:** 10.1186/1471-2164-14-199

**Published:** 2013-03-22

**Authors:** Francisco Hernández-Torres, Alberto Rastrojo, Begoña Aguado

**Affiliations:** 1 Centro de Biología Molecular Severo Ochoa (CBMSO), Consejo Superior de Investigaciones Científicas (CSIC)-Universidad Autónoma de Madrid, Madrid, Spain; 2 Present address: Experimental Biology Department, Universidad de Jaén, Jaén, Spain

## Abstract

**Background:**

Alternative splicing (AS) is a major mechanism for modulating gene expression of an organism, allowing the synthesis of several structurally and functionally distinct mRNAs and protein isoforms from a unique gene. Related to AS is the Transcription Induced Chimerism (TIC) or Tandem Chimerism, by which chimeric RNAs between adjacent genes can be found, increasing combinatorial complexity of the proteome. The *Ly6g5b* gene presents particular behaviours in its expression, involving an intron retention event and being capable to form RNA chimera transcripts with the upstream gene *Csnk2b*. We wanted to characterise these events more deeply in four tissues in six different mammals and analyse their protein products.

**Results:**

While canonical *Csnk2b* isoform was widely expressed, *Ly6g5b* canonical isoform was less ubiquitous, although the *Ly6g5b* first intron retained transcript was present in all the tissues and species analysed. *Csnk2b-Ly6g5b* chimeras were present in all the samples analysed, but with restricted expression patterns. Some of these chimeric transcripts maintained correct structural domains from *Csnk2b* and *Ly6g5b*. Moreover, we found *Csnk2b, Ly6g5b,* and *Csnk2b-Ly6g5b* transcripts that present exon skipping, alternative 5' and 3' splice site and intron retention events. These would generate truncated or aberrant proteins whose role remains unknown. Some chimeric transcripts would encode *CSNK2B* proteins with an altered C-terminus, which could affect its biological function broadening its substrate specificity. Over-expression of human *CSNK2B, LY6G5B,* and *CSNK2B-LY6G5B* proteins, show different patterns of post-translational modifications and cell distribution.

**Conclusions:**

*Ly6g5b* intron retention and *Csnk2b-Ly6g5b* transcript chimerism are broadly distributed in tissues of different mammals.

## Background

To date, a high number of eukaryotic genomes have been sequenced. Surprisingly, it is interesting to observe that *Homo sapiens* and *Caenorhabditis elegans* genomes contain a similar number of protein-coding genes (~21.000), according to the Ensembl (http://www.ensembl.org/) database. Initially, these findings disconcerted researchers who thought the number of genes should be correlated with developmental and physiological complexity, and made them realize that other mechanisms should be involved in this evolutionary variety. Alternative splicing (AS) is a major mechanism for modulating gene expression of an organism, and enables a single gene to increase its expression capacity, allowing the synthesis of several structurally and functionally distinct mRNAs and protein isoforms from a unique gene (For reviews see [1-3]). This mechanism, which was initially described in viruses [4-6], is now known to affect 95% of all human genes [7] and has been proposed as a primary driver of the evolution of phenotypic complexity in mammals [8-10]. The human Major Histocompatibility Complex (MHC) is located on chromosome 6, and is ~4 Mb in length. It is composed by three regions, the class I and class II regions flanking the central class III region. The class III region is ~0.9 Mb in length and contains 62–64 genes and 2–3 pseudogenes, depending on the haplotype [11,12]. Previously, our group precisely defined the AS patterns of a five gene cluster from the Lymphocyte antigen-6 (LY-6) superfamily [13] and characterised the expression of the corresponding proteins [14] in human and mouse. LY-6 superfamily members are cysteine-rich, generally GPI-anchored, cell surface proteins, which have definite or putative immune-related roles [15]. Among these LY-6 MHC class III region genes, *Ly6g5b* showed a particular behaviour in the regulation of its expression [13,16], involving an intron retention event in human and mouse, the rarest form of alternative splicing found in metazoan species [17]. The intron retained is the first one after the initial exon and interrupts the open reading frame (ORF) just after the signal peptide introducing a premature stop codon (PSC). The presence of a PSC at this position should cause this intron-retained transcript to undergo Nonsense Mediated Decay (NMD) [18,19]. However, this transcript seemed to escape NMD and was more abundant than the correctly spliced mRNA [13,16]. In addition, findings in our laboratory showed the presence of *LY6G5B* gene exons in transcripts derived from the upstream gene *CSNK2B*[16], which encodes the *Casein Kinase II β subunit*, a ubiquitous protein kinase which regulates metabolic pathways, signal transduction, transcription, translation, and replication [20,21]. The Transcription Induced Chimerism (TIC) or Tandem Chimerism, is a phenomenon whose mechanism still remains largely unknown, although it is being promoted as a novel way to increase combinatorial complexity of the proteome [22,23]. At least 4%-5% of the tandem gene pairs in the human genome can be eventually transcribed into a single RNA sequence encoding a putative chimeric protein [22] but recent bioinformatic analyses, partially supported by experimental data, show that this phenomenon could be quite frequent [24,25]. Recently, it has been showed that these chimeras significantly exploit signal peptides and transmembrane domains, which could alter the cellular localisation of cognate proteins, and that chimeric RNAs are more tissue specific than non-chimeric transcripts [26]. Thus, this novel mechanism directly related to AS could have an important role in evolution divergence. In this regard, the majority of these studies give relevance to this phenomenon in *Homo sapiens*[24], but detailed comparative analyses among different species are, to our knowledge, not described. Here, we have deeply characterised the expression of *Ly6g5b* and *Csnk2b* transcripts independently and of the *Csnk2b-Ly6g5b* chimeric transcripts in four defined tissues among six different mammals. We conclude that *Ly6g5b* intron retention and *Csnk2b-Ly6g5b* chimerism is present in the tissues of the analysed mammalian group. In addition, we have made a comparative analysis of human CSNK2B*,* LY6G5B*,* and CSNK2B*-*LY6G5B protein expressions.

## Results

### *Csnk2b* transcript analysis

**Figure 1:**
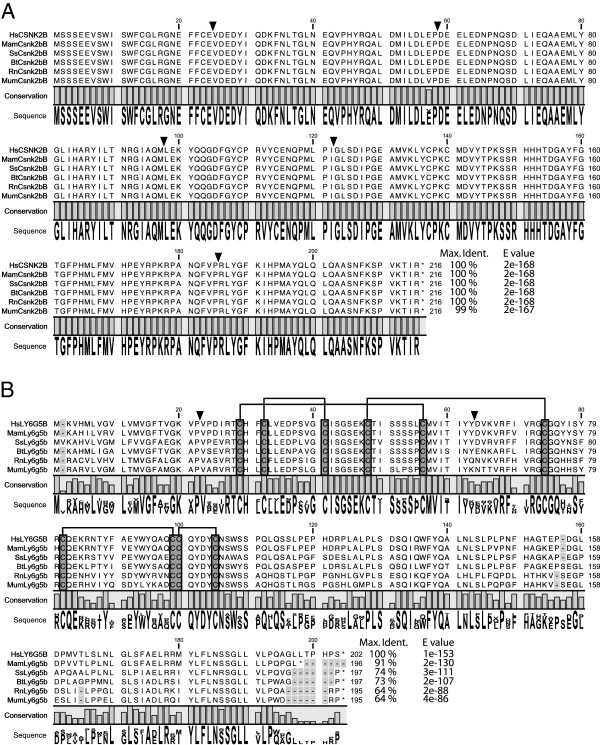
***Homo sapiens *(Hs)*, Macaca mulata *(Mam)*, Sus scrofa *(Ss)*, Bos taurus *(Bt)*, Rattus norvegicus *(Rn) and *Mus musculus *(Mum) ORF alignments. A**) Csnk2b: N-terminal region α1-α5 (5-104), juxta-dimer interface region including zinc-finger (105-147) and α6 (163-175), C-terminal region (178-205) including interaction with CSNK2α (175-193) and dimerization (190-205) regions [27], **B**) Ly6g5b: The conserved cysteines, characteristic for Ly-6 domain, are shown highlighted in grey and their connectivity with interconnected lines. Exon-exon junctions are indicated by inverted triangles. The canonical (or theoretical canonical) sequence for each specie is only shown. The percentage of identity of each sequence respect to the human one is shown as well as the corresponding E values.

**Figure 2:**
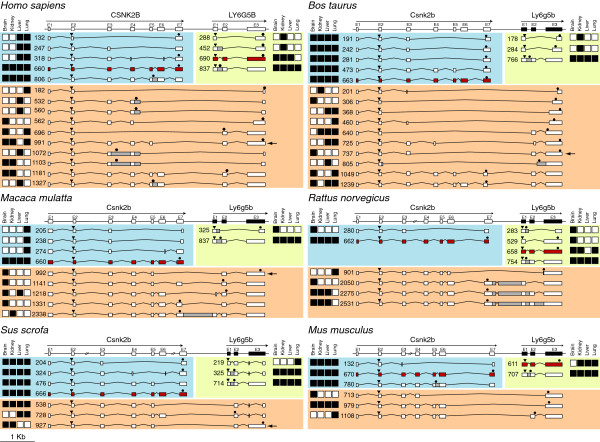
**Schematic representation of all Csnk2b and Ly6g5b transcripts detected through nested RT-PCR on each analysed tissue for *Homo sapiens, Macaca mulata, Sus scrofa, Bos Taurus, Rattus norvegicus and Mus musculus.*** White boxes represent Csnk2b, Ly6g5b and chimera Csnk2b-Ly6g5b exons. Grey boxes represent intron sequences retained on each transcript. Red boxes indicate canonical isoforms. ORFs are delimited by triangles (ATG codon) and hexagons (Stop codon). Squares at the left or right, represent the presence (black) or absence (white) of expression of each transcript on the indicated tissue. Black arrows indicate Ly6g6b-Csnk2b chimeras that carry structural domains from Csnk2b and Ly6g5b, respectively. The corresponding nucleotide and protein sequences and the accession numbers (EMBL: HE864415-HE864490) are shown in Additional file 1.

Canonical Csnk2kb ORF orthologue sequences from *Homo sapiens* (NM_001320), *Macaca mulata* (XM_001112478), *Sus scrofa* (XM_001928731), *Bos taurus* (NM_001046454), *Rattus norvegicus* (NM_031021) and *Mus musculus* (NM_009975) were analysed in order to find common features among them. Comparative analysis showed a total conservation rate in protein sequence among these six species, except for *Mus musculus* which presents a unique change in position 57 (V→ E) (Figure 1A). Through RT-PCR analysis, we found five different transcripts for *Csnk2b* in *Homo sapiens*, four in *Macaca mulata*, four in *Sus scrofa*, five in *Bos taurus*, two in *Rattus norvegicus* and three in *Mus musculus* (Figure 2 and Additional file 1). Only the canonical transcript sequences were on databases, except for *Bos taurus* for which BtCsnk2b-473 was also present (see Additional file 2: Table S1). We could detect the presence of the canonical *Csnk2b* transcript in each tested species and tissues (Figure 2), and it was also the isoform expressed at the highest level (data not shown). In addition, *Csnk2b* expression also generated other transcripts (Figure 2) expressed at lower levels (data not shown), but with a remarkable specificity among the analysed tissues in these six species (Figure 2). Some of them presented quite restricted expression patterns, such as the *Macaca mulata* ones that are only expressed in lung, or the *Rattus norvegicus* one, expressed only in brain. By contrast, the variants from *Sus scrofa, Bos taurus* and *Mus musculus* are broadly expressed. In *Homo sapiens*, the isoform which retains intron 5 is widely expressed; however the other three are only expressed in liver and lung. Through AS these *CSNK2B* transcripts present exon skipping, alternative 5’ and 3’ splice site and intron retention events (Figure 2) in the different species, which would generate severely truncated or aberrant proteins by using the canonical start codon.

### *Ly6g5b* transcript analysis

Canonical *Ly6g5b* ORF orthologue sequences from *Homo sapiens* (NM_021221), *Macaca mulata* (XR_014070), *Sus scrofa* (XM_001926307), *Bos taurus* (XM_585827), *Rattus norvegicus* (NM_001001934) and *Mus musculus* (NM_148939) were compared (Figure 1B). Although some differences in amino acid sequence can be detected, a *LY-6* protein domain conservation in these *Ly6g5b* orthologues is clearly present (Figure 1B). This domain is composed of ~80 amino acids and is characterised by a conserved pattern of eight to ten cysteine residues that have a defined disulfide-bounding pattern [14]. Through RT-PCR analysis, we found four different transcripts for *Ly6g5b* in Homo sapiens, two in *Macaca mulata*, three in *Sus scrofa*, three in *Bos taurus*, four in *Rattus norvegicus* and two in *Mus musculus* (Figure 2 and Additional file 1). The majority of these transcripts were not available on databases; even the canonical sequences (see Additional file 2: Table S1). Curiously, the presence of the canonical *Ly6g5b* transcript was only detected in three of the analysed species (*Homo sapiens, Rattus norvegicus* and *Mus musculus*). Similarly to what happened for *Csnk2b*, different transcript variants are generated for *Ly6g5b* through AS. These transcripts present a remarkable specificity among the analysed tissues and species, and assuming that they could be translated into proteins starting from the first canonical start codon, only truncated or aberrant proteins could be generated by them. Nevertheless, there is an interesting feature that should be stressed, the retention of the first intron in *Ly6g5b* transcripts, giving rise to a particular isoform (exon 1, intron 1, exon 2 and exon 3) that is present in all the tissues and analysed species (Figure 2), indicating conservation, and presenting the highest expression levels (data not shown). This isoform contains a PSC after the canonical start codon, in the middle of the retained intron, and therefore should be degraded through control mechanisms like NMD [18,19]. However this seems not to be the case, as we have previously described in human [16].

### *Csnk2b-Ly6g5b* Chimeric transcript analysis

**Figure 3:**
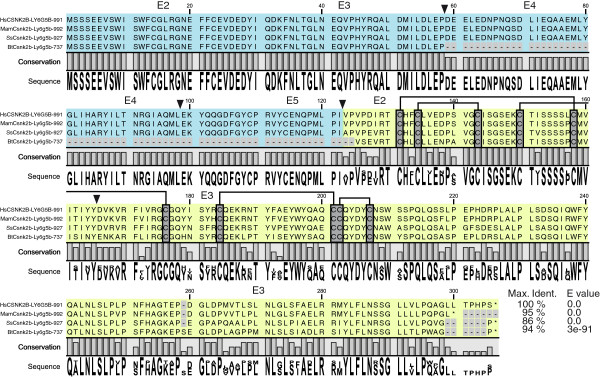
***Homo sapiens *(Hs)*, Macaca mulata *(Mam)*, Sus scrofa *(Ss) and *Bos Taurus *(Bt) Csnk2b- Ly6g5b chimeras ORF alignment.** Blue and light green colour indicates Csnk2b and Ly6g5b domains, respectively. Exon-exon junctions are indicated by inverted triangles. The conserved cysteines, characteristic for Ly-6 domain, are shown highlighted in grey and their connectivity with interconnected lines. The percentage of identity of each sequence respect to the human one is shown as well as the corresponding E values.

Through RT-PCR analysis, we found ten different *Csnk2b-Ly6g5b* chimeric transcripts in *Homo sapiens*, five in *Macaca mulata*, three in *Sus scrofa*, ten in *Bos taurus*, four in *Rattus norvegicus* and three in *Mus musculus* (Figure 2 and Additional file 1). Only human Csnk2b - Ly6g5b -1181 was found on databases (see Additional file 2: Table S1). As it happened with Csnk2b and Ly6g5b independent transcripts described above, AS seems to play an important role in generating these chimeras, and results in a set of transcripts that greatly vary in terms of composition and size. Indeed, exon skipping, intron retention and intergenic region retention events are present in these transcripts. The majority of the described chimeras (26/35) have a common characteristic: the total lack of the last exon (exon 7) of the upstream gene (*Csnk2b*) as well as the first exon of the downstream gene (*Ly6g5b*). There are also four chimeric transcripts that partially lack *Csnk2b* last exon (exon 7) (two in *Macaca mulata* and two in *Bos taurus*), one that partially maintains *Ly6g5b* first exon (in *Macaca mulata*) and four that retain the intergenic regions (three in *Rattus norvegicus* and one in *Macaca mulata*). Although chimeras function is still unknown, some authors defend that this kind of fusion might generate bi-functional proteins which would have the properties of both original proteins [23,26]. Assuming this, we determined the number of chimeric transcripts which conserved the ORF of both *Csnk2b* and *Ly6g5b* genes. We found such transcripts in *Homo sapiens*, *Macaca mulata*, *Sus scrofa* and *Bos taurus*, of which only *HsCsnk2b -* Ly6g5b *-991, MamCsnk2b-Ly6g5b-992* and *SsCsnk2b-Ly6g5b-927* (Figures 2, 3 and Additional file 1) maintain the N-terminal functional domains (alpha helices 1 to 6) from *Csnk2b* (see Figures 1A and 3) [27], such as the acidic loop (aa 55-64) and nuclear localization sequence (aa 9-14 or α1), as well as the *LY-6* structural domain (see Figures 1B and 3) [14], allowing the possibility to create potentially bi-functional proteins [26,28,29]. These particular transcripts which contain the same exon-intron structure are expressed in different tissues, but commonly in brain. *Bos taurus BtCsnk2b-Ly6g5b-737* maintains only exons 1 to 3 of *Csnk2b* and then would not encode its entire N-terminal domain [27]. We did not find this type of “bi-functional” chimeric transcripts in *Rattus norvegicus* or *Mus musculus* (Figure 2).

It is interesting to note that several (23/35) chimeric transcripts could encode *Csnk2b* truncated proteins (7/35) or with modifications in their C-terminus (16/35). The *CSNK2B* C-terminal part is involved in homodimerization and binding to CSNK2A subunit (see Figure 1) [27]. Some of these detected chimeric transcripts are generated by replacing the canonical sequence of exon 7 by sequences encoded by total or partial exon 2 or 3 of *Ly6g5b* but not corresponding to *LY-6* amino-acid sequences due to changes in the reading frames. These are chimeras *HsCsnk2b-Ly6g5b-1181, MamCsnk2b-Ly6g5b-1218, SsCsnk2b-Ly6g5b-728, MumCsnk2b-Ly6g5b-979* and *MumCsnk2b-Ly6g5b-1108,* with variable tissue distribution, except *MumCsnk2b-Ly6g5b-979* that is expressed in the four tissues. Other transcripts are altered on *Csnk2b* exon 6 or 7, lacking the zinc-finger domain, α6 and C-terminal regions of CSNK2B (see Figure 1), such as *MamCsnk2b-Ly6g5b-1141* (only expressed in brain) and *BtCsnk2b-Ly6g5b-1049* (expressed in brain and lung) which maintain the same exon-intron structure (lack of Csnk2b exon 6 and Ly6g6b exon 1), and *SsCsnk2b-Ly6g5b-538* (expressed in the four tissues) (see Figure 2). In addition, there are some chimeric transcripts that would encode a complete CSNK2B protein considering that they contain all exons (1-7) of *Csnk2b* including the stop codon and in which the Ly6g5b nucleotide sequences will act as 3´ UTRs. These are *MamCsnk2b-Ly6g5b-1331, MamCsnk2b-Ly6g5b-2338, BtCsnk2b-Ly6g5b-1239, RnCsnk2b-Ly6g5b-2050, RnCsnk2b-Ly6g5b-2275* and *RnCsnk2b-Ly6g5b-2531.* They present variable tissue distribution and exon-intron structure (see Figure 2).

### *Csnk2b*, *Ly6g5b* and *Csnk2b -Ly6g5b* Chimera protein analysis

**Figure 4:**
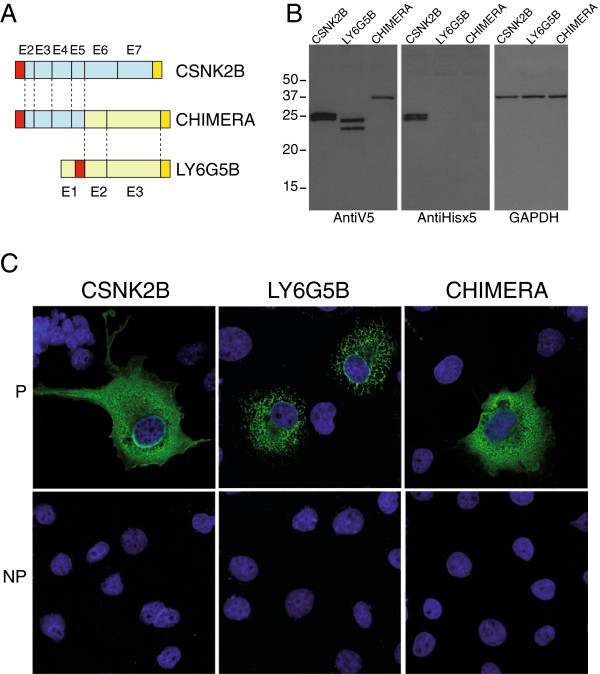
**Human CSNK2B, LY6G5B and CSNK2B-LY6G5B chimeric protein characterisation.** Schematic representation of **A**) V5 and Hisx6 double tag strategy. V5 (red box) and Hisx6 (yellow box) epitopes are shown. Exon numbers are indicated. **B**) Western-blot analysis showing in vitro expression of CSNK2B, LY6G5B and CSNK2B-LY6G5B chimera double tagged proteins. GAPDH expression is shown as internal control. **C**) Immunofluorescence experiments in permeable (P) and non permeable (NP) conditions showing CSNK2B, LY6G5B and CSNK2B-LY6G5B chimeric protein cellular distribution.

In order to analyse post-translational modifications and sub-cellular localisation of human CSNK2B, LY6G5B and CSNK2B-LY6G5B proteins, we over-expressed them in the COS7 cell line (Figure 4), using a double tag strategy. Thus, CSNK2B, LY6G5B and CSNK2B-LY6G5B proteins were C-terminally tagged by adding a Hisx6 tag. On the other hand, N-terminal V5 epitope was added upstream the first ATG to CSNK2B and CSNK2B-LY6G5B proteins, but due to the presence of a signal peptide in LY6G5B [13] and in order to tag the mature LY6G5B protein (Figure 4A) the V5 epitope tag was inserted after the signal peptide. Western blot analysis using anti-V5 or anti-PentaHis antibodies showed interesting results. Anti-V5 antibodies showed two close intense bands for *CSNK2B* protein of the estimated size (Figure 4B). These two bands could correspond to post-translational modifications of CSNK2B such as phosphorilation [30]. For *LY6G5B*, also two clear bands were also present, in agreement with previous results [14]. Interestingly, anti-V5 antibodies western-blot analysis showed a single discrete band of the predicted size for *CSNK2B-LY6G5B* protein, indicating lack of post-translational modifications.

On the other hand, anti-PentaHis antibodies showed similar pattern for *CSNK2B* protein to that showed by anti-V5 antibodies, but no signal of *LY6G5B* protein or *CSNK2B-LY6G5B* protein was detected (Figure 4B). This lack of detection on *LY6G5B* could be due to a C-terminal processing cleaving also the Tag. This cleavage signal sequence would also be present in *CSNK2B-LY6G5B* protein and for that also prone to be processed.

Cellular *CSNK2B* protein distribution has been described before in cytoplasm, nuclei, and other organelles [21]. Our results, through immunoflourescent confocal microscopy experiments by using anti-V5 antibodies under permeabilised conditions, showed mainly cytoplasmic cellular *CSNK2B* protein distribution, in agreement with previous data [21]. Under the same conditions, *LY6G5B* showed a protein distribution clearly related with ER pattern, and not extracellular staining, as previously described [14]. Here, for the first time, we show *CSNK2B-LY6G5B* protein distribution, which is quite similar to the one presented by *CSNK2B* and which clearly differs from the one of the *LY6G5B* protein.

Although the *LY6G5B* protein belongs to a GPI-anchored protein family, it has not been found to be located on the outside of the cellular membrane [14]. In addition, it is known that *CSNK2B* can be exported to the external side of the cellular membrane [31], and *CSNK2B-LY6G5B* presents *CSNK2B* domains needed for its exportation to cell surface and/or its excretion, as well as a mature Ly-6 domain (Figure 3). To know whether *CSNK2B-LY6G5B* could be on the cell surface we carried out two experimental strategies. The first one consisted of immunoflourescent confocal microscopy experiments under non-permeabilised conditions. Our results showed the absence of *CSNK2B*, *LY6G5B* as well as *CSNK2B-LY6G5B* chimeric proteins in the cell surface (Figure 4C), in COS 7 cells. The second one was to test *CSNK2B*, *LY6G5B* and/or *CSNK2B-LY6G5B* proteins presence in supernatant by western-blot experiment. It was not possible to observe expression of these proteins in the supernatant, either when loaded directly on a gel or when TCA-precipitated (data not shown).

## Discussion

After the challenge supposed by the human genome sequencing, as well as of other organisms with medical or commercial interest, the determination of the transcriptome of these species is a prerequisite for fully understanding not only their molecular biology, but also for translating this information to technical applications in medicine, pharmacology and biotechnology. In this sense, high throughput technologies supported by systematic cDNA libraries sequencing has been the main approach used for transcriptome characterisation. Thus, sequencing of random transcript cDNA clones that results in short partial sequences, known as expressed sequence tags [32] (EST) or, more recently, methods for full length isolation and sequencing of random clones from cDNA libraries have been used [33,34]. In addition, in the last few years massive sequencing technologies have also been developed for transcriptome analysis [7,24,35]. All these high throughput techniques present the advantage to generate a considerable volume of information in a relative short time, but these techniques seem to be inefficient for discovering relative rare transcripts. This affirmation is supported by recent data that suggest the existence of a wealth of transcripts which had, so far, escaped detection through systematic sequencing of cDNA libraries [36]. In a recent published work [26] combining EST and RNAseq data, it has been showed that chimeras are lowly expressed transcripts. Thus, here we present that nested RT-PCR shows to be an efficient tool to discover a number of transcripts expressed from a concrete locus, not only those highly expressed but also those expressed at lower levels. In fact, 62 of 76 transcripts detected in our experiments had never been described before. This supposes 81.5% of novel sequences, showing how powerful this technique is in order to detect transcripts from a concrete locus. In this respect, our results show that current gene and transcript annotation sets might cover only a small fraction of the total transcriptional output of the human and other organism described genomes, and in this sense, a major enforce should be taken in order to detect more transcripts of a particular organism.

A detailed human and mouse *Ly6g5b* transcript analysis has been previously described by our group [13,16]. However, here we have extended this comparative analysis among different mammalian species, showing that *Ly6g5b* first intron retention is a ubiquitous and important characteristic conserved in mammals. Its apparent capacity to escape NMD together with its conservation in the mammalian group studied points to an important role for this non-coding RNA, which should be investigated. So, we could conclude that *Ly6g5b* gene presents a double expression pattern. The first one, quite similar among tissues and species, is constituted by *Ly6g5b* transcripts with first intron retention event. The second one seems to be tissue and species specific and is constituted by canonical *Ly6g5b* transcripts with a complete Ly-6 ORF as well as aberrant and non conserved transcripts, with no common pattern distribution.

For *Csnk2b* gene expression we can also describe a double expression pattern. The first one constituted by the canonical isoform, which is quite similar among tissues and species, and the second one which seems to be tissue and species specific and is constituted by aberrant and non conserved transcripts, with no common pattern distribution.

*CSNK2B-LY6G5B* human chimerism was initially described by Calvanesse *et al* (2008) by using different human cell lines [16]. However, here we show the first comparative analysis of this TIC event using RNA from different mammalian tissues and species. Our results show that, far to be a human characteristic, *Csnk2b-Ly6g5b* chimerism is widely conserved in mammals. Its conservation among all the species analysed in this study shows how *Csnk2b-Ly6g5b* chimerism is not a trivial event. The majority of them lack the last exon of the upstream gene as well as the first exon of the downstream gene which is consistent with previous reported data [22,23]. This eliminates the stop codon as well as the molecular targets present in the 3’ UTR region of the transcripts of the upstream gene. We also agree with these authors that run-off is the most likely mechanism involved in the origin of TIC, since some chimeric transcripts detected in our study maintain the intergenic region. Other mechanisms proposed for generating chimeric transcripts, like trans-splicing, are not likely to maintain these intergenic regions.

In addition to the canonical *Csnk2b, Ly6g5* and *Csnk2b-Ly6g5b* transcripts with a coherent ORF, other transcripts detected in our study present exon skipping as well as intron retention events which allow to generate, assuming that all these transcripts could be translated into protein by using canonical start codon, truncated or aberrant proteins. Others are non-coding RNAs. The observation that there are tens of thousands of non-coding RNA (ncRNA) expressed in mammals, and that most of the genome is transcribed, confronts and contradicts the traditional protein-centric view of genetic information and genome organisation [37], [38]. Thus, there are two opposing alternatives either the bulk of the transcription which does not yield mRNAs is ‘transcriptional noise’ and/or the residue of evolutionary baggage retained or accumulated within genes, or this transcription comprises another level of expression and transaction of RNA information that is important to the evolution and developmental ontogeny of the higher organisms [39]. If one assumes that all this is transcriptional noise and that all these transcripts are the result of transcriptional machinery mistakes while it is working, they should not be distributed in a specific manner.

Some authors defend that chimerism might generate bi-functional proteins having properties from both original proteins [23]. Through different analyses of our results, we could identify chimeras which maintain the ORFs of *Csnk2b* and *Ly6g5b* susceptible to form bi-functional chimeric proteins in *Homo sapiens*, *Macaca mulata*, and *Sus scrofa*. The fact that these were not found in cow, rat and mouse does not indicate functional chimera absence in these species, since they could be present in other tissues not analysed in this study. These hypothetical new chimeric proteins all carry N-terminus domains from *Csnk2b* involved in structural aspects that are required for *Csnk2b* exportation to the cellular surface [21] and/or its regulation [27], as well as Ly-6 domain amino acid sequence [13,14] at their C-terminus. However, the chimera “bi-functional” protein will affect the juxta-dimer interface region [27] containing the zinc-finger involved in the homo-dimerisation, as well as all the C-terminal domain involved in the interaction with the *CSNK2A* subunits and the crucial last 20 amino-acids also involved in the homo-dimerisation. In addition, the Ly-6 domain, with 10 Cysteins, could not be folded as such due to the intracellular localisation. These two facts indicate that the “bi-functional” chimera would not be formed, and would only have one function: the one of CSNK2B, although possibly binding to other kinase different to CSNK2A due to the alterations produced on the C-terminal domain commented above. Other chimeras would only be affected from exon 7, containing then the juxta-dimer interface region but differing at the very end C-terminal region and not containing the Ly-6 domain sequence due to frameshifts, and probably only affecting the binding to *CSNK2A*. It has been proposed that *CSNK2B* might bind other kinases such as Ras-1 and Mos to modify their catalytic affinity in a CK2-independent fashion. The alternative C-terminal ends, generated by the chimeric transcripts, could increment the binding repertoire of *CSNK2B* to other kinases or non-kinase proteins converting *CSNK2B* in even a wider “wild-card” regulator subunit than previously proposed [27].

In addition, we have found chimeric transcripts that would encode a complete CSNK2B protein, but with different 3´UTR due to the Ly6g5b sequence, in *Macaca mulata, Bos taurus* and *Rattus norvegicus*. These transcripts could have different mRNA localisations or stabilities which could have altered protein functional implications [40,41]. It has been described that some 3´UTR contain “localization elements” or “zip codes” which target mRNAs to specific subcellular sites.

## Conclusions

Alternative splicing has an important role in *Csnk2b* and *Ly6g5b* gene expression. *Ly6g5b* intron retention and *Csnk2b-Ly6g5b* chimera transcripts are present in many tissues of different mammals. The data and analysis we have performed should serve as a valuable resource for further characterizing the possible functional role of TIC and the mechanisms that affect it.

## Methods

### Computational analysis

*Csnk2b* and *Ly6g5b* orthologue ORF sequences *Homo sapiens*, *Macaca mulata*, *Sus scrofa*, *Bos taurus*, *Rattus norvegicus* and *Mus musculus* were obtained from the National Centre for Biotechnology Information (NCBI) (http://www.ncbi.nlm.nih.gov/BLAST/) [42]. Multiple alignments of sequences were performed with ClustalW2 software (http://www.ebi.ac.uk/Tools/msa/clustalw2/) [43,44]. Protein sequences analyses were carried out using Pfam (http://www.sanger.ac.uk/resources/databases/pfam.html) [45], SMART (http://smart.embl-heidelberg.de/) and InterProScan (http://www.ebi.ac.uk/InterProScan/) [46-49] databases. The sequencing results were evaluated using the BLAST algorithm at the NCBI web page and the MultAlin alignment software (http://bioinfo.genotoul.fr/multalin/multalin.html) [50]. Primers were designed using the Primer3 software (http://www.bioinformatics.nl/cgi-bin/primer3plus/primer3plus.cgi).

### mRNA extraction and retrotranscription

*Homo sapiens*, *Macaca mulata*, *Sus scrofa*, *Bos taurus*, *Rattus norvegicus* and *Mus musculus* brain, kidney, liver and lung tissue total RNAs were obtained from BioChain® (USA) http://www.biochain.com through one of their Europe distributor "AMS" http://www.amsbio.com (UK). One μg of total RNA from each tissue was used for oligo-dT primed cDNA synthesis which was performed using the ImProm-II(TM) Reverse Transcription System (Promega) in a 20 μl reaction volume following the manufacturer's instructions.

### Nested RT-PCR

In order to detect Csnk2b, Ly6g5b and Csnk2b-Ly6g5b transcripts, first and second rounds of nested RT-PCR were performed with the primers indicated on Table 1 by using GoTaq® Green Master Mix (Promega) in a 20 μl reaction volume. For the first round of PCR 1 μl of cDNA was used in each reaction. Amplification conditions were: 95°C for 5 min followed by 40 cycles of 95°C for 30 s, 60°C for 30 s and 72°C for 90 s followed by 72°C for 10 min. For the second round of PCR, 1 μl from the first round product diluted 1/25 was used in each PCR reaction. Amplification conditions were the same as before but only 30 cycles were used. PCR products were separated by size through electrophoresis, purified from gel (Wizard® SV Gel and PCR Clean-Up System, Promega) and finally cloned into pGEM-T Easy Vector (Promega). Sequencing of PCR products and plasmids were carried out by the sequencing service of the Instituto de Investigaciones Biomédicas “Alberto Sols” (Madrid, Spain. http://www.iib.uam.es).

**Table 1:** Nested-PCR primers

		**CSNK2B**		**LY6G5B**	
		**First Round**	**Second Round**	**First Round**	**Second Round**
***H.sapiens***	**F**	HsCSNK2BE1f01	Hs-MamCSNK2BE1-2f01	HsLY6G5BE1f01	HsLY6G5BE1f02
		CGTTCCCTGGAAGTAGCAA	GCTGACGTGAAGATGAGCAG	GAGCATGGTCACAGGAAGGT	CATCTCCCCAGAATTCCAAA
	**R**	Hs-SsCSNK2BE7r02	Hs-Mam-RnCSNK2BE7r01	HsLY6G5BE3r02	Hs-MamLY6G5BE3r01
		CCCACCACAATAACGACTCC	TCAGCGAATCGTCTTGACTG	CGGAGGCCTAAGAAATCACA	TGTTTTCAGAGAGGGCAGTG
***M.mulata***	**F**	MamCsnk2bE1iso1f01	Hs-MamCsnk2bE1-2f01	MamLy6g5bE1f01	MamLy6g5bE1f02
		ACCCTCCCCAATTTCCACT	GCTGACGTGAAGATGAGCAG	TCACAGGAAGGTGGGGTTT	CGTCTCCCCAGAATTCCATA
	**R**	Mam-BtCsnk2bE7r02	Hs-Mam-RnCsnk2bE7r01	MamLy6g5bE3r02	Hs-MamLy6g5bE3r01
		TCCCACCACAATAATGACTCC	TCAGCGAATCGTCTTGACTG	CAACAGAGGGAGGCCTAAGA	TGTTTTCAGAGAGGGCAGTG
***S.scrofa***	**F**	SsCsnk2bE1f01	SsCsnk2bE1-2f01	SsLy6g5bE1f01	SsLy6g5bE1f02
		TCCTTGGAAGCAGAAACTCC	CGCTGAAGTGAAGATGAGCA	CTCAGGGATCACCCCTCTC	TCACGTGCTCGTAGGTATGC
	**R**	Hs-SsCsnk2bE7r02	SsCsnk2bE7r01	SsLy6g5bE3r02	SsLy6g5bE3r01
		CCCACCACAATAACGACTCC	GGGAATCAGCGAATTGTCTT	GAATGCTGGGGTCTTAGGG	CCGGGAAGGATGAGATGTTA
***B.taurus***	**F**	BtCsnk2bE1f01	BtCsnk2bE1-2f01	BtLy6g5bE1f01	BtLy6g5bE1f02
		GAAGCAGAAACTCCCCTTCC	CCGACGTGAAGATGAGCAG	GTCAGAACCACCCTGCAGTT	CTCTCCCCAGAAGTCCATGA
	**R**	Mam-BtCsnk2bE7r02	BtCsnk2bE7r01	BtLy6g5bE3r02	BtLy6g5bE3r01
		TCCCACCACAATAATGACTCC	GGAATCAGCGAATCGTCTTC	GGGGTGTCAGGAGTGAGATG	AATCTATCTGCGACGGGAAA
***R.norvegicus***	**F**	Rn-MumCsnk2bE1f01	Rn-MumCsnk2bE1-2f01	RnLy6g5bBE1f01	RnLy6g5bE1f02
		GTTCCTTGGAAGCACAGCTC	CCGCGGACATAAAGATGAGT	AGTGTGATCCCAGGAAGGTG	CTACTCCACGGGAGTTGCTC
	**R**	RnCsnk2bE7r02	Hs-Mam-RnCsnk2bE7r01	RnLy6g5bE3r02	RnLy6g5bE3r01
		GTGTCACAGGCAGAGGAGGT	TCAGCGAATCGTCTTGACTG	CCATGGAGAGCAGAAGGAAG	CAGAGCAGAATCTGGGAAGG
***M.musculus***	**F**	Rn-MumCsnk2bE1f01	Rn-MumCsnk2bE1-2f01	MumLy6g5bE1f01	MumLy6g5bE1f02
		GTTCCTTGGAAGCACAGCTC	CCGCGGACATAAAGATGAGT	ACTGCCTGTCAAACCCATTC	TCCCCCAAATTCCATAATGA
	R	MumCsnk2bE7r02	MumCsnk2bE7r01	MumLy6g5bE3r02	MumLy6g5bE3r01
		AGCAGAGGAATGGTGGTGTC	GTGGGCAATCAGCGAATAGT	GAGTGTTCACAGACCGCAGA	GGGGAACACATCAGGGTCTA

Name and sequence (5’-3’ orientation). F, forward primer; R, reverse primer.

### Expression constructs

The full-length coding sequence of human *CSNK2B, LY6G5B* and *CSNK2B-LY6G5B* were amplified from the previously cloned pGEM-T Easy vectors with the specific primers showed on Table 2 by using *Pfu* DNA Polymerase (Promega). Amplification conditions were: 95°C for 5 min followed by 35 cycles of 95°C for 30 s, 60°C for 30 s and 74°C for 3 min followed by 74°C for 10 min. PCR products were cloned in *Bam*HI/*Age*I cloning sites present in pcDNA3.1/V5-His-TOPO plasmid (Invitrogen), removing V5 epitope and in frame with the His tag creating *CSNK2B-*His, *CSNK2B-LY6G5B-*His and exon 2-exon 3 *LY6G5B-*His plasmids. To create a V5Tag-pcDNA3.1/Zeo vector we designed long primers containing the V5 epitope sequence (Table 2). These primers were hybridised (100°C for 4 min followed by 1 min cycles decreasing 0,5°C per cycle from 100°C to 4°C) to create the dsV5 Tag sequence, which was cloned in *Not*I*/Eco*RV cloning sites present in pcDNA3.1/Zeo(-) plasmid (Invitrogen) ending with the V5-Tag-pcDNA3.1/Zeo vector. Then, ATG-Stop *CSNK2B*-His and *CSNK2B-LY6G5B*-His and exon 2-exon 3 *LY6G5B*-His coding sequences were amplified with primers showed on Table 2 by using *Pfu* DNA Polymerase (Promega) under the same PCR conditions as above and cloned in *Bam*HI*/Hin*dIII cloning sites present in the new V5-Tag-pcDNA3.1/Zeo vector in frame with the N-terminal V5 epitope creating V5-*CSNK2B*-His, V5-*CSNK2B*-*LY6G5B*-His and V5-exon 2-exon 3 *LY6G5B*-His. Finally, LY6G5B exon 1 was amplified from LY6G5B-His plasmid using the primers showed on Table 2 and *Pfu* DNA Polymerase (Promega) under the same PCR conditions described above and cloned in *Xba*I*/Not*I cloning sites present in V5-exon 2-exon 3 LY6G5B-His plasmid to finally create an exon1-V5-exon 2-exon 3 *LY6G5B*-His vector. Sequencing of PCR products and plasmids were carried out by the sequencing service of the Instituto de Investigaciones Biomédicas “Alberto Sols” (Madrid, Spain. http://www.iib.uam.es).

**Table 2:** Expression constructs primers

*Long primers for V5 epitope cloning in pcDNA3.1/Zeo(-)*	
Name	Sequence
MCV5_F01	GGG***GCGGCCGC***ATGGGAAAGCCGATCCCAAACCCTCTATTAGGTCTGGACTCCACC***GGATCC***TGA***GATATC***GGG
MCV5_R01	CCC***GATATC***TCA***GGATCC***GGTGGAGTCCAGACCTAATAGAGGGTTTGGGATCGGCTTTCCCAT***GCGGCCGC***CCC
*Primers for CSNK2B, LY6G5B and CSNK2B-LY6G5B Chimera ORFs cloning in pcDNA3.1/V5-His-TOPO*	
Name	Sequence
HsCSNK2BORF_F01	CCC***GGATCC***ATGAGCAGCTCAGAGG
HsCSNK2BORF_R01	CCC***ACCGGT***GCGAATCGTCTTGAC
HsLY6G5BORF_F01	CCC***GGATCC***ATGAAGGTCCATATGC
HsLY6G5BORF_R01	CCC***ACCGGT***GGAAGGGTGAGGTGTC
*Primers for final construct in pcDNA3.1/Zeo(-)*	
Name	Sequence
HsCSNK2BORF_F01	CCC***GGATCC***ATGAGCAGCTCAGAGG
HsLY6G5BORFE2_F02	CCC***GGATCC***GTTCCTGTTCCCGACATC
Hisx6HindIIIR_01	CCC***AAGCTT***TCAATGGTGATGGTGATGATG
HsLY6G5BPS_F01	GGG***TCTAGA***ATGAAGGTCCATATGC
HsLY6G5BPS_R01	CCC***GCGGCCGC***TCTTTCCTACTGTGAA

Restriction enzyme target sequences are in bold and italics.

### Transfections and western blot analyses

Transfections of COS-7 cells were carried in 24-well plates, with 0.5 μg of plasmid per well, by using TransIT® COS Transfection kit (Mirus-BioNova) following the manufacturer´s instructions. For Western Blot analyses, two days after transfection, cells were harvested in Laemmli´s SDS sample buffer. Samples were resolved on SDS 12% (w/v) polyacrylamide gels and the proteins were transferred onto nitrocellulose membranes (Protran). After blocking the membrane 30 minutes in PBST (PBS-0,05% (v/v) Tween) containing 5% (w/v) skimmed milk powder (blocking solution), the blot was first incubated for 90 min either in a 1/2000 dilution of mouse anti-Hisx5 (Qiagen), a 1/10000 dilution of mouse anti-V5 (Sigma) or a 1/5000 dilution of mouse anti-GAPDH monoclonal antibodies in blocking solution, washed three times for 10 min with PBST, and then incubated for 30 min in a 1:5000 dilution of peroxidase-conjugated anti-mouse IgG antibody (Sigma) in PBST. Membrane was washed twice for 10 min with PBST followed by a final wash in PBS for 10 min. Bound antibodies were detected by ECL Plus Western Blotting (Amershan). Secreted proteins were TCA-precipitated from the culture media after the addition of 1 volume of TCA to 4 volumes of media, incubated 10 min at 4°C, washed with 200 μl cold acetone, resuspended in Laemmli´s SDS sample buffer, and analysed by SDS-PAGE followed by Western blotting, as described above.

### Immunofluorescences

Transfections were performed as described above. Two days after transfection, cells were fixed for 15 min at room temperature with 4% (v/v) paraformaldehyde in PBS. Following fixation, cells were washed three times for 15 min with PBS, treated with ammonium chloride 50 mM for 30 min and permeabilised for 20 min with PBS containing 0.1% (v/v) Triton X-100 (wash solution). Cells were blocked for 30 min in wash solution with 5% (w/v) BSA (Bovine Serum Albumin, blocking solution) and then incubated for 1 h with 1:400 dilution of mouse anti-V5 monoclonal antibody (Sigma) in blocking solution. After that, cells were washed three times for 15 min with wash solution and then incubated with a secondary antibody coupled to Alexa 488 (anti mouse IgG, Invitrogen) in a 1:500 dilution for 1 h in blocking solution. Cells were washed three times for 15 min with wash solution and nucleic acids were stained with To-Pro3 in a 1:500 dilution in blocking solution. Finally, cells were washed three times for 15 min with wash solution, mounted with Prolong Gold Antifade Reagent (Molecular Probes) and photographed with a Leika confocal microscope. Non-permeabilised immunofluorescence experiments were carried out under the same conditions described above but avoiding Triton X-100 addition.

## Abbreviations

RT-PCR: Reverse transcription polymerase chain reaction; CDS: Coding sequence; AS: Alternative splicing; MHC: Major Histocompatibility Complex; ORF: Open reading frame; PSC: Premature stop codon; NMD: Nonsense mediated decay; TIC: Transcription induced chimerism; EST: Expressed sequence tag; cDNA: Complementary DNA; mRNA: Messenger RNA; ncRNA: Non-coding RNA; TCA: Trichloroacetic acid; ER: Endoplasmic reticulum

## Competing interest

The authors declare that they have no competing interests.

## Authors’ contributions

FH-T and AR designed and carried out the experiments, and analysed and interpreted the data. FH-T drafted the manuscript. BA conceived the study, participated in its design and data interpretation, and completed the manuscript. All authors read and approved the final manuscript.

## Supplementary Material

Additional file 1 List of all the nucleotide and protein sequences of the mRNAs described on this article with their corresponding names and accession numbers. Click here for file

Additional file 2: Table S1 Table that contains all the different spliced isoforms detected in all the species studied in this work indicating whether they were previously described (deposited on databases) and, in the case they were found on databases the access number of the corresponding sequence or of the ESTs are indicated. Click here for file
