# Supplementary material for: Intron retention and transcript chimerism conserved across mammals: Ly6g5b and Csnk2b-Ly6g5b as examples
Source: BMC Genomics. 2013 Mar 22;14:199. doi: 10.1186/1471-2164-14-199 (PMC3626593; doi:10.1186/1471-2164-14-199)
Supplement: Additional file 1 — List of all the nucleotide and protein sequences of the mRNAs described on this article with their corresponding names and accession numbers. [file 1471-2164-14-199-S1.doc]

Csnk2b sequences

1.- HsCSNK2B-132 HE864443

Nucleotides

GCTGACGTGAAGATGAGCAGCTCAGAGGAGGTGTCCTGGATTTCCTGGTTCTGTGGGCTCCATCCGATGGCCTACCAGCTGCAGCTCCAAGCCGCCAGCAACTTCAAGAGCCCAGTCAAGACGATTCGCTGA

Amino acids

MSSSEEVSWISWFCGLHPMAYQLQLQAASNFKSPVKTIR*

2.-HsCSNK2B-247 HE864444

Nucleotides

GCTGACGTGAAGATGAGCAGCTCAGAGGAGGTGTCCTGGATTTCCTGGTTCTGTGGGCTCCGTGGCAATGAATTCTTCTGTGAAGTGGATGAAGACTACATCCAGGACAAATTTAATCTTACTGGACTCAATGAGCAGGTCCCTCACTACCGACAAGCTCTACGGTTTCAAGATCCATCCGATGGCCTACCAGCTGCAGCTCCAAGCCGCCAGCAACTTCAAGAGCCCAGTCAAGACGATTCGCTGA

Amino acids

MSSSEEVSWISWFCGLRGNEFFCEVDEDYIQDKFNLTGLNEQVPHYRQALRFQDPSDGLPAAAPSRQQLQEPSQDDSL

3.-HsCSNK2B-318 HE864445

Nucleotides

GCTGACGTGAAGATGAGCAGCTCAGAGGAGGTGTCCTGGATTTCCTGGTTCTGTGGGCTCCGTGGCAATGAATTCTTCTGTGAAGTGGATGAAGACTACATCCAGGACAAATTTAATCTTACTGGACTCAATGAGCAGGTCCCTCACTACCGACAAGCTCTAGACATGATCTTGGACCTGGAGCCTGATGAAGAACTGGAAGACAACCCCAACCAGTTTGTGCCCAGGCTCTACGGTTTCAAGATCCATCCGATGGCCTACCAGCTGCAGCTCCAAGCCGCCAGCAACTTCAAGAGCCCAGTCAAGACGATTCGCTGA

Amino acids

MSSSEEVSWISWFCGLRGNEFFCEVDEDYIQDKFNLTGLNEQVPHYRQALDMILDLEPDEELEDNPNQFVPRLYGFKIHPMAYQLQLQAASNFKSPVKTIR*

4.-HsCSNK2B-660 HE864446

Nucleotides

GCTGACGTGAAGATGAGCAGCTCAGAGGAGGTGTCCTGGATTTCCTGGTTCTGTGGGCTCCGTGGCAATGAATTCTTCTGTGAAGTGGATGAAGACTACATCCAGGACAAATTTAATCTTACTGGACTCAATGAGCAGGTCCCTCACTACCGACAAGCTCTAGACATGATCTTGGACCTGGAGCCTGATGAAGAACTGGAAGACAACCCCAACCAGAGTGACCTGATTGAGCAGGCAGCCGAGATGCTTTATGGATTGATCCACGCCCGCTACATCCTTACCAACCGTGGCATCGCCCAGATGTTGGAAAAGTACCAGCAAGGAGACTTTGGTTACTGTCCTCGTGTGTACTGTGAGAACCAGCCAATGCTTCCCATTGGCCTTTCAGACATCCCAGGTGAAGCCATGGTGAAGCTCTACTGCCCCAAGTGCATGGATGTGTACACACCCAAGTCATCAAGACACCATCACACGGATGGCGCCTACTTCGGCACTGGTTTCCCTCACATGCTCTTCATGGTGCATCCCGAGTACCGGCCCAAGAGACCTGCCAACCAGTTTGTGCCCAGGCTCTACGGTTTCAAGATCCATCCGATGGCCTACCAGCTGCAGCTCCAAGCCGCCAGCAACTTCAAGAGCCCAGTCAAGACGATTCGCTGA

Amino acids

MSSSEEVSWISWFCGLRGNEFFCEVDEDYIQDKFNLTGLNEQVPHYRQALDMILDLEPDEELEDNPNQSDLIEQAAEMLYGLIHARYILTNRGIAQMLEKYQQGDFGYCPRVYCENQPMLPIGLSDIPGEAMVKLYCPKCMDVYTPKSSRHHHTDGAYFGTGFPHMLFMVHPEYRPKRPANQFVPRLYGFKIHPMAYQLQLQAASNFKSPVKTIR*

5.-HsCSNK2B-806 HE864447

Nucleotides

GCTGACGTGAAGATGAGCAGCTCAGAGGAGGTGTCCTGGATTTCCTGGTTCTGTGGGCTCCGTGGCAATGAATTCTTCTGTGAAGTGGATGAAGACTACATCCAGGACAAATTTAATCTTACTGGACTCAATGAGCAGGTCCCTCACTACCGACAAGCTCTAGACATGATCTTGGACCTGGAGCCTGATGAAGAACTGGAAGACAACCCCAACCAGAGTGACCTGATTGAGCAGGCAGCCGAGATGCTTTATGGATTGATCCACGCCCGCTACATCCTTACCAACCGTGGCATCGCCCAGATGTTGGAAAAGTACCAGCAAGGAGACTTTGGTTACTGTCCTCGTGTGTACTGTGAGAACCAGCCAATGCTTCCCATTGGTGAGTGTTGAAGAAGGGAAAGGAAAGCACCGTGTGGCAGTCTTATGGGAAGGAGTTGGGGCTCAACACATTGGAGCCTGAGTCCTGAGGGGAGGTTAGGTAGGAATAGGGGGATACCTGGCCTGCTGAGTCTGGCTGTCTCCCAGGCCTTTCAGACATCCCAGGTGAAGCCATGGTGAAGCTCTACTGCCCCAAGTGCATGGATGTGTACACACCCAAGTCATCAAGACACCATCACACGGATGGCGCCTACTTCGGCACTGGTTTCCCTCACATGCTCTTCATGGTGCATCCCGAGTACCGGCCCAAGAGACCTGCCAACCAGTTTGTGCCCAGGCTCTACGGTTTCAAGATCCATCCGATGGCCTACCAGCTGCAGCTCCAAGCCGCCAGCAACTTCAAGAGCCCAGTCAAGACGATTCGCTGA

Amino acids

MSSSEEVSWISWFCGLRGNEFFCEVDEDYIQDKFNLTGLNEQVPHYRQALDMILDLEPDEELEDNPNQSDLIEQAAEMLYGLIHARYILTNRGIAQMLEKYQQGDFGYCPRVYCENQPMLPIGEC*

1.-MamCsnk2b-205 HE864462

Nucleotides

GCTGACGTGAAGATGAGCAGCTCAGAGGAGGTGTCCTGGATTTCCTGGTTCTGTGGGCTCCGTGGCAATGAATTCTTCTGTGAAGTGGATGAAGACTACATCCAGGACAAATTTAATCTTACTGGACTCAATGAGCAGGTTCCTCACTATCGACAAGCTCCAAGCCGCCAGCAACTTCAAGAGCCCAGTCAAGACGATTCGCTGA

Amino acids

MSSSEEVSWISWFCGLRGNEFFCEVDEDYIQDKFNLTGLNEQVPHYRQAPSRQQLQEPSQDDSL

2.-MamCsnk2b-238 HE864463

Nucleotides

GCTGACGTGAAGATGAGCAGCTCAGAGGAGGTGTCCTGGATTTCCTGGTTCTGTGGGCTCCGTGGCAATGAATTCTTCTGTGAAGTGGATGAAGACTACATCCAGGACAAATTTAATCTTACTGGACTCAATGAGCAGGTTCCTCACTATCGACAAGCTCAAGATTCATCCGATGGCCTACCAGCTGCAGCTCCAAGCCGCCAGCAACTTCAAGAGCCCAGTCAAGACGATTCGCTGA

Amino acids

MSSSEEVSWISWFCGLRGNEFFCEVDEDYIQDKFNLTGLNEQVPHYRQAQDSSDGLPAAAPSRQQLQEPSQDDSL

3.-MamCsnk2b-274 HE864464

Nucleotides

GCTGACGTGAAGATGAGCAGCTCAGAGGAGGTGTCCTGGATTTCCTGGTTCTGTGGGCTCCGTGGCAATGAATTCTTCTGTGAAGTGGATGAAGACTACATCCAGGACAAATTTAATCTTACTGGACTCAATGAGCAGGTTCCTCACTATCGACAAGAGACCCGCCAACCAGTTTGTGCCCAGGCTCTACGGTTTCAAGATTCATCCGATGGCCTACCAGCTGCAGCTCCAAGCCGCCAGCAACTTCAAGAGCCCAGTCAAGACGATTCGCTGA

Amino acids

MSSSEEVSWISWFCGLRGNEFFCEVDEDYIQDKFNLTGLNEQVPHYRQETRQPVCAQALRFQDSSDGLPAAAPSRQQLQEPSQDDSL

4.-MamCsnk2b-660 HE864465

Nucleotides

GCTGACGTGAAGATGAGCAGCTCAGAGGAGGTGTCCTGGATTTCCTGGTTCTGTGGGCTCCGTGGCAATGAATTCTTCTGTGAAGTGGATGAAGACTACATCCAGGACAAATTTAATCTTACTGGACTCAATGAGCAGGTTCCTCACTATCGACAAGCTCTAGACATGATCTTGGACCTGGAGCCTGATGAAGAACTGGAAGACAACCCCAACCAGAGTGACCTGATTGAGCAGGCTGCCGAGATGCTTTATGGATTGATCCACGCCCGCTACATCCTTACCAACCGTGGCATTGCCCAGATGTTGGAAAAGTACCAGCAGGGAGACTTTGGTTACTGTCCTCGTGTGTACTGTGAGAACCAGCCAATGCTTCCCATCGGCCTTTCAGACATCCCAGGTGAAGCCATGGTGAAGCTCTACTGCCCCAAGTGCATGGATGTGTACACACCCAAGTCATCGAGACACCATCACACGGATGGCGCCTACTTTGGCACTGGTTTCCCTCACATGCTCTTCATGGTGCATCCCGAGTACCGGCCCAAGAGACCCGCCAACCAGTTTGTGCCCAGGCTCTACGGTTTCAAGATTCATCCGATGGCCTACCAGCTGCAGCTCCAAGCCGCCAGCAACTTCAAGAGCCCAGTCAAGACGATTCGCTGA

Amino acids

MSSSEEVSWISWFCGLRGNEFFCEVDEDYIQDKFNLTGLNEQVPHYRQALDMILDLEPDEELEDNPNQSDLIEQAAEMLYGLIHARYILTNRGIAQMLEKYQQGDFGYCPRVYCENQPMLPIGLSDIPGEAMVKLYCPKCMDVYTPKSSRHHHTDGAYFGTGFPHMLFMVHPEYRPKRPANQFVPRLYGFKIHPMAYQLQLQAASNFKSPVKTIR*

1.-SsCsnk2b-204 HE864473

Nucleotides

CGCTGAAGTGAAGATGAGCAGCTCAGAGGAGGTGTCCTGGATTTCCTGGTTCTGTGGGCTCCGTGGCAATGAATTCTTCTGTGAAGTGGATGAAGACTACATCCAGGACAAATTCAATCTCACTGGACTCAACGAGCAGGTGCCTCATTATCGACAAGCCGCCAGCAACTTCAAGAGCCCGGTCAAGACAATTCGCTGATTCCC

Amino acids

MSSSEEVSWISWFCGLRGNEFFCEVDEDYIQDKFNLTGLNEQVPHYRQAASNFKSPVKTIR*

2.-SsCsnk2b-324 HE864474

Nucleotides

CGCTGAAGTGAAGATGAGCAGCTCAGAGGAGGTGTCCTGGATTTCCTGGTTCTGTGGGCTCCGTGGCAATGAATTCTTCTGTGAAGTGGATGAAGACTACATCCAGGACAAATTCAATCTCACTGGACTCAACGAGCAGGTGCCTCATTATCGACAAGCCCTAGACATGATCTTGGACCTGGAACCTGATGAGGAGCTGGAAGACAACCCCAACCAGTTTGTGCCCAGGCTCTACGGTTTCAAGATCCATCCAATGGCCTACCAGCTGCAGCTCCAAGCCGCCAGCAACTTCAAGAGCCCGGTCAAGACAATTCGCTGATTCCC

Amino acids

MSSSEEVSWISWFCGLRGNEFFCEVDEDYIQDKFNLTGLNEQVPHYRQALDMILDLEPDEELEDNPNQFVPRLYGFKIHPMAYQLQLQAASNFKSPVKTIR*

3.-SsCsnk2b-476 HE864475

Nucleotides

CGCTGAAGTGAAGATGAGCAGCTCAGAGGAGGTGTCCTGGATTTCCTGGTTCTGTGGGCTCCGTGGCAATGAATTCTTCTGTGAAGTGGATGAAGACTACATCCAGGACAAATTCAATCTCACTGGACTCAACGAGCAGGTGCCTCATTATCGACAAGCCCTAGACATGATCTTGGACCTGGAACCTGATGAGGAGCTGGAAGACAACCCCAACCAGAGTGACCTGATTGAGCAGGCAGCCGAAATGCTCTATGGATTGATCCACGCCCGCTATATCCTCACCAACCGTGGCATCGCCCAGATGTTGGAAAAGTACCAGCAGGGAGACTTTGGCTACTGTCCCCGTGTGTACTGTGAGAACCAGCCAATGCTTCCCATCGGCTCTACGGTTTCAAGATCCATCCAATGGCCTACCAGCTGCAGCTCCAAGCCGCCAGCAACTTCAAGAGCCCGGTCAAGACAATTCGCTGATTCCC

Amino acids

MSSSEEVSWISWFCGLRGNEFFCEVDEDYIQDKFNLTGLNEQVPHYRQALDMILDLEPDEELEDNPNQSDLIEQAAEMLYGLIHARYILTNRGIAQMLEKYQQGDFGYCPRVYCENQPMLPIGSTVSRSIQWPTSCSSKPPATSRARSRQFADS

4.-SsCsnk2b-666 HE864476

Nucleotides

CGCTGAAGTGAAGATGAGCAGCTCAGAGGAGGTGTCCTGGATTTCCTGGTTCTGTGGGCTCCGTGGCAATGAATTCTTCTGTGAAGTGGATGAAGACTACATCCAGGACAAATTCAATCTCACTGGACTCAACGAGCAGGTGCCTCATTATCGACAAGCCCTAGACATGATCTTGGACCTGGAACCTGATGAGGAGCTGGAAGACAACCCCAACCAGAGTGACCTGATTGAGCAGGCAGCCGAAATGCTCTATGGATTGATCCACGCCCGCTATATCCTCACCAACCGTGGCATCGCCCAGATGTTGGAAAAGTACCAGCAGGGAGACTTTGGCTACTGTCCCCGTGTGTACTGTGAGAACCAGCCAATGCTTCCCATCGGCCTTTCGGACATCCCAGGCGAGGCCATGGTGAAGCTCTACTGCCCCAAGTGCATGGACGTGTACACGCCCAAGTCATCGAGGCACCACCACACGGATGGCGCCTACTTCGGCACCGGTTTCCCTCACATGCTCTTCATGGTGCACCCCGAGTACCGGCCCAAACGGCCTGCCAACCAGTTTGTGCCCAGGCTCTACGGTTTCAAGATCCATCCAATGGCCTACCAGCTGCAGCTCCAAGCCGCCAGCAACTTCAAGAGCCCGGTCAAGACAATTCGCTGATTCCC

Amino acids

MSSSEEVSWISWFCGLRGNEFFCEVDEDYIQDKFNLTGLNEQVPHYRQALDMILDLEPDEELEDNPNQSDLIEQAAEMLYGLIHARYILTNRGIAQMLEKYQQGDFGYCPRVYCENQPMLPIGLSDIPGEAMVKLYCPKCMDVYTPKSSRHHHTDGAYFGTGFPHMLFMVHPEYRPKRPANQFVPRLYGFKIHPMAYQLQLQAASNFKSPVKTIR*

1.-BtCsnk2b-191 HE864483

Nucleotides

CCGACGTGAAGATGAGCAGCTCAGAGGAGGTGTCCTGGATTTCCTGGTTCTGTGGGCTCCGTGGCAATGAATTCTTCTGTGAGGTGGATGAAGACTATATCCAGGACAAATTCAATCTCACTGGACTCAATGAGCAGGTGCCTCACTATCGACAAGCTTTAAGAGCCCAGTGAAGACGATTCGCTGATTCC

Amino acids

MSSSEEVSWISWFCGLRGNEFFCEVDEDYIQDKFNLTGLNEQVPHYRQALRAQ*

2.-BtCsnk2b-242 HE864484

Nucleotides

CCGACGTGAAGATGAGCAGCTCAGAGGAGGTGTCCTGGATTTCCTGGTTCTGTGGGCTCCGTGGCAATGAATTCTTCTGTGAGGTGGATGAAGACTATATCCAGGACAAATTCAATCTCACTGGACTCAATGAGCAGGTGCCTCACTATCGACAAGCTTCAAGATCCATCCAATGGCCTACCAGCTGCAGCTCCAAGCCGCCAGCAACTTCAAGAGCCCAGTGAAGACGATTCGCTGATTCC

Amino acids

MSSSEEVSWISWFCGLRGNEFFCEVDEDYIQDKFNLTGLNEQVPHYRQASRSIQWPTSCSSKPPATSRAQ*

3.-BtCsnk2b-281 HE864485

Nucleotides

CCGACGTGAAGATGAGCAGCTCAGAGGAGGTGTCCTGGATTTCCTGGTTCTGTGGGCTCCGTGGCAATGAATTCTTCTGTGAGGTGGATGAAGACTATATCCAGGACAAATTCAATCTCACTGGACTCAATGAGCAGGTGCCTCACTATCGACAAGCTTTAGACATGATCTTGGACCTGGAGCCTGGCTTTACGGTTTCAAGATCCATCCAATGGCCTACCAGCTGCAGCTCCAAGCCGCCAGCAACTTCAAGAGCCCAGTGAAGACGATTCGCTGATTCC

Amino acids

MSSSEEVSWISWFCGLRGNEFFCEVDEDYIQDKFNLTGLNEQVPHYRQALDMILDLEPGFTVSRSIQWPTSCSSKPPATSRAQ*

4.- BtCsnk2b-473 HE864486

Nucleotides

CCGACGTGAAGATGAGCAGCTCAGAGGAGGTGTCCTGGATTTCCTGGTTCTGTGGGCTCCGTGGCAATGAATTCTTCTGTGAGGTGGATGAAGACTATATCCAGGACAAATTCAATCTCACTGGACTCAATGAGCAGGTGCCTCACTATCGACAAGCTTTAGACATGATCTTGGACCTGGAGCCTGATGAGGAGCTGGAGGACAACCCCAACCAGAGTGACCTGATTGAGCAAGCAGCTGAAATGCTCTATGGATTGATCCACGCCCGCTATATCCTCACCAACCGTGGCATTGCCCAGATGTTGGAAAAGTACCAGCAGGGAGACTTTGGATACTGTCCCCGTGTGTACTGTGAGAACCAGCCAATGCTTCCCATCGGCTTTACGGTTTCAAGATCCATCCAATGGCCTACCAGCTGCAGCTCCAAGCCGCCAGCAACTTCAAGAGCCCAGTGAAGACGATTCGCTGATTCC

Amino acids

MSSSEEVSWISWFCGLRGNEFFCEVDEDYIQDKFNLTGLNEQVPHYRQALDMILDLEPDEELEDNPNQSDLIEQAAEMLYGLIHARYILTNRGIAQMLEKYQQGDFGYCPRVYCENQPMLPIGFTVSRSIQWPTSCSSKPPATSRAQ*

5.- BtCsnk2b-663 HE864487

Nucleotides

CCGACGTGAAGATGAGCAGCTCAGAGGAGGTGTCCTGGATTTCCTGGTTCTGTGGGCTCCGTGGCAATGAATTCTTCTGTGAGGTGGATGAAGACTATATCCAGGACAAATTCAATCTCACTGGACTCAATGAGCAGGTGCCTCACTATCGACAAGCTTTAGACATGATCTTGGACCTGGAGCCTGATGAGGAGCTGGAGGACAACCCCAACCAGAGTGACCTGATTGAGCAAGCAGCTGAAATGCTCTATGGATTGATCCACGCCCGCTATATCCTCACCAACCGTGGCATTGCCCAGATGTTGGAAAAGTACCAGCAGGGAGACTTTGGATACTGTCCCCGTGTGTACTGTGAGAACCAGCCAATGCTTCCCATCGGCCTTTCAGACATCCCAGGTGAGGCCATGGTGAAGCTCTACTGCCCCAAGTGCATGGACGTGTACACACCCAAGTCATCGAGGCACCACCACACGGATGGCGCCTACTTCGGCACCGGTTTCCCTCACATGCTCTTCATGGTGCACCCCGAGTACCGGCCCAAGAGGCCCGCCAACCAGTTTGTGCCCAGGCTTTACGGTTTCAAGATCCATCCAATGGCCTACCAGCTGCAGCTCCAAGCCGCCAGCAACTTCAAGAGCCCAGTGAAGACGATTCGCTGATTCC

Amino acids

MSSSEEVSWISWFCGLRGNEFFCEVDEDYIQDKFNLTGLNEQVPHYRQALDMILDLEPDEELEDNPNQSDLIEQAAEMLYGLIHARYILTNRGIAQMLEKYQQGDFGYCPRVYCENQPMLPIGLSDIPGEAMVKLYCPKCMDVYTPKSSRHHHTDGAYFGTGFPHMLFMVHPEYRPKRPANQFVPRLYGFKIHPMAYQLQLQAASNFKSPVKTIR*

1.-RnCsnk2b-280 HE864425

Nucleotides

CCGCGGACATAAAGATGAGTAGCTCTGAGGAGGTGTCCTGGATTTCCTGGTTCTGTGGGCTCCGTGGTAATGAATTCTTCTGTGAGGTGGATGAAGACTACATCCAGGACAAATTTAATCTTACTGGACTCAATGAGCAGGTGCCTCACTATCGACAAGCCCTAGACATGATCTTGGACCTGGAACCTGGCTCTACGGTTTCAAGATCCATCCAATGGCCTACCAGCTGCAGCTCCAAGCCGCCAGCAACTTCAAGAGCCCAGTCAAGACGATTCGCTGA

Amino acids

MSSSEEVSWISWFCGLRGNEFFCEVDEDYIQDKFNLTGLNEQVPHYRQALDMILDLEPGSTVSRSIQWPTSCSSKPPATSRAQSRRFA

2.-RnCsnk2b-662 HE864426

Nucleotides

CCGCGGACATAAAGATGAGTAGCTCTGAGGAGGTGTCCTGGATTTCCTGGTTCTGTGGGCTCCGTGGTAATGAATTCTTCTGTGAGGTGGATGAAGACTACATCCAGGACAAATTTAATCTTACTGGACTCAATGAGCAGGTGCCTCACTATCGACAAGCCCTAGACATGATCTTGGACCTGGAACCTGATGAAGAGCTGGAAGACAACCCCAACCAGAGTGACTTGATTGAGCAGGCGGCCGAGATGCTCTATGGGTTGATCCACGCCCGCTACATCCTCACCAACCGGGGCATTGCACAAATGTTGGAAAAGTACCAGCAAGGAGACTTTGGCTACTGTCCTCGAGTATACTGTGAGAACCAGCCGATGCTTCCCATCGGCCTTTCGGACATCCCAGGAGAGGCCATGGTGAAGCTCTACTGCCCCAAGTGCATGGACGTGTACACACCCAAGTCCTCTAGGCACCACCACACGGATGGCGCATACTTCGGCACTGGTTTCCCTCACATGCTCTTCATGGTGCATCCCGAGTACCGGCCCAAGCGGCCGGCCAACCAGTTTGTGCCCAGGCTCTACGGTTTCAAGATCCATCCAATGGCCTACCAGCTGCAGCTCCAAGCCGCCAGCAACTTCAAGAGCCCAGTCAAGACGATTCGCTGA

Amino acids

MSSSEEVSWISWFCGLRGNEFFCEVDEDYIQDKFNLTGLNEQVPHYRQALDMILDLEPDEELEDNPNQSDLIEQAAEMLYGLIHARYILTNRGIAQMLEKYQQGDFGYCPRVYCENQPMLPIGLSDIPGEAMVKLYCPKCMDVYTPKSSRHHHTDGAYFGTGFPHMLFMVHPEYRPKRPANQFVPRLYGFKIHPMAYQLQLQAASNFKSPVKTIR*

1.-MumCsnk2b-132 HE864435

Nucleotides

CCGCGGACATAAAGATGAGTAGCTCTGAGGAGGTGTCCTGGATTTCCTGGTTCTGTGGGCTCCGTGGTAATGAATTCTTCTGTGAGGTGGATGAAGACTACATCCAGTCAAGACTATTCGCTGATTGCCCAC

Amino acids

MSSSEEVSWISWFCGLRGNEFFCEVDEDYIQSRLFADCP

2.-MumCsnk2b-670 HE864436

Nucleotides

CCGCGGACATAAAGATGAGTAGCTCTGAGGAGGTGTCCTGGATTTCCTGGTTCTGTGGGCTCCGTGGTAATGAATTCTTCTGTGAGGTGGATGAAGACTACATCCAGGACAAATTTAATCTTACTGGACTCAATGAGCAGGTGCCTCACTATCGACAAGCTCTGGACATGATCTTAGACCTGGTACCTGATGAAGAGCTGGAAGACAACCCCAACCAGAGCGACTTGATCGAACAGGCAGCTGAGATGCTTTATGGGTTGATCCACGCCCGCTACATCCTCACCAACCGAGGCATCGCACAAATGTTGGAAAAGTACCAGCAGGGAGACTTTGGCTACTGTCCTCGTGTATACTGTGAGAACCAGCCAATGCTTCCTATCGGCCTTTCAGACATCCCAGGCGAGGCCATGGTGAAACTCTACTGCCCCAAGTGCATGGACGTGTACACACCCAAGTCCTCCAGACACCACCACACGGACGGCGCATACTTCGGCACTGGTTTCCCTCACATGCTCTTCATGGTGCATCCAGAGTACCGGCCCAAGCGACCTGCCAACCAGTTTGTACCCAGGCTCTATGGTTTCAAGATCCATCCAATGGCTTACCAGCTGCAGCTCCAAGCCGCCAGCAACTTCAAGAGCCCAGTCAAGACTATTCGCTGATTGCCCAC

Amino acids

MSSSEEVSWISWFCGLRGNEFFCEVDEDYIQDKFNLTGLNEQVPHYRQALDMILDLVPDEELEDNPNQSDLIEQAAEMLYGLIHARYILTNRGIAQMLEKYQQGDFGYCPRVYCENQPMLPIGLSDIPGEAMVKLYCPKCMDVYTPKSSRHHHTDGAYFGTGFPHMLFMVHPEYRPKRPANQFVPRLYGFKIHPMAYQLQLQAASNFKSPVKTIR*

3.-MumCsnk2b-780 HE864437

Nucleotides

CCGCGGACATAAAGATGAGTAGCTCTGAGGAGGTGTCCTGGATTTCCTGGTTCTGTGGGCTCCGTGGTAATGAATTCTTCTGTGAGGTGGATGAAGACTACATCCAGGACAAATTTAATCTTACTGGACTCAATGAGCAGGTGCCTCACTATCGACAAGCTCTGGACATGATCTTAGACCTGGAACCTGATGAAGAGCTGGAAGACAACCCCAACCAGAGCGACTTGATCGAACAGGCAGCTGAGATGCTTTATGGGTTGATCCACGCCCGCTACATCCTCACCAACCGAGGCATCGCACAAATGTTGGAAAAGTACCAGCAGGGAGACTTTGGCTACTGTCCTCGTGTATACTGTGAGAACCAGCCAATGCTTCCTATCGGTGAGTGTtgaAAGCCAGAAGCTGCCTTCCTGTACTCAGTGCCCGAGTCCTTTGGGAGGTTGGGGAGCGCTAGGGATACCTGGTCTGCAGTCTGGCTGTCTATCTCCCAGGCCTTTCAGACATCCCAGGCGAGGCCATGGTGAAACTCTACTGCCCCAAGTGCATGGACGTGTACACACCCAAGTCCTCCAGACACCACCACACGGACGGCGCATACTTCGGCACTGGTTTCCCTCACATGCTCTTCATGGTGCATCCAGAGTACCGGCCCAAGCGACCTGCCAACCAGTTTGTACCCAGGCTCTATGGTTTCAAGATCCATCCAATGGCTTACCAGCTGCAGCTCCAAGCCGCCAGCAACTTCAAGAGCCCAGTCAAGACTATTCGCTGATTGCCCAC

Amino acids

MSSSEEVSWISWFCGLRGNEFFCEVDEDYIQDKFNLTGLNEQVPHYRQALDMILDLEPDEELEDNPNQSDLIEQAAEMLYGLIHARYILTNRGIAQMLEKYQQGDFGYCPRVYCENQPMLPIGEC*

Ly6g5b sequences

1.-HsLY6G5B-288 HE864448

Nucleotides

CATCTCCCCAGAATTCCAAAATGAAGGTCCATATGCTTGTAGGTGTGCTGCCCCTCCCCAATTTCCATGCTGGGACGGAGCCTGATGGCCTGGACCCCATGGTCACACTGTCCCTGAACCTGGGCTTGTCTTTTGCTGAGCTGCGCCGCATGTACTTGTTCCTCAATAGTTCAGGACTTTTGGTTCTTCCCCAGGCTGGACTCTTGACACCTCACCCTTCCTGAATTCCACAGTGCAAATATCTTTCTGTAACACCCTCAGCATCCTGCACTGCCCTCTCTGAAAACA

Amino acids

MKVHMLVGVLPLPNFHAGTEPDGLDPMVTLSLNLGLSFAELRRMYLFLNSSGLLVLPQAGLLTPHPS*

2.-HsLY6G5B-452 HE864449

Nucleotides

CATCTCCCCAGAATTCCAAAATGAAGGTCCATATGCTTGTAGGTGTGCTGGTCATGGTGGGCTTCACAGTAGGAAAGGGTAAGTGGGGCCCAGGGGCAGGGAGGGAGGAAGGGGTAACTGAGTCCAGGAAGGGGGTGGAGCGTGGCCATGGATAATCGGGCTTCCTACTGGCCCAGGGTATTTGAGAGTGACCCAGTGCCTCCATCCCTCCTTCTGCCTCCCCAATTTCCATGCTGGGACGGAGCCTGATGGCCTGGACCCCATGGTCACACTGTCCCTGAACCTGGGCTTGTCTTTTGCTGAGCTGCGCCGCATGTACTTGTTCCTCAATAGTTCAGGACTTTTGGTTCTTCCCCAGGCTGGACTCTTGACACCTCACCCTTCCTGAATTCCACAGTGCAAATATCTTTCTGTAACACCCTCAGCATCCTGCACTGCCCTCTCTGAAAACA

Amino acids

MKVHMLVGVLVMVGFTVGKGKWGPGAGREEGVTESRKGVERGHG*

3.-HsLY6G5B-690 HE864450

Nucleotides

CATCTCCCCAGAATTCCAAAATGAAGGTCCATATGCTTGTAGGTGTGCTGGTCATGGTGGGCTTCACAGTAGGAAAGGTTCCTGTTCCCGACATCCGGACGTGCCACTTCTGCCTCGTAGAAGACCCTTCTGTAGGATGCATTTCAGGCTCAGAGAAGTGTACCATCAGCAGCTCATCCCTGTGCATGGTGATCACCATCTATTATGATGTCAAGGTTCGCTTCATCGTTCGAGGCTGTGGACAGTACATTTCCTACCGCTGCCAAGAAAAACGCAACACCTACTTTGCAGAGTACTGGTATCAGGCCCAGTGCTGTCAGTACGATTATTGCAACTCCTGGTCAAGCCCCCAACTCCAGAGCTCTCTGCCGGAGCCCCATGACAGGCCCCTGGCCCTGCCTCTGTCTGACTCCCAGATTCAGTGGTTCTACCAGGCCCTGAACCTCTCCCTGCCCCTCCCCAATTTCCATGCTGGGACGGAGCCTGATGGCCTGGACCCCATGGTCACACTGTCCCTGAACCTGGGCTTGTCTTTTGCTGAGCTGCGCCGCATGTACTTGTTCCTCAATAGTTCAGGACTTTTGGTTCTTCCCCAGGCTGGACTCTTGACACCTCACCCTTCCTGAATTCCACAGTGCAAATATCTTTCTGTAACACCCTCAGCATCCTGCACTGCCCTCTCTGAAAACA

Amino acids

MKVHMLVGVLVMVGFTVGKVPVPDIRTCHFCLVEDPSVGCISGSEKCTISSSSLCMVITIYYDVKVRFIVRGCGQYISYRCQEKRNTYFAEYWYQAQCCQYDYCNSWSSPQLQSSLPEPHDRPLALPLSDSQIQWFYQALNLSLPLPNFHAGTEPDGLDPMVTLSLNLGLSFAELRRMYLFLNSSGLLVLPQAGLLTPHPS*

4.-HsLY6G5B-837 HE864451

Nucleotides

CATCTCCCCAGAATTCCAAAATGAAGGTCCATATGCTTGTAGGTGTGCTGGTCATGGTGGGCTTCACAGTAGGAAAGGGTAAGTGGGGCCCAGGGGCAGGGAGGGAGGAAGGGGTAACTGAGTCCAGGAAGGGGGTGGAGCGTGGCCATGGATAATCGGGCTTCCTACTGGCCCAGGGTATTTGAGAGTGACCCAGTGCCTCCATCCCTCCTTCTGCCTCCCCAGTTCCTGTTCCCGACATCCGGACGTGCCACTTCTGCCTCGTAGAAGACCCTTCTGTAGGATGCATTTCAGGCTCAGAGAAGTGTACCATCAGCAGCTCATCCCTGTGCATGGTGATCACCATCTATTATGATGTCAAGGTTCGCTTCATCGTTCGAGGCTGTGGACAGTACATTTCCTACCGCTGCCAAGAAAAACGCAACACCTACTTTGCAGAGTACTGGTATCAGGCCCAGTGCTGTCAGTACGATTATTGCAACTCCTGGTCAAGCCCCCAACTCCAGAGCTCTCTGCCGGAGCCCCATGACAGGCCCCTGGCCCTGCCTCTGTCTGACTCCCAGATTCAGTGGTTCTACCAGGCCCTGAACCTCTCCCTGCCCCTCCCCAATTTCCATGCTGGGACGGAGCCTGATGGCCTGGACCCCATGGTCACACTGTCCCTGAACCTGGGCTTGTCTTTTGCTGAGCTGCGCCGCATGTACTTGTTCCTCAATAGTTCAGGACTTTTGGTTCTTCCCCAGGCTGGACTCTTGACACCTCACCCTTCCTGAATTCCACAGTGCAAATATCTTTCTGTAACACCCTCAGCATCCTGCACTGCCCTCTCTGAAAACA

Amino acids

MKVHMLVGVLVMVGFTVGKGKWGPGAGREEGVTESRKGVERGHG*

1.-MamLy6g5b-325 HE864466

Nucleotides

CGTCTCCCCAGAATTCCATAATGAAGGCCCATATACTTGTACGTGTGCTGGTCATGGTGGGCTTCACAGTGGGAAAGGGTAAGTGGGGCCCAGGGGCAGGGAGGGAGGAAGGGGTAATTGAGTTCAGGAAGGGGGTGGAACGTGGCCATGGATACTCGGGCTTGTCTTTTGCTGAGCTGCGCCGCATGTACTTGTTCCTCAATAGTTCAGGACTTTTGCTTCTTCCCCAGCCTGGACTCTGACACCTCACCCTTCCTGAATTCCACTCTGTGCAAGTATCTTTCTGTAACACCCTCAGCATCCTGCACTGCCCTCTCTGAAAACA

Amino acids

MKAHILVRVLVMVGFTVGKGKWGPGAGREEGVIEFRKGVERGHGYSGLSFAELRRMYLFLNSSGLLLLPQPGL*

2.-MamLy6g5b-837 HE864467

Nucleotides

CGTCTCCCCAGAATTCCATAATGAAGGCCCATATACTTGTACGTGTGCTGGTCATGGTGGGCTTCACAGTGGGAAAGGGTAAGTGGGGCCCAGGGGCAGGGAGGGAGGAAGGGGTAATTGAGTTCAGGAAGGGGGTGGAACGTGGCCATGGATACTCGGGCTTCCTACTGGCCCAGGGTATCTGAGAGTGACCCAGTGCCCCATCCCTCCTTCTGCCTTCCCAGTTCCTGTTCCCGACATCCGGACCTGCCACTTCTGCCTCTTAGAAGACCCTTCTGTAGGATGCATTTCAGGCTCAGAGAAGTGTACCATCAGCAGCTCATCCCCATGCATGGTGATCACCATCTATTATGATGTCAAGGTTCGCTTCGTCATTCGAGGCTGTGGACAATACTATTCCTACCACTGCCAAGAAAAACGCAACACCTACTTCGTAGAGTACTGGTATCAGGCCCAGTGCTGCCAGTACGATTATTGCAACTCCTGGTCAAGCCCCCAACTCCAGAGCTTCCTGCCGGAGCCCCATGACAGGCCCCTGGCCCTGCCTCTGTCTGACTCCCAGATTCGGTGGTTCTACCAGGCCCTGAACCTCTCACTACCCGTCCCCAATTTCCATGCTGGGAAGGAGCCTGATGGCCTGGACCCCGTGGTCACATTGCCCCTGAACCTGGGCTTGTCTTTTGCTGAGCTGCGCCGCATGTACTTGTTCCTCAATAGTTCAGGACTTTTGCTTCTTCCCCAGCCTGGACTCTGACACCTCACCCTTCCTGAATTCCACTCTGTGCAAGTATCTTTCTGTAACACCCTCAGCATCCTGCACTGCCCTCTCTGAAAACA

Amino acids

MKAHILVRVLVMVGFTVGKGKWGPGAGREEGVIEFRKGVERGHGYSGFLLAQGI*

1.-SsLy6g5b-219 HE864477

Nucleotides

ATGGTGAAGGCTCACGTGCTCGTAGGTATGCTGTTTGTGGTGGGCTTTGCAGAGGGAAAGGCTCCTGTTCCTGAAGTCCGGACCTGCCATCTCTGCCTCTTAGAAGACCCTTTGGTAGGATGCATCTCAGGCTCGGAGAAGTGCACTGTCAGCAGCTCGTCCCCCTGCATGGTGATCACCATCTATTATGGTTGGGCCCTAACATCTCATCCTTCCCGG

Amino acids

MVKAHVLVGMLFVVGFAEGKAPVPEVRTCHLCLLEDPLVGCISGSEKCTVSSSSPCMVITIYYGWALTSHPSR

2.-SsLy6g5b-325 HE864478

Nucleotides

ATGGTGAAGGCTCACGTGCTCGTAGGTATGCTGTTTGTGGTGGGCTTTGCAGAGGGAAAGGGTAAGTGGGGCCCAGGGGCAGGGAGGGAGGGCTGTGGCTCTGGATGGAGGCCAGGGAGCTTGGAGTGGGCCGACATCCCCATCGCTTCTGTCTTCTGCTTCCCCAGCTCCTGTTCCTGAAGTCCGGACCTGCCATCTCTGCCTCTTAGAAGACCCTTTGGTAGGATGCATCTCAGGCTCGGAGAAGTGCACTGTCAGCAGCTCGTCCCCCTGCATGGTGATCACCATCTATTATGGTTGGGCCCTAACATCTCATCCTTCCCGG

Amino acids

MVKAHVLVGMLFVVGFAEGKGKWGPGAGREGCGSGWRPGSLEWADIPIASVFCFPSSCS*

3.-SsLy6g5b-714 HE864479

Nucleotides

ATGGTGAAGGCTCACGTGCTCGTAGGTATGCTGTTTGTGGTGGGCTTTGCAGAGGGAAAGGGTAAGTGGGGCCCAGGGGCAGGGAGGGAGGGCTGTGGCTCTGGATGGAGGCCAGGGAGCTTGGAGTGGGCCGACATCCCCATCGCTTCTGTCTTCTGCTTCCCCAGCTCCTGTTCCTGAAGTCCGGACCTGCCATCTCTGCCTCTTAGAAGACCCTTTGGTAGGATGCATCTCAGGCTCGGAGAAGTGCACTGTCAGCAGCTCGTCCCCCTGCATGGTGATCACCATCTATTATGATGTCAGGGTTCGCTTCTTCATCCGAGGCTGTGGACAGTACAATTCTTTCCGCTGCCAAGAAAAACGCAGCACCTACGTCCCAGAGTACTGGTACCAGGCTGAATGCTGCCAGTACGATTACTGCAACGCCTGGGCCAGCCCGCAGCTCCAGAGCGCCCTCCCTGGGTTCTCTGATGAGTCCCTGGCCCTGCCCCTCTCTGCGTCCCAGATCCAGTGGTTCTACCAAGCCCTGAACCTCTCACTGCCCCTCCCCAGCTTCCATGCTGGGAAGGCACCCGAAGGCCCAGCCCCCCAGGCTGCCCTGCCCCTGAACCTGAGCTTGCCCATTGCTGAGCTGCGTCGCATATACTTGTTCCTCAATCGTTCAGGACTTCTGGTTCTTCCCCAGGTTGGGCCCTAACATCTCATCCTTCCCGG

Amino acids

MVKAHVLVGMLFVVGFAEGKGKWGPGAGREGCGSGWRPGSLEWADIPIASVFCFPSSCS*

1.-BtLy6g5b-178 HE864488

Nucleotides

CTCTCCCCAGAAGTCCATGATGAAGGCCCACATGCTTATAGGTGCACTGGCTGGCCCACCCATGAATCTGAGTTTGTCCATTGCTGACCTGCGCAGCATATACTTGTTTCTCAACAGTTCTGGACTTTTGACTCTTCCTTGGGCTGGTCCCTGACATCTTTCCCGTCGCAGATAGATT

Amino acids

MMKAHMLIGALAGPPMNLSLSIADLRSIYLFLNSSGLLTLPWAGP*

2.-BtLy6g5b-284 HE864489

Nucleotides

CTCTCCCCAGAAGTCCATGATGAAGGCCCACATGCTTATAGGTGCTGCCCCTGTCTCAGTCCCAGATCCTGTGGTTCTACCAAACCCTGAACCTGTCACTGCCCCTGCCCAGCTTCCCTGCTGGGAAGGAGCCTTCTGAAGGCCTGGACCCCCTGGCTGGCCCACCCATGAATCTGAGTTTGTCCATTGCTGACCTGCGCAGCATATACTTGTTTCTCAACAGTTCTGGACTTTTGACTCTTCCTTGGGCTGGTCCCTGACATCTTTCCCGTCGCAGATAGATT

Amino acids

MMKAHMLIGAAPVSVPDPVVLPNPEPVTAPAQLPCWEGAF*

3.-BtLy6g5b-766 HE864490

Nucleotides

CTCTCCCCAGAAGTCCATGATGAAGGCCCACATGCTTATAGGTGCACTGGTCATGGTGGGCTTCACGGTGGGAAAGGGTAAGTGGGGCAGGGGCAGGGAAGGAGGGGTGGCTGAGTACAGGGAGAGGGTGGCGGTGGGTGAGCGGGATGTTGCAGGGTAGGGAACTTGGAGTGGGCCAACACCACCATCCATGTCTTCTGCTTCCCCAGCTCCTGTTTCTGAAGTCCGGACCTGCCACCTCTGCCTCTTAGAAAATCCTGCTGTAGGATGCATTTCAGGCTCGGAGAAGTGCACTATTAGCAGCTCATCCCCATGCATGGTGATCAGCATCAATTATGAAAACAAAGCTCGCTTCTTAATCCGAGGCTGTGGACAACACAATTCCTACCGCTGCCAAGAAAAGCTCCCCACCTACATCTCAGAGTACTGGTACTCGGCCCAGTGCTGCCAGTATGACTACTGCAACTCCTGGTACAGTCCCCAGCTCCAGAGTGCCTCGCCTGAGCCCCTTGACAGGTCCCTGGCTCTGCCCCTGTCTCAGTCCCAGATCCTGTGGTTCTACCAAACCCTGAACCTGTCACTGCCCCTGCCCAGCTTCCCTGCTGGGAAGGAGCCTTCTGAAGGCCTGGACCCCCTGGCTGGCCCACCCATGAATCTGAGTTTGTCCATTGCTGACCTGCGCAGCATATACTTGTTTCTCAACAGTTCTGGACTTTTGACTCTTCCTTGGGCTGGTCCCTGACATCTTTCCCGTCGCAGATAGATT

Amino acids

MMKAHMLIGALVMVGFTVGKGKWGRGREGGVAEYRERVAVGERDVAG*

1.-RnLy6g5b-283 HE864427

Nucleotides

CTACTCCACGGGAGTTGCTCCTCTCCCCTGAAATTCCATAATGAGGGCCTGTGTGCTTGTCCATGTGCTGACCATGGTGGGCTTTGCCTTGGGGAAGGGTAAGTAAGGCCAGGGTCTGGAAAGAGTGGGGAGAGCTGGTCCTGGGTGTGCTTGAAAGACCTCCTGGTTCCGTCTCCTTTGTTCTCTGCGCAAAATATACTTGTTCCTCAACAGTTCAGGACTTCTGGTTCTTCCCCAGGCTAGACCCTGACATGTCCCATCCCCCTTCCCAGATTCTGCTCTG

Amino acids

MRACVLVHVLTMVGFALGKGK*

2.-RnLy6g5b-529 HE864428

Nucleotides

CTACTCCACGGGAGTTGCTCCTCTCCCCTGAAATTCCATAATGAGGGCCTGTGTGCTTGTCCATGTGCTGACCATGGTGGGCTTTGCCTTGGGGAAGGATGTTAAGGTTCGCTTCCACGTACGGGGCTGTGGACAGCACCACTCCTTCCGGTGTCAAGAAAATCACGTCATCTACTACTCAGACTACTGGTATAGGGTTAATTGCTGCCAGTATGATTACTGCAACTCCTGGTCCAGTGCCCAGCACCAGAGCACTCTGCCTGGGCCCCCAGGAAACCATCTGGGTGTGCCCCTCTCTGAGTCTCAGATAAAACAGTTCTACCAGGCCCTGCACCTCCCTCTGTTTCAGCCTGACCTCCACACTCATAAGGTGTCTGAGGGCCCGGACTCTCTCATTCTGCCCCCGGGGCTGGGCTTGTCCATTGCCGACCTGCGCAAAATATACTTGTTCCTCAACAGTTCAGGACTTCTGGTTCTTCCCCAGGCTAGACCCTGACATGTCCCATCCCCCTTCCCAGATTCTGCTCTG

Amino acids

MRACVLVHVLTMVGFALGKDVKVRFHVRGCGQHHSFRCQENHVIYYSDYWYRVNCCQYDYCNSWSSAQHQSTLPGPPGNHLGVPLSESQIKQFYQALHLPLFQPDLHTHKVSEGPDSLILPPGLGLSIADLRKIYLFLNSSGLLVLPQARP*

3.-RnLy6g5b-658 HE864429

Nucleotides

CTACTCCACGGGAGTTGCTCCTCTCCCCTGAAATTCCATAATGAGGGCCTGTGTGCTTGTCCATGTGCTGACCATGGTGGGCTTTGCCTTGGGGAAGGCTCCGGTCGCCAGCGTCCGTACCTGCCACCTGTGCTTCTTAGAAGACCCTTCGATAGGCTGCATTTCTGGGTCAGAAAAGTGCACCATCAGCTCTTCCTCGCCATGCATGGTGATCACCATCTATCAGAATGTTAAGGTTCGCTTCCACGTACGGGGCTGTGGACAGCACCACTCCTTCCGGTGTCAAGAAAATCACGTCATCTACTACTCAGACTACTGGTATAGGGTTAATTGCTGCCAGTATGATTACTGCAACTCCTGGTCCAGTGCCCAGCACCAGAGCACTCTGCCTGGGCCCCCAGGAAACCATCTGGGTGTGCCCCTCTCTGAGTCTCAGATAAAACAGTTCTACCAGGCCCTGCACCTCCCTCTGTTTCAGCCTGACCTCCACACTCATAAGGTGTCTGAGGGCCCGGACTCTCTCATTCTGCCCCCGGGGCTGGGCTTGTCCATTGCCGACCTGCGCAAAATATACTTGTTCCTCAACAGTTCAGGACTTCTGGTTCTTCCCCAGGCTAGACCCTGACATGTCCCATCCCCCTTCCCAGATTCTGCTCTG

Amino acids

MRACVLVHVLTMVGFALGKAPVASVRTCHLCFLEDPSIGCISGSEKCTISSSSPCMVITIYQNVKVRFHVRGCGQHHSFRCQENHVIYYSDYWYRVNCCQYDYCNSWSSAQHQSTLPGPPGNHLGVPLSESQIKQFYQALHLPLFQPDLHTHKVSEGPDSLILPPGLGLSIADLRKIYLFLNSSGLLVLPQARP*

4.-RnLy6g5b-754 HE864430

Nucleotides

CTACTCCACGGGAGTTGCTCCTCTCCCCTGAAATTCCATAATGAGGGCCTGTGTGCTTGTCCATGTGCTGACCATGGTGGGCTTTGCCTTGGGGAAGGGTAAGTAAGGCCAGGGTCTGGAAAGAGTGGGGAGAGCTGGTCCTGGGTGTGCTTGAAAGACCTCCTGGTTCCGTCTCCTTTGTTCTCTGCGCCAAGCTCCGGTCGCCAGCGTCCGTACCTGCCACCTGTGCTTCTTAGAAGACCCTTCGATAGGCTGCATTTCTGGGTCAGAAAAGTGCACCATCAGCTCTTCCTCGCCATGCATGGTGATCACCATCTATCAGAATGTTAAGGTTCGCTTCCACGTACGGGGCTGTGGACAGCACCACTCCTTCCGGTGTCAAGAAAATCACGTCATCTACTACTCAGACTACTGGTATAGGGTTAATTGCTGCCAGTATGATTACTGCAACTCCTGGTCCAGTGCCCAGCACCAGAGCACTCTGCCTGGGCCCCCAGGAAACCATCTGGGTGTGCCCCTCTCTGAGTCTCAGATAAAACAGTTCTACCAGGCCCTGCACCTCCCTCTGTTTCAGCCTGACCTCCACACTCATAAGGTGTCTGAGGGCCCGGACTCTCTCATTCTGCCCCCGGGGCTGGGCTTGTCCATTGCCGACCTGCGCAAAATATACTTGTTCCTCAACAGTTCAGGACTTCTGGTTCTTCCCCAGGCTAGACCCTGACATGTCCCATCCCCCTTCCCAGATTCTGCTCTG

Amino acids

MRACVLVHVLTMVGFALGKGK*

1.- MumLy6g5b-611 HE864438

Nucleotides

TCCCCCAAATTCCATAATGAGGGCCCGCGTGCTTGTAGGTATGCTGACCATGGTGGGCTTTGCGATGGGGAAGGCTCCAGTTGCCAGAGTCCGTACCTGCCACCTGTGCCTCTTAGAAGACCCTTCGCTAGGCTGCATTTCTGGCTCAGAAAAGTGCACCATCAGCCTTCCATCGCCGTGTATGGTGATCACCATCTATAAAAATACTACTGTTCGCTTCCACGTACGGGGCTGTGGACAGCATCATTCCTACCGATGTCAAGAAAGGCATGTGATCTACCAATCAGACTACTTGTATAAGGCTGATTGCTGCCAGTACGATTACTGCAACTCCTGGTCCAGTGCTCAGCACCAGAGCACCCTGCGTGGGTCCCCAGGAAGCCATCTGGGCATGCCCCTGTCAGCGTCTCAGATAAAACAGTTTTACCAGGCCCTGAACCTCTCTCTGCCTCAGCCTGGCTTCCATGCTCATAAGGTGTCTGAGGGCCTGGAGTCTCTCATTCTGCCCCCGGAGCTGGGCTTGTCCATTGCCGACCTTCGCCAAATATACTTGTTCCTCAACAGTTCAGGACTTCTGGTCCTTCCCTGGGATAGACCCTGATGTGTTCCCC

Amino acids

MRARVLVGMLTMVGFAMGKAPVARVRTCHLCLLEDPSLGCISGSEKCTISLPSPCMVITIYKNTTVRFHVRGCGQHHSYRCQERHVIYQSDYLYKADCCQYDYCNSWSSAQHQSTLRGSPGSHLGMPLSASQIKQFYQALNLSLPQPGFHAHKVSEGLESLILPPELGLSIADLRQIYLFLNSSGLLVLPWDRP*

2.- MumLy6g5b-707 HE864439

Nucleotides

TCCCCCAAATTCCATAATGAGGGCCCGCGTGCTTGTAGGTATGCTGACCATGGTGGGCTTTGCGATGGGGAAGGGTAAGTAAGACCAGGGTCTGGGAGGAGTGGGGACAGCTGGTCCTGGGTGTGCTTGAAAGAGCTCCTGGCTCCATCTCCTTTATCCTCTGTGCCGAGCTCCAGTTGCCAGAGTCCGTACCTGCCACCTGTGCCTCTTAGAAGACCCTTCGCTAGGCTGCATTTCTGGCTCAGAAAAGTGCACCATCAGCCTTCCATCGCCGTGTATGGTGATCACCATCTATAAAAATACTACTGTTCGCTTCCACGTACGGGGCTGTGGACAGCATCATTCCTACCGATGTCAAGAAAGGCATGTGATCTACCAATCAGACTACTTGTATAAGGCTGATTGCTGCCAGTACGATTACTGCAACTCCTGGTCCAGTGCTCAGCACCAGAGCACCCTGCGTGGGTCCCCAGGAAGCCATCTGGGCATGCCCCTGTCAGCGTCTCAGATAAAACAGTTTTACCAGGCCCTGAACCTCTCTCTGCCTCAGCCTGGCTTCCATGCTCATAAGGTGTCTGAGGGCCTGGAGTCTCTCATTCTGCCCCCGGAGCTGGGCTTGTCCATTGCCGACCTTCGCCAAATATACTTGTTCCTCAACAGTTCAGGACTTCTGGTCCTTCCCTGGGATAGACCCTGATGTGTTCCCC

Amino acids

MRARVLVGMLTMVGFAMGKGK*

Chimera Csnk2b-Ly6g5b

1.-Chimera HsCSNK2B-LY6G5B-182 HE864452

Nucleotides

GCTGACGTGAAGATGAGCAGCTCAGAGGAGGTGTCCTGGATTTCCTGGTTCTGTGGGCTCCGTGGCAATGAATTCTTCTGTGAAGTGAGTTCTCTTCAACCTCCCTACTTGCCAGCTTCACATATCTTCCCACCAGACGTTCCTTCACATATTCCACTTCTACACTGCCCTCTCTGAAAACA

Amino acids

MSSSEEVSWISWFCGLRGNEFFCEVSSLQPPYLPASHIFPPDVPSHIPLLHCPL*

2.-Chimera HsCSNK2B-LY6G5B-532 HE864453

Nucleotides

GCTGACGTGAAGATGAGCAGCTCAGAGGAGGTGTCCTGGATTTCCTGGTTCTGTGGGCTCCGTGGCAATGAATTCTTCTGTGAAGTGGATGAAGACTACATCCAGGACAAATTTAATCTTACTGGACTCAATGAGCAGGTCCCTCACTACCGACAAGCTCTAGACATGATCTTGGACCTGGAGCCTGATGAAGAACTGGAAGACAACCCCAACCAGAGTGACCTGATTGAGCAGGCAGCCGAGATGCTTTATGGATTGATCCACGCCCGCTACATCCTTACCAACCGTGGCATCGCCCAGATGGTGAGGCCTCTCTGCTCCTACCTGCCTCCTTCTGAGCAGTAAGAGACACAGGTTCCTGCAGCAAGAAGTCATGTTTAAGCCCTGTTTAAGGAAGCTAGCTGAGAAGAGGGGAAGAACCCCAGAACTTGGGCCTGGGAATTGAATTCTGATTGGGGGTCATCCTGAATTCCACAGTGCAAATATCTTTCTGTAACACCCTCAGCATCCTGCACTGCCCTCTCTGAAAACA

Amino acids

MSSSEEVSWISWFCGLRGNEFFCEVDEDYIQDKFNLTGLNEQVPHYRQALDMILDLEPDEELEDNPNQSDLIEQAAEMLYGLIHARYILTNRGIAQMVRPLCSYLPPSEQ*

3.-Chimera HsCSNK2B-LY6G5B-560 HE864454

Nucleotides

GCTGACGTGAAGATGAGCAGCTCAGAGGAGGTGTCCTGGATTTCCTGGTTCTGTGGGCTCCGTGGCAATGAATTCTTCTGTGAAGTGGATGAAGACTACATCCAGGACAAATTTAATCTTACTGGACTCAATGAGCAGGTCCCTCACTACCGACAAGCTCTAGACATGATCTTGGACCTGGAGCCTGATGAAGAACTGGAAGACAACCCCAACCAGAGTGACCTGATTGAGCAGGCAGCCGAGATGCTTTATGGATTGATCCACGCCCGCTACATCCTTACCAACCGTGGCATCGCCCAGATGGTGAGGCCTCTCTGCTCCTACCTGCCTCCTTCTGAGCAGTAAGAGACACAGGTTCCTGCAGCAAGAAGTCATGTTTAAGCCCTGTTTAAGGAAGCTAGCTGAGAAGAGGGGAAGAACCCCAGAACTTGGGCCTGGGAATTGAATTCTGATTGGGGGTCACCAGGCTGGACTCTTGACACCTCACCCTTCCTGAATTCCACAGTGCAAATATCTTTCTGTAACACCCTCAGCATCCTGCACTGCCCTCTCTGAAAACA

Amino acids

MSSSEEVSWISWFCGLRGNEFFCEVDEDYIQDKFNLTGLNEQVPHYRQALDMILDLEPDEELEDNPNQSDLIEQAAEMLYGLIHARYILTNRGIAQMVRPLCSYLPPSEQ*

4.-Chimera HsCSNK2B-LY6G5B-562 HE864455

Nucleotides

GCTGACGTGAAGATGAGCAGCTCAGAGGAGGTGTCCTGGATTTCCTGGTTCTGTGGGCTCCGTGGCAATGAATTCTTCTGTGAAGTGGATGAAGACTACATCCAGGACAAATTTAATCTTACTGGACTCAATGAGCAGGTCCCTCACTACCGACAAGCTCTAGACATGATCTTGGACCTGGAGCCTGATGAAGAACTGGAAGACAACCCCAACCAGAGTGACCTGATTGAGCAGGCAGCCGAGATGCTTTATGGATTGATCCACGCCCTGCCTCTGTCTGACTCCCAGATTCAGTGGTTCTACCAGGCCCTGAACCTCTCCCTGCCCCTCCCCAATTTCCATGCTGGGACGGAGCCTGATGGCCTGGACCCCATGGTCACACTGTCCCTGAACCTGGGCTTGTCTTTTGCTGAGCTGCGCCGCATGTACTTGTTCCTCAATAGTTCAGGACTTTTGGTTCTTCCCCAGGCTGGACTCTTGACACCTCACCCTTCCTGAATTCCACAGTGCAAATATCTTTCTGTAACACCCTCAGCATCCTGCACTGCCCTCTCTGAAAACA

Amino acids

MSSSEEVSWISWFCGLRGNEFFCEVDEDYIQDKFNLTGLNEQVPHYRQALDMILDLEPDEELEDNPNQSDLIEQAAEMLYGLIHALPLSDSQIQWFYQALNLSLPLPNFHAGTEPDGLDPMVTLSLNLGLSFAELRRMYLFLNSSGLLVLPQAGLLTPHPS*

5.-Chimera HsCSNK2B-LY6G5B-696 HE864456

Nucleotides

GCTGACGTGAAGATGAGCAGCTCAGAGGAGGTGTCCTGGATTTCCTGGTTCTGTGGGCTCCGTGGCAATGAATTCTTCTGTGAATTCCTGTTCCCGACATCCGGACGTGCCACTTCTGCCTCGTAGAAGACCCTTCTGTAGGATGCATTTCAGGCTCAGAGAAGTGTACCATCAGCAGCTCATCCCTGTGCATGGTGATCACCATCTATTATGATGTCAAGGTTCGCTTCATCGTTCGAGGCTGTGGACAGTACATTTCCTACCGCTGCCAAGAAAAACGCAACACCTACTTTGCAGAGTACTGGTATCAGGCCCAGTGCTGTCAGTACGATTATTGCAACTCCTGGTCAAGCCCCCAACTCCAGAGCTCTCTGCCGGAGCCCCATGACAGGCCCCTGGCCCTGCCTCTGTCTGACTCCCAGATTCAGTGGTTCTACCAGGCCCTGAACCTCTCCCTGCCCCTCCCCAATTTCCATGCTGGGACGGAGCCTGATGGCCTGGACCCCATGGTCACACTGTCCCTGAACCTGGGCTTGTCTTTTGCTGAGCTGCGCCGCATGTACTTGTTCCTCAATAGTTCAGGACTTTTGGTTCTTCCCCAGGCTGGACTCTTGACACCTCACCCTTCCTGAATTCCACAGTGCAAATATCTTTCTGTAACACCCTCAGCATCCTGCACTGCCCTCTCTGAAAACA

Amino acids

MSSSEEVSWISWFCGLRGNEFFCEFLFPTSGRATSAS*

6.-Chimera HsCSNK2B-LY6G5B-991 HE864457

Nucleotides

GCTGACGTGAAGATGAGCAGCTCAGAGGAGGTGTCCTGGATTTCCTGGTTCTGTGGGCTCCGTGGCAATGAATTCTTCTGTGAAGTGGATGAAGACTACATCCAGGACAAATTTAATCTTACTGGACTCAATGAGCAGGTCCCTCACTACCGACAAGCTCTAGACATGATCTTGGACCTGGAGCCTGATGAAGAACTGGAAGACAACCCCAACCAGAGTGACCTGATTGAGCAGGCAGCCGAGATGCTTTATGGATTGATCCACGCCCGCTACATCCTTACCAACCGTGGCATCGCCCAGATGTTGGAAAAGTACCAGCAAGGAGACTTTGGTTACTGTCCTCGTGTGTACTGTGAGAACCAGCCAATGCTTCCCATTGTTCCTGTTCCCGACATCCGGACGTGCCACTTCTGCCTCGTAGAAGACCCTTCTGTAGGATGCATTTCAGGCTCAGAGAAGTGTACCATCAGCAGCTCATCCCTGTGCATGGTGATCACCATCTATTATGATGTCAAGGTTCGCTTCATCGTTCGAGGCTGTGGACAGTACATTTCCTACCGCTGCCAAGAAAAACGCAACACCTACTTTGCAGAGTACTGGTATCAGGCCCAGTGCTGTCAGTACGATTATTGCAACTCCTGGTCAAGCCCCCAACTCCAGAGCTCTCTGCCGGAGCCCCATGACAGGCCCCTGGCCCTGCCTCTGTCTGACTCCCAGATTCAGTGGTTCTACCAGGCCCTGAACCTCTCCCTGCCCCTCCCCAATTTCCATGCTGGGACGGAGCCTGATGGCCTGGACCCCATGGTCACACTGTCCCTGAACCTGGGCTTGTCTTTTGCTGAGCTGCGCCGCATGTACTTGTTCCTCAATAGTTCAGGACTTTTGGTTCTTCCCCAGGCTGGACTCTTGACACCTCACCCTTCCTGAATTCCACAGTGCAAATATCTTTCTGTAACACCCTCAGCATCCTGCACTGCCCTCTCTGAAAACA

Amino acids

MSSSEEVSWISWFCGLRGNEFFCEVDEDYIQDKFNLTGLNEQVPHYRQALDMILDLEPDEELEDNPNQSDLIEQAAEMLYGLIHARYILTNRGIAQMLEKYQQGDFGYCPRVYCENQPMLPIVPVPDIRTCHFCLVEDPSVGCISGSEKCTISSSSLCMVITIYYDVKVRFIVRGCGQYISYRCQEKRNTYFAEYWYQAQCCQYDYCNSWSSPQLQSSLPEPHDRPLALPLSDSQIQWFYQALNLSLPLPNFHAGTEPDGLDPMVTLSLNLGLSFAELRRMYLFLNSSGLLVLPQAGLLTPHPS*

7.-Chimera HsCSNK2B-LY6G5B-1072 HE864458

Nucleotides

GCTGACGTGAAGATGAGCAGCTCAGAGGAGGTGTCCTGGATTTCCTGGTTCTGTGGGCTCCGTGGCAATGAATTCTTCTGTGAAGTGGATGAAGACTACATCCAGGACAAATTTAATCTTACTGGACTCAATGAGCAGGTCCCTCACTACCGACAAGCTCTAGACATGATCTTGGACCTGGAGCCTGGTGAGGCACCCTCAGGGTTGTTTTGTGTGTGTGCGTGCACTATTTTTCTCTTCAAATCTCTATTCACTTGCCTGAATTTTGAAATTTCCTTTGGTTCTCTGATTTCTTTAACCCCAAATTCATGCTTTATTTTGATCCTCCACCTGACTCTTGTCTAGTTTTGTGACGTATATCACTTGTTCTCATGTTTTCTAAATCCGCAATTCAGACCTCATGCGTTTCCTCATGGGTCTGGTTTGTTGTCTGTTTCTCCTGCTTTGCACCTTCCAGTCTAGAGTTTCATCTTCTGCATTGACATTGTTGCAGTTATGTATTGAGGAGGGAGTTGGGAGGGAGAGCAAGGAGCAGAGGCTGAAAAGGTGTGAAGGGAAGGCAGAGCTGTCTTCGTTTGATGCAAGGGTCAGAAGCCCAGGTTTCTGGGTCCCATGCCCAGATGTTGGATGGGGTAAGGCCCAAAAGTAGGTGCTAGGCAAACTGAATAGCCCGCAGCCCCTGGATATGGGCAGGGCACCTAGGAAAGCTGAAAAACAAGTAGTTGCATTTGGCCGGGCTGTGTTTCAGATGAAGAACTGGAAGACAACCCCAACCAGAGTGACCTGATTGAGCAGGCAGCCGAGATGCTTTATGGATTGATCCACGCCCGCTACATCCTTACCAACCGTGGCATCGCCCAGATGGTGAGGCCTCTCTGCTCCTACCTGCCTCCTTCTGAGCAGTAAGAGACACAGGTTCCTGCAGCAAGAAGTCATGTTTAAGCCCTGTTTAAGGAAGCTAGCTGAGAAGAGGGGAAGAACCCCAGAACTTGGGCCTGGGAATTGAATTCTGATTGGGGGTCATCCTGAAAACACCCACATTCTTTGGTCACTGTGATTTCTTAGGCCTCCG

Amino acids

MSSSEEVSWISWFCGLRGNEFFCEVDEDYIQDKFNLTGLNEQVPHYRQALDMILDLEPGEAPSGLFCVCACTIFLFKSLFTCLNFEISFGSLISLTPNSCFILILHLTLV*

8.-Chimera HsCSNK2B-LY6G5B-1103 HE864459

Nucleotides

GCTGACGTGAAGATGAGCAGCTCAGAGGAGGTGTCCTGGATTTCCTGGTTCTGTGGGCTCCGTGGCAATGAATTCTTCTGTGAAGTGGATGAAGACTACATCCAGGACAAATTTAATCTTACTGGACTCAATGAGCAGGTCCCTCACTACCGACAAGCTCTAGACATGATCTTGGACCTGGAGCCTGGTGAGGCACCCTCAGGGTTGTTTTGTGTGTGTGCGTGCACTATTTTTCTCTTCAAATCTCTATTCACTTGCCTGAATTTTGAAATTTCCTTTGGTTCTCTGATTTCTTTAACCCCAAATTCATGCTTTATTTTGATCCTCCACCTGACTCTTGTCTAGTTTTGTGACGTATATCACTTGTTCTCATGTTTTCTAAATCCGCAATTCAGACCTCTTCCAAAATGCGTTTCCTCATGGGTCTGGTTTGTTGTCTGTTTCTCCTGCTTTGCACCTTCCAGTCTAGAGTTTCATCTTCTGCATTGACATTGTTGCAGTTATGTATTGAGGAGGGAGTTGGGAGGGAGAGCAAGGAGCAGAGGCTGAAAAGGTGTGAAGGGAAGGCAGAGCTGTCTTCGTTTGATGCAAGGGTCAGAAGCCCAGGTTTCTGGGTCCCATGCCCAGATGTTGGATGGGGTAAGGCCCAAAAGTAGGTGCTAGGCAAACTGAATAGCCCGCAGCCCCTGGATATGGGCAGGGCACCTAGGAAAGCTGAAAAACAAGTAGTTGCATTTGGCCGGGCTGTGTTTCAGATGAAGAACTGGAAGACAACCCCAACCAGAGTGACCTGATTGAGCAGGCAGCCGAGATGCTTTATGGATTGATCCACGCCCGCTACATCCTTACCAACCGTGGCATCGCCCAGATGGTGAGGCCTCTCTGCTCCTACCTGCCTCCTTCTGAGCAGTAAGAGACACAGGTTCCTGCAGCAAGAAGTCATGTTTAAGCCCTGTTTAAGGAAGCTAGCTGAGAAGAGGGGAAGAACCCCAGAACTTGGGCCTGGGAATTGAATTCTGATTGGGGGTCATCTTCCTGAATTCCACAGTGCAAATATCTTTCTGTAACACCCTCAGCATCCTGCACTGCCCTCTCTGAAAACA

Amino acids

MSSSEEVSWISWFCGLRGNEFFCEVDEDYIQDKFNLTGLNEQVPHYRQALDMILDLEPGEAPSGLFCVCACTIFLFKSLFTCLNFEISFGSLISLTPNSCFILILHLTLV*

9.-Chimera HsCSNK2B-LY6G5B-1181 HE864460

Nucleotides

GCTGACGTGAAGATGAGCAGCTCAGAGGAGGTGTCCTGGATTTCCTGGTTCTGTGGGCTCCGTGGCAATGAATTCTTCTGTGAAGTGGATGAAGACTACATCCAGGACAAATTTAATCTTACTGGACTCAATGAGCAGGTCCCTCACTACCGACAAGCTCTAGACATGATCTTGGACCTGGAGCCTGATGAAGAACTGGAAGACAACCCCAACCAGAGTGACCTGATTGAGCAGGCAGCCGAGATGCTTTATGGATTGATCCACGCCCGCTACATCCTTACCAACCGTGGCATCGCCCAGATGTTGGAAAAGTACCAGCAAGGAGACTTTGGTTACTGTCCTCGTGTGTACTGTGAGAACCAGCCAATGCTTCCCATTGGCCTTTCAGACATCCCAGGTGAAGCCATGGTGAAGCTCTACTGCCCCAAGTGCATGGATGTGTACACACCCAAGTCATCAAGACACCATCACACGGATGGCGCCTACTTCGGCACTGGTTTCCCTCACATGCTCTTCATGGTGCATCCCGAGTACCGGCCCAAGAGACCTGCCAACCAGTTTGTGCCCAGTTCCTGTTCCCGACATCCGGACGTGCCACTTCTGCCTCGTAGAAGACCCTTCTGTAGGATGCATTTCAGGCTCAGAGAAGTGTACCATCAGCAGCTCATCCCTGTGCATGGTGATCACCATCTATTATGATGTCAAGGTTCGCTTCATCGTTCGAGGCTGTGGACAGTACATTTCCTACCGCTGCCAAGAAAAACGCAACACCTACTTTGCAGAGTACTGGTATCAGGCCCAGTGCTGTCAGTACGATTATTGCAACTCCTGGTCAAGCCCCCAACTCCAGAGCTCTCTGCCGGAGCCCCATGACAGGCCCCTGGCCCTGCCTCTGTCTGACTCCCAGATTCAGTGGTTCTACCAGGCCCTGAACCTCTCCCTGCCCCTCCCCAATTTCCATGCTGGGACGGAGCCTGATGGCCTGGACCCCATGGTCACACTGTCCCTGAACCTGGGCTTGTCTTTTGCTGAGCTGCGCCGCATGTACTTGTTCCTCAATAGTTCAGGACTTTTGGTTCTTCCCCAGGCTGGACTCTTGACACCTCACCCTTCCTGAATTCCACAGTGCAAATATCTTTCTGTAACACCCTCAGCATCCTGCACTGCCCTCTCTGAAAACA

Amino acids

MSSSEEVSWISWFCGLRGNEFFCEVDEDYIQDKFNLTGLNEQVPHYRQALDMILDLEPDEELEDNPNQSDLIEQAAEMLYGLIHARYILTNRGIAQMLEKYQQGDFGYCPRVYCENQPMLPIGLSDIPGEAMVKLYCPKCMDVYTPKSSRHHHTDGAYFGTGFPHMLFMVHPEYRPKRPANQFVPSSCSRHPDVPLLPRRRPFCRMHFRLREVYHQQLIPVHGDHHLL*

10.-Chimera HsCSNK2B-LY6G5B-1327 HE864461

Nucleotides

GCTGACGTGAAGATGAGCAGCTCAGAGGAGGTGTCCTGGATTTCCTGGTTCTGTGGGCTCCGTGGCAATGAATTCTTCTGTGAAGTGGATGAAGACTACATCCAGGACAAATTTAATCTTACTGGACTCAATGAGCAGGTCCCTCACTACCGACAAGCTCTAGACATGATCTTGGACCTGGAGCCTGATGAAGAACTGGAAGACAACCCCAACCAGAGTGACCTGATTGAGCAGGCAGCCGAGATGCTTTATGGATTGATCCACGCCCGCTACATCCTTACCAACCGTGGCATCGCCCAGATGTTGGAAAAGTACCAGCAAGGAGACTTTGGTTACTGTCCTCGTGTGTACTGTGAGAACCAGCCAATGCTTCCCATTGGTGAGTGTTGAAGAAGGGAAAGGAAAGCACCGTGTGGCAGTCTTATGGGAAGGAGTTGGGGCTCAACACATTGGAGCCTGAGTCCTGAGGGGAGGTTAGGTAGGAATAGGGGGATACCTGGCCTGCTGAGTCTGGCTGTCTCCCAGGCCTTTCAGACATCCCAGGTGAAGCCATGGTGAAGCTCTACTGCCCCAAGTGCATGGATGTGTACACACCCAAGTCATCAAGACACCATCACACGGATGGCGCCTACTTCGGCACTGGTTTCCCTCACATGCTCTTCATGGTGCATCCCGAGTACCGGCCCAAGAGACCTGCCAACCAGTTTGTGCCCAGTTCCTGTTCCCGACATCCGGACGTGCCACTTCTGCCTCGTAGAAGACCCTTCTGTAGGATGCATTTCAGGCTCAGAGAAGTGTACCATCAGCAGCTCATCCCTGTGCATGGTGATCACCATCTATTATGATGTCAAGGTTCGCTTCATCGTTCGAGGCTGTGGACAGTACATTTCCTACCGCTGCCAAGAAAAACGCAACACCTACTTTGCAGAGTACTGGTATCAGGCCCAGTGCTGTCAGTACGATTATTGCAACTCCTGGTCAAGCCCCCAACTCCAGAGCTCTCTGCCGGAGCCCCATGACAGGCCCCTGGCCCTGCCTCTGTCTGACTCCCAGATTCAGTGGTTCTACCAGGCCCTGAACCTCTCCCTGCCCCTCCCCAATTTCCATGCTGGGACGGAGCCTGATGGCCTGGACCCCATGGTCACACTGTCCCTGAACCTGGGCTTGTCTTTTGCTGAGCTGCGCCGCATGTACTTGTTCCTCAATAGTTCAGGACTTTTGGTTCTTCCCCAGGCTGGACTCTTGACACCTCACCCTTCCTGAATTCCACAGTGCAAATATCTTTCTGTAACACCCTCAGCATCCTGCACTGCCCTCTCTGAAAACA

Amino acids

MSSSEEVSWISWFCGLRGNEFFCEVDEDYIQDKFNLTGLNEQVPHYRQALDMILDLEPDEELEDNPNQSDLIEQAAEMLYGLIHARYILTNRGIAQMLEKYQQGDFGYCPRVYCENQPMLPIGEC*

1.-Chimera MamCsnk2b-Ly6g5b-992 HE864468

Nucleotides

GCTGACGTGAAGATGAGCAGCTCAGAGGAGGTGTCCTGGATTTCCTGGTTCTGTGGGCTCCGTGGCAATGAATTCTTCTGTGAAGTGGATGAAGACTACATCCAGGACAAATTTAATCTTACTGGACTCAATGAGCAGGTTCCTCACTATCGACAAGCTCTAGACATGATCTTGGACCTGGAGCCTGATGAAGAACTGGAAGACAACCCCAACCAGAGTGACCTGATTGAGCAGGCTGCCGAGATGCTTTATGGATTGATCCACGCCCGCTACATCCTTACCAACCGTGGCATTGCCCAGATGTTGGAAAAGTACCAGCAGGGAGACTTTGGTTACTGTCCTCGTGTGTACTGTGAGAACCAGCCAATGCTTCCCATCGTTCCTGTTCCCGACATCCGGACCTGCCACTTCTGCCTCTTAGAAGACCCTTCTGTAGGATGCATTTCAGGCTCAGAGAAGTGTACCATCAGCAGCTCATCCCCATGCATGGTGATCACCATCTATTATGATGTCAAGGTTCGCTTCGTCATTCGAGGCTGTGGACAATACTATTCCTACCACTGCCAAGAAAAACGCAACACCTACTTCGTAGAGTACTGGTATCAGGCCCAGTGCTGCCAGTACGATTATTGCAACTCCTGGTCAAGCCCCCAACTCCAGAGCTTCCTGCCGGAGCCCCATGACAGGCCCCTGGCCCTGCCTCTGTCTGACTCCCAGATTCGGTGGTTCTACCAGGCCCTGAACCTCTCACTACCCGTCCCCAATTTCCATGCTGGGAAGGAGCCTGATGGCCTGGACCCCGTGGTCACATTGCCCCTGAACCTGGGCTTGTCTTTTGCTGAGCTGCGCCGCATGTACTTGTTCCTCAATAGTTCAGGACTTTTGCTTCTTCCCCAGCCTGGACTCTGACACCTCACCCTTCCTGAATTCCACTCTGTGCAAGTATCTTTCTGTAACACCCTCAGCATCCTGCACTGCCCTCTCTGAAAACA

Amino acids

MSSSEEVSWISWFCGLRGNEFFCEVDEDYIQDKFNLTGLNEQVPHYRQALDMILDLEPDEELEDNPNQSDLIEQAAEMLYGLIHARYILTNRGIAQMLEKYQQGDFGYCPRVYCENQPMLPIVPVPDIRTCHFCLLEDPSVGCISGSEKCTISSSSPCMVITIYYDVKVRFVIRGCGQYYSYHCQEKRNTYFVEYWYQAQCCQYDYCNSWSSPQLQSFLPEPHDRPLALPLSDSQIRWFYQALNLSLPVPNFHAGKEPDGLDPVVTLPLNLGLSFAELRRMYLFLNSSGLLLLPQPGL*

2.-Chimera MamCsnk2b-Ly6g5b-1141 HE864469

Nucleotides

GCTGACGTGAAGATGAGCAGCTCAGAGGAGGTGTCCTGGATTTCCTGGTTCTGTGGGCTCCGTGGCAATGAATTCTTCTGTGAAGTGGATGAAGACTACATCCAGGACAAATTTAATCTTACTGGACTCAATGAGCAGGTTCCTCACTATCGACAAGCTCTAGACATGATCTTGGACCTGGAGCCTGATGAAGAACTGGAAGACAACCCCAACCAGAGTGACCTGATTGAGCAGGCTGCCGAGATGCTTTATGGATTGATCCACGCCCGCTACATCCTTACCAACCGTGGCATTGCCCAGATGTTGGAAAAGTACCAGCAGGGAGACTTTGGTTACTGTCCTCGTGTGTACTGTGAGAACCAGCCAATGCTTCCCATCGGCTCTACGGTTTCAAGATTCATCCGATGGCCTACCAGCTGCAGCTCCAAGCCGCCAGCAACTTCAAGAGCCCAGTCAAGACGATTCGCTGATTCCCTACCCCACCTGTCCTGCACTTCCTTTCCTTTTTGCCACCCTTTCAGGAACCCTTTCCTGTTCCCGACATCCGGACCTGCCACTTCTGCCTCTTAGAAGACCCTTCTGTAGGATGCATTTCAGGCTCAGAGAAGTGTACCATCAGCAGCTCATCCCCATGCATGGTGATCACCATCTATTATGATGTCAAGGTTCGCTTCGTCATTCGAGGCTGTGGACAATACTATTCCTACCACTGCCAAGAAAAACGCAACACCTACTTCGTAGAGTACTGGTATCAGGCCCAGTGCTGCCAGTACGATTATTGCAACTCCTGGTCAAGCCCCCAACTCCAGAGCTTCCTGCCGGAGCCCCATGACAGGCCCCTGGCCCTGCCTCTGTCTGACTCCCAGATTCGGTGGTTCTACCAGGCCCTGAACCTCTCACTACCCGTCCCCAATTTCCATGCTGGGAAGGAGCCTGATGGCCTGGACCCCGTGGTCACATTGCCCCTGAACCTGGGCTTGTCTTTTGCTGAGCTGCGCCGCATGTACTTGTTCCTCAATAGTTCAGGACTTTTGCTTCTTCCCCAGCCTGGACTCTGACACCTCACCCTTCCTGAATTCCACTCTGTGCAAGTATCTTTCTGTAACACCCTCAGCATCCTGCACTGCCCTCTCTGAAAACA

Amino acids

MSSSEEVSWISWFCGLRGNEFFCEVDEDYIQDKFNLTGLNEQVPHYRQALDMILDLEPDEELEDNPNQSDLIEQAAEMLYGLIHARYILTNRGIAQMLEKYQQGDFGYCPRVYCENQPMLPIGSTVSRFIRWPTSCSSKPPATSRAQSRRFADSLPHLSCTSFPFCHPFRNPFLFPTSGPATSAS*

3.-Chimera MamCsnk2b-Ly6g5b-1218 HE864470

Nucleotides

GCTGACGTGAAGATGAGCAGCTCAGAGGAGGTGTCCTGGATTTCCTGGTTCTGTGGGCTCCGTGGCAATGAATTCTTCTGTGAAGTGGATGAAGACTACATCCAGGACAAATTTAATCTTACTGGACTCAATGAGCAGGTTCCTCACTATCGACAAGCTCTAGACATGATCTTGGACCTGGAGCCTGATGAAGAACTGGAAGACAACCCCAACCAGAGTGACCTGATTGAGCAGGCTGCCGAGATGCTTTATGGATTGATCCACGCCCGCTACATCCTTACCAACCGTGGCATTGCCCAGATGTTGGAAAAGTACCAGCAGGGAGACTTTGGTTACTGTCCTCGTGTGTACTGTGAGAACCAGCCAATGCTTCCCATCGGCCTTTCAGACATCCCAGGTGAAGCCATGGTGAAGCTCTACTGCCCCAAGTGCATGGATGTGTACACACCCAAGTCATCGAGACACCATCACACGGATGGCGCCTACTTTGGCACTGGTTTCCCTCACATGCTCTTCATGGTGCATCCCGAGTACCGGCCCAAGAGACCCGCCAACCAGTTTGTGCCCAGGTGTGCTGGTCATGGTGGGCTTCACAGTGGGAAAGGTTCCTGTTCCCGACATCCGGACCTGCCACTTCTGCCTCTTAGAAGACCCTTCTGTAGGATGCATTTCAGGCTCAGAGAAGTGTACCATCAGCAGCTCATCCCCATGCATGGTGATCACCATCTATTATGATGTCAAGGTTCGCTTCGTCATTCGAGGCTGTGGACAATACTATTCCTACCACTGCCAAGAAAAACGCAACACCTACTTCGTAGAGTACTGGTATCAGGCCCAGTGCTGCCAGTACGATTATTGCAACTCCTGGTCAAGCCCCCAACTCCAGAGCTTCCTGCCGGAGCCCCATGACAGGCCCCTGGCCCTGCCTCTGTCTGACTCCCAGATTCGGTGGTTCTACCAGGCCCTGAACCTCTCACTACCCGTCCCCAATTTCCATGCTGGGAAGGAGCCTGATGGCCTGGACCCCGTGGTCACATTGCCCCTGAACCTGGGCTTGTCTTTTGCTGAGCTGCGCCGCATGTACTTGTTCCTCAATAGTTCAGGACTTTTGCTTCTTCCCCAGCCTGGACTCTGACACCTCACCCTTCCTGAATTCCACTCTGTGCAAGTATCTTTCTGTAACACCCTCAGCATCCTGCACTGCCCTCTCTGAAAACA

Amino acids

MSSSEEVSWISWFCGLRGNEFFCEVDEDYIQDKFNLTGLNEQVPHYRQALDMILDLEPDEELEDNPNQSDLIEQAAEMLYGLIHARYILTNRGIAQMLEKYQQGDFGYCPRVYCENQPMLPIGLSDIPGEAMVKLYCPKCMDVYTPKSSRHHHTDGAYFGTGFPHMLFMVHPEYRPKRPANQFVPRCAGHGGLHSGKGSCSRHPDLPLLPLRRPFCRMHFRLREVYHQQLIPMHGDHHLL*

4.-Chimera MamCsnk2b-Ly6g5b-1331 HE864471

Nucleotides

GCTGACGTGAAGATGAGCAGCTCAGAGGAGGTGTCCTGGATTTCCTGGTTCTGTGGGCTCCGTGGCAATGAATTCTTCTGTGAAGTGGATGAAGACTACATCCAGGACAAATTTAATCTTACTGGACTCAATGAGCAGGTTCCTCACTATCGACAAGCTCTAGACATGATCTTGGACCTGGAGCCTGATGAAGAACTGGAAGACAACCCCAACCAGAGTGACCTGATTGAGCAGGCTGCCGAGATGCTTTATGGATTGATCCACGCCCGCTACATCCTTACCAACCGTGGCATTGCCCAGATGTTGGAAAAGTACCAGCAGGGAGACTTTGGTTACTGTCCTCGTGTGTACTGTGAGAACCAGCCAATGCTTCCCATCGGCCTTTCAGACATCCCAGGTGAAGCCATGGTGAAGCTCTACTGCCCCAAGTGCATGGATGTGTACACACCCAAGTCATCGAGACACCATCACACGGATGGCGCCTACTTTGGCACTGGTTTCCCTCACATGCTCTTCATGGTGCATCCCGAGTACCGGCCCAAGAGACCCGCCAACCAGTTTGTGCCCAGGCTCTACGGTTTCAAGATTCATCCGATGGCCTACCAGCTGCAGCTCCAAGCCGCCAGCAACTTCAAGAGCCCAGTCAAGACGATTCGCTGATTCCCTACCCCACCTGTCCTGCACTTCCTTTCCTTTTTGCCACCCTTTCAGGAACCCTTTCCTGTTCCCGACATCCGGACCTGCCACTTCTGCCTCTTAGAAGACCCTTCTGTAGGATGCATTTCAGGCTCAGAGAAGTGTACCATCAGCAGCTCATCCCCATGCATGGTGATCACCATCTATTATGATGTCAAGGTTCGCTTCGTCATTCGAGGCTGTGGACAATACTATTCCTACCACTGCCAAGAAAAACGCAACACCTACTTCGTAGAGTACTGGTATCAGGCCCAGTGCTGCCAGTACGATTATTGCAACTCCTGGTCAAGCCCCCAACTCCAGAGCTTCCTGCCGGAGCCCCATGACAGGCCCCTGGCCCTGCCTCTGTCTGACTCCCAGATTCGGTGGTTCTACCAGGCCCTGAACCTCTCACTACCCGTCCCCAATTTCCATGCTGGGAAGGAGCCTGATGGCCTGGACCCCGTGGTCACATTGCCCCTGAACCTGGGCTTGTCTTTTGCTGAGCTGCGCCGCATGTACTTGTTCCTCAATAGTTCAGGACTTTTGCTTCTTCCCCAGCCTGGACTCTGACACCTCACCCTTCCTGAATTCCACTCTGTGCAAGTATCTTTCTGTAACACCCTCAGCATCCTGCACTGCCCTCTCTGAAAACA

Amino acids

MSSSEEVSWISWFCGLRGNEFFCEVDEDYIQDKFNLTGLNEQVPHYRQALDMILDLEPDEELEDNPNQSDLIEQAAEMLYGLIHARYILTNRGIAQMLEKYQQGDFGYCPRVYCENQPMLPIGLSDIPGEAMVKLYCPKCMDVYTPKSSRHHHTDGAYFGTGFPHMLFMVHPEYRPKRPANQFVPRLYGFKIHPMAYQLQLQAASNFKSPVKTIR*

5.- Chimera MamCsnk2b-Ly6g5b-2338 HE864472

Nucleotides

GCTGACGTGAAGATGAGCAGCTCAGAGGAGGTGTCCTGGATTTCCTGGTTCTGTGGGCTCCGTGGCAATGAATTCTTCTGTGAAGTGGATGAAGACTACATCCAGGACAAATTTAATCTTACTGGACTCAATGAGCAGGTTCCTCACTATCGACAAGCTCTAGACATGATCTTGGACCTGGAGCCTGATGAAGAACTGGAAGACAACCCCAACCAGAGTGACCTGATTGAGCAGGCTGCCGAGATGCTTTATGGATTGATCCACGCCCGCTACATCCTTACCAACCGTGGCATTGCCCAGATGTTGGAAAAGTACCAGCAGGGAGACTTTGGTTACTGTCCTCGTGTGTACTGTGAGAACCAGCCAATGCTTCCCATCGGCCTTTCAGACATCCCAGGTGAAGCCATGGTGAAGCTCTACTGCCCCAAGTGCATGGATGTGTACACACCCAAGTCATCGAGACACCATCACACGGATGGCGCCTACTTTGGCACTGGTTTCCCTCACATGCTCTTCATGGTGCATCCCGAGTACCGGCCCAAGAGACCCGCCAACCAGTTTGTGCCCAGGCTCTACGGTTTCAAGATTCATCCGATGGCCTACCAGCTGCAGCTCCAAGCCGCCAGCAACTTCAAGAGCCCAGTCAAGACGATTCGCTGATTCCCTACCCCACCTGTCCTGCACTTCCTTTCCTTTTTGCCACCCTTTCAGGAACCCTGTATGGTTTTTAGTTTAAATTAAAGGAGTCATTATTGTGGTGGGAATATGAAATAAAGTGGAAGAAAAGGCCATGAGCTAGTCTGCTGGTGCTTGCGGTTGGGGAAGGGAAGGTGATGGTGTGTTGGACTCCAGGGGCCCTCATGGCCCAGCCCACCCTCCCCAGATTGAAAACCAGGACAGATTTGTGCTCAGTGAATTGGGTGATGTTTTTAGTATAGAGCAGAACAGAATTCCTAGGACTATGTGTGATGAAGTGCAAGGTCAAAAGGAAAAGACAGAAAAAGCATGTTTTAAAGATGAGAAGTATTTGGATACCTATGACTGTCTGTCTATACTGTAAGGTGCTTAATCAGCAACTCCATCTTTCAGTTTTAAAGGAAAAGTAGCCCTAAAGTCAGTATAACTAAGGGTGGAATGAGGTGGGACAAGGTCCAGAATTACTGCTCAGTGATGTGTGTGTGTGCTGCCTGCTGGTGGAGCTGGGACTGCCCATCTCAGAAGGATGGGGATGCTTGATTTCCAGGCCAGGTTGTCCCAGCACAGTGGGGACTGGCCCTGTTATATGAGGAAGACAGCACATGGTGGCAGAGATAGACACTAAGCCATGGACTTTCCAAGGGAGGGAGTAGGTCTTTGGAGGGTATGCAGGACAAAGGTAGACACTGGATAAAGAACCAGATAGTGCCCAGATATTACCCCGTCTGGGCCATTGCTCCCACACTCAGGAACCAGACGTTGTGGGTGAGGACATGCTGTCCCTCCTGCCAAGTTAATAACTTCCTTCCCAACCAGGATCCTGCCCCAAGCAGGAATATAGCTCTGCATTTACAGCAGCTCCTCAGACCTTGTCAAAACCACCCTGCAACTCAGGATTAAGGAGCGTGGTCACAGGAAGGTGGGGTTTCAGGGCATCCCTCAGGAACTGCCCGTCTCCCCAGAATTCCATAATGAAGGCCCATATACTTGTACGTGTGCTGGTCATGGTGGGCTTCACAGTGGGAAAGGTTCCTGTTCCCGACATCCGGACCTGCCACTTCTGCCTCTTAGAAGACCCTTCTGTAGGATGCATTTCAGGCTCAGAGAAGTGTACCATCAGCAGCTCATCCCCATGCATGGTGATCACCATCTATTATGATGTCAAGGTTCGCTTCGTCATTCGAGGCTGTGGACAATACTATTCCTACCACTGCCAAGAAAAACGCAACACCTACTTCGTAGAGTACTGGTATCAGGCCCAGTGCTGCCAGTACGATTATTGCAACTCCTGGTCAAGCCCCCAACTCCAGAGCTTCCTGCCGGAGCCCCATGACAGGCCCCTGGCCCTGCCTCTGTCTGACTCCCAGATTCGGTGGTTCTACCAGGCCCTGAACCTCTCACTACCCGTCCCCAATTTCCATGCTGGGAAGGAGCCTGATGGCCTGGACCCCGTGGTCACATTGCCCCTGAACCTGGGCTTGTCTTTTGCTGAGCTGCGCCGCATGTACTTGTTCCTCAATAGTTCAGGACTTTTGCTTCTTCCCCAGCCTGGACTCTGACACCTCACCCTTCCTGAATTCCACTCTGTGCAAGTATCTTTCTGTAACACCCTCAGCATCCTGCACTGCCCTCTCTGAAAACA

Amino acids

MSSSEEVSWISWFCGLRGNEFFCEVDEDYIQDKFNLTGLNEQVPHYRQALDMILDLEPDEELEDNPNQSDLIEQAAEMLYGLIHARYILTNRGIAQMLEKYQQGDFGYCPRVYCENQPMLPIGLSDIPGEAMVKLYCPKCMDVYTPKSSRHHHTDGAYFGTGFPHMLFMVHPEYRPKRPANQFVPRLYGFKIHPMAYQLQLQAASNFKSPVKTIR*

1.-Chimera SsCsnk2b-Ly6g5b-538 HE864480

Nucleotides

CGCTGAAGTGAAGATGAGCAGCTCAGAGGAGGTGTCCTGGATTTCCTGGTTCTGTGGGCTCCGTGGCAATGAATTCTTCTGTGAAGTGGATGAAGACTACATCCAGGACAAATTCAATCTCACTGGACTCAACGAGCAGGTGCCTCATTATCGACAAGCCCTAGACATGATCTTGGACCTGGAACCTGATGAGGAGCTGGAAGACAACCCCAACCAGAGTGACCTGATTGAGCAGGCAGCCGAAATGCTCTATGGATTGATCCACGCCCGCTATATCCTCACCAACCGTGGCATCGCCCAGATGTTGGAAAAGTACCAGCAGGGAGACTTTGGCTACTGTCCCCGTGTGTACTGTGAGAACCAGCCAATGCTTCCCATCGCTCCTGTTCCTGAAGTCCGGACCTGCCATCTCTGCCTCTTAGAAGACCCTTTGGTAGGATGCATCTCAGGCTCGGAGAAGTGCACTGTCAGCAGCTCGTCCCCCTGCATGGTGATCACCATCTATTATGGTTGGGCCCTAACATCTCATCCTTCCCGG

Amino acids

MSSSEEVSWISWFCGLRGNEFFCEVDEDYIQDKFNLTGLNEQVPHYRQALDMILDLEPDEELEDNPNQSDLIEQAAEMLYGLIHARYILTNRGIAQMLEKYQQGDFGYCPRVYCENQPMLPIAPVPEVRTCHLCLLEDPLVGCISGSEKCTVSSSSPCMVITIYYGWALTSHPSR

2.- Chimera SsCsnk2b-Ly6g5b-728 HE864481

Nucleotides

CGCTGAAGTGAAGATGAGCAGCTCAGAGGAGGTGTCCTGGATTTCCTGGTTCTGTGGGCTCCGTGGCAATGAATTCTTCTGTGAAGTGGATGAAGACTACATCCAGGACAAATTCAATCTCACTGGACTCAACGAGCAGGTGCCTCATTATCGACAAGCCCTAGACATGATCTTGGACCTGGAACCTGATGAGGAGCTGGAAGACAACCCCAACCAGAGTGACCTGATTGAGCAGGCAGCCGAAATGCTCTATGGATTGATCCACGCCCGCTATATCCTCACCAACCGTGGCATCGCCCAGATGTTGGAAAAGTACCAGCAGGGAGACTTTGGCTACTGTCCCCGTGTGTACTGTGAGAACCAGCCAATGCTTCCCATCGGCCTTTCGGACATCCCAGGCGAGGCCATGGTGAAGCTCTACTGCCCCAAGTGCATGGACGTGTACACGCCCAAGTCATCGAGGCACCACCACACGGATGGCGCCTACTTCGGCACCGGTTTCCCTCACATGCTCTTCATGGTGCACCCCGAGTACCGGCCCAAACGGCCTGCCAACCAGTTTGTGCCCAGCTCCTGTTCCTGAAGTCCGGACCTGCCATCTCTGCCTCTTAGAAGACCCTTTGGTAGGATGCATCTCAGGCTCGGAGAAGTGCACTGTCAGCAGCTCGTCCCCCTGCATGGTGATCACCATCTATTATGGTTGGGCCCTAACATCTCATCCTTCCCGG

Amino acids

MSSSEEVSWISWFCGLRGNEFFCEVDEDYIQDKFNLTGLNEQVPHYRQALDMILDLEPDEELEDNPNQSDLIEQAAEMLYGLIHARYILTNRGIAQMLEKYQQGDFGYCPRVYCENQPMLPIGLSDIPGEAMVKLYCPKCMDVYTPKSSRHHHTDGAYFGTGFPHMLFMVHPEYRPKRPANQFVPSSCS*

3.-Chimera SsCsnk2b-Ly6g5b-927 HE864482

Nucleotides

CGCTGAAGTGAAGATGAGCAGCTCAGAGGAGGTGTCCTGGATTTCCTGGTTCTGTGGGCTCCGTGGCAATGAATTCTTCTGTGAAGTGGATGAAGACTACATCCAGGACAAATTCAATCTCACTGGACTCAACGAGCAGGTGCCTCATTATCGACAAGCCCTAGACATGATCTTGGACCTGGAACCTGATGAGGAGCTGGAAGACAACCCCAACCAGAGTGACCTGATTGAGCAGGCAGCCGAAATGCTCTATGGATTGATCCACGCCCGCTATATCCTCACCAACCGTGGCATCGCCCAGATGTTGGAAAAGTACCAGCAGGGAGACTTTGGCTACTGTCCCCGTGTGTACTGTGAGAACCAGCCAATGCTTCCCATCGCTCCTGTTCCTGAAGTCCGGACCTGCCATCTCTGCCTCTTAGAAGACCCTTTGGTAGGATGCATCTCAGGCTCGGAGAAGTGCACTGTCAGCAGCTCGTCCCCCTGCATGGTGATCACCATCTATTATGATGTCAGGGTTCGCTTCTTCATCCGAGGCTGTGGACAGTACAATTCTTTCCGCTGCCAAGAAAAACGCAGCACCTACGTCCCAGAGTACTGGTACCAGGCTGAATGCTGCCAGTACGATTACTGCAACGCCTGGGCCAGCCCGCAGCTCCAGAGCGCCCTCCCTGGGTTCTCTGATGAGTCCCTGGCCCTGCCCCTCTCTGCGTCCCAGATCCAGTGGTTCTACCAAGCCCTGAACCTCTCACTGCCCCTCCCCAGCTTCCATGCTGGGAAGGCACCCGAAGGCCCAGCCCCCCAGGCTGCCCTGCCCCTGAACCTGAGCTTGCCCATTGCTGAGCTGCGTCGCATATACTTGTTCCTCAATCGTTCAGGACTTCTGGTTCTTCCCCAGGTTGGGCCCTAACATCTCATCCTTCCCGG

Amino acids

MSSSEEVSWISWFCGLRGNEFFCEVDEDYIQDKFNLTGLNEQVPHYRQALDMILDLEPDEELEDNPNQSDLIEQAAEMLYGLIHARYILTNRGIAQMLEKYQQGDFGYCPRVYCENQPMLPIAPVPEVRTCHLCLLEDPLVGCISGSEKCTVSSSSPCMVITIYYDVRVRFFIRGCGQYNSFRCQEKRSTYVPEYWYQAECCQYDYCNAWASPQLQSALPGFSDESLALPLSASQIQWFYQALNLSLPLPSFHAGKAPEGPAPQAALPLNLSLPIAELRRIYLFLNRSGLLVLPQVGP*

1.- Chimera BtCsnk2b-Ly6g5b-201 HE864415

Nucleotides

CCGACGTGAAGATGAGCAGCTCAGAGGAGGTGTCCTGGATTTCCTGGTTCTGTGGGCTCCGTGGCAATGAATTCTTCTGTGAGGTGGATGAAGACTATATCCATTGCTGACCTGCGCAGCATATACTTGTTTCTCAACAGTTCTGGACTTTTGACTCTTCCTTGGGCTGGTCCCTGACATCtttcccgtcgcagAtagatt

Amino acids

MSSSEEVSWISWFCGLRGNEFFCEVDEDYIHC*

2.- Chimera BtCsnk2b-Ly6g5b-306 HE864416

Nucleotides

CCGACGTGAAGATGAGCAGCTCAGAGGAGGTGTCCTGGATTTCCTGGTTCTGTGGGCTCCGTGGCTCTGCCCCTGTCTCAGTCCCAGATCCTGTGGTTCTACCAAACCCTGAACCTGTCACTGCCCCTGCCCAGCTTCCCTGCTGGGAAGGAGCCTTCTGAAGGCCTGGACCCCCTGGCTGGCCCACCCATGAATCTGAGTTTGTCCATTGCTGACCTGCGCAGCATATACTTGTTTCTCAACAGTTCTGGACTTTTGACTCTTCCTTGGGCTGGTCCCTGACATCtttcccgtcgcagatagatt

Amino acids

MSSSEEVSWISWFCGLRGSAPVSVPDPVVLPNPEPVTAPAQLPCWEGAF*

3.- Chimera BtCsnk2b-Ly6g5b-368 HE864417

Nucleotides

CCGACGTGAAGATGAGCAGCTCAGAGTACTGGTACTCGGCCCAGTGCTGCCAGTATGACTACTGCAACTCCTGGTACAGTCCCCAGCTCCAGAGTGCCTCGCCTGAGCCCCTTGACAGGTCCCTGGCTCTGCCCCTGTCTCAGTCCCAGATCCTGTGGTTCTACCAAACCCTGAACCTGTCACTGCCCCTGCCCAGCTTCCCTGCTGGGAAGGAGCCTTCTGAAGGCCTGGACCCCCTGGCTGGCCCACCCATGAATCTGAGTTTGTCCATTGCTGACCTGCGCAGCATATACTTGTTTCTCAACAGTTCTGGACTTTTGACTCTTCCTTGGGCTGGTCCCTGACATCtttcccgtcgcagatagatt

Amino acids

MSSSEYWYSAQCCQYDYCNSWYSPQLQSASPEPLDRSLALPLSQSQILWFYQTLNLSLPLPSFPAGKEPSEGLDPLAGPPMNLSLSIADLRSIYLFLNSSGLLTLPWAGP*

4.- Chimera BtCsnk2b-Ly6g5b-460 HE864418

Nucleotides

CCGACGTGAAGATGAGCAGCTCAGAGGAGGTGTCCTGGATTTCCTGGTTCTGTGGGCTCCGTGGCAATGAATTCTTCTGTGAGGTGGATGAAGACTATATCCAGGACAAATTCAATCTCACTGGTACTCGGCCCAGTGCTGCCAGTATGACTACTGCAACTCCTGGTACAGTCCCCAGCTCCAGAGTGCCTCGCCTGAGCCCCTTGACAGGTCCCTGGCTCTGCCCCTGTCTCAGTCCCAGATCCTGTGGTTCTACCAAACCCTGAACCTGTCACTGCCCCTGCCCAGCTTCCCTGCTGGGAAGGAGCCTTCTGAAGGCCTGGACCCCCTGGCTGGCCCACCCATGAATCTGAGTTTGTCCATTGCTGACCTGCGCAGCATATACTTGTTTCTCAACAGTTCTGGACTTTTGACTCTTCCTTGGGCTGGTCCCTGACATCtttcccgtcgcagatagatt

Amino acids

MSSSEEVSWISWFCGLRGNEFFCEVDEDYIQDKFNLTGTRPSAASMTTATPGTVPSSRVPRLSPLTGPWLCPCLSPRSCGSTKP*

5.- Chimera BtCsnk2b-Ly6g5b-640 HE864419

Nucleotides

CCGACGTGAAGATGAGCAGCTCAGAGGAGGTGTCCTGGATTTCCTGGTTCTGTGGGCTCCGTGGCAATGAATTCTTCTGTGAGCTCCTGTTTCTGAAGTCCGGACCTGCCACCTCTGCCTCTTAGAAAATCCTGCTGTAGGATGCATTTCAGGCTCGGAGAAGTGCACTATTAGCAGCTCATCCCCATGCATGGTGATCAGCATCAATTATGAAAACAAAGCTCGCTTCTTAATCCGAGGCTGTGGACAACACAATTCCTACCGCTGCCAAGAAAAGCTCCCCACCTACATCTCAGAGTACTGGTACTCGGCCCAGTGCTGCCAGTATGACTACTGCAACTCCTGGTACAGTCCCCAGCTCCAGAGTGCCTCGCCTGAGCCCCTTGACAGGTCCCTGGCTCTGCCCCTGTCTCAGTCCCAGATCCTGTGGTTCTACCAAACCCTGAACCTGTCACTGCCCCTGCCCAGCTTCCCTGCTGGGAAGGAGCCTTCTGAAGGCCTGGACCCCCTGGCTGGCCCACCCATGAATCTGAGTTTGTCCATTGCTGACCTGCGCAGCATATACTTGTTTCTCAACAGTTCTGGACTTTTGACTCTTCCTTGGGCTGGTCCCTGACATCtttcccgtcgcagatagatt

Amino acids

MSSSEEVSWISWFCGLRGNEFFCELLFLKSGPATSAS*

6.- Chimera BtCsnk2b-Ly6g5b- 725 HE864420

Nucleotides

CCGACGTGAAGATGAGCAGCTCAGAGGAGGTGTCCTGGATTTCCTGGTTCTGTGGGCTCCGTGGCAATGAATTCTTCTGTGAGGTGGATGAAGACTATATCCAGGACAAATTCAATCTCACTGGACTCAATGAGCAGGTGCCTCACTATCGACAAGCTTTAGACATGATCTTGGACCTGGAGCCTGATGAGGAGCTGGAGGACAACCCCAACCAGAGTGACCTGATTGAGCAAGCAGGCTCGGAGAAGTGCACTATTAGCAGCTCATCCCCATGCATGGTGATCAGCATCAATTATGAAAACAAAGCTCGCTTCTTAATCCGAGGCTGTGGACAACACAATTCCTACCGCTGCCAAGAAAAGCTCCCCACCTACATCTCAGAGTACTGGTACTCGGCCCAGTGCTGCCAGTATGACTACTGCAACTCCTGGTACAGTCCCCAGCTCCAGAGTGCCTCGCCTGAGCCCCTTGACAGGTCCCTGGCTCTGCCCCTGTCTCAGTCCCAGATCCTGTGGTTCTACCAAACCCTGAACCTGTCACTGCCCCTGCCCAGCTTCCCTGCTGGGAAGGAGCCTTCTGAAGGCCTGGACCCCCTGGCTGGCCCACCCATGAATCTGAGTTTGTCCATTGCTGACCTGCGCAGCATATACTTGTTTCTCAACAGTTCTGGACTTTTGACTCTTCCTTGGGCTGGTCCCTGACATCtttcccgtcgcagatagatt

Amino acids

MSSSEEVSWISWFCGLRGNEFFCEVDEDYIQDKFNLTGLNEQVPHYRQALDMILDLEPDEELEDNPNQSDLIEQAGSEKCTISSSSPCMVISINYENKARFLIRGCGQHNSYRCQEKLPTYISEYWYSAQCCQYDYCNSWYSPQLQSASPEPLDRSLALPLSQSQILWFYQTLNLSLPLPSFPAGKEPSEGLDPLAGPPMNLSLSIADLRSIYLFLNSSGLLTLPWAGP*

7.- Chimera BtCsnk2b-Ly6g5b-737 HE864421

Nucleotides

CCGACGTGAAGATGAGCAGCTCAGAGGAGGTGTCCTGGATTTCCTGGTTCTGTGGGCTCCGTGGCAATGAATTCTTCTGTGAGGTGGATGAAGACTATATCCAGGACAAATTCAATCTCACTGGACTCAATGAGCAGGTGCCTCACTATCGACAAGCTTTAGACATGATCTTGGACCTGGAGCCTGTTTCTGAAGTCCGGACCTGCCACCTCTGCCTCTTAGAAAATCCTGCTGTAGGATGCATTTCAGGCTCGGAGAAGTGCACTATTAGCAGCTCATCCCCATGCATGGTGATCAGCATCAATTATGAAAACAAAGCTCGCTTCTTAATCCGAGGCTGTGGACAACACAATTCCTACCGCTGCCAAGAAAAGCTCCCCACCTACATCTCAGAGTACTGGTACTCGGCCCAGTGCTGCCAGTATGACTACTGCAACTCCTGGTACAGTCCCCAGCTCCAGAGTGCCTCGCCTGAGCCCCTTGACAGGTCCCTGGCTCTGCCCCTGTCTCAGTCCCAGATCCTGTGGTTCTACCAAACCCTGAACCTGTCACTGCCCCTGCCCAGCTTCCCTGCTGGGAAGGAGCCTTCTGAAGGCCTGGACCCCCTGGCTGGCCCACCCATGAATCTGAGTTTGTCCATTGCTGACCTGCGCAGCATATACTTGTTTCTCAACAGTTCTGGACTTTTGACTCTTCCTTGGGCTGGTCCCTGACATCtttcccgtcgcagatagatt

Amino acids

MSSSEEVSWISWFCGLRGNEFFCEVDEDYIQDKFNLTGLNEQVPHYRQALDMILDLEPVSEVRTCHLCLLENPAVGCISGSEKCTISSSSPCMVISINYENKARFLIRGCGQHNSYRCQEKLPTYISEYWYSAQCCQYDYCNSWYSPQLQSASPEPLDRSLALPLSQSQILWFYQTLNLSLPLPSFPAGKEPSEGLDPLAGPPMNLSLSIADLRSIYLFLNSSGLLTLPWAGP*

8.- Chimera BtCsnk2b-Ly6g5b-805 HE864422

Nucleotides

CCGACGTGAAGATGAGCAGCTCAGAGGAGGTGTCCTGGATTTCCTGGTTCTGTGGGCTCCGTGGCAATGAATTCTTCTGTGAGgtaagttctcctcaaccctaccatccacctccactggctagacagcccatggcattgtctcctcttgtaaatgagggctgcttggggcctgaggtcTAAggagatggaggtttctggtaggggaggagtctggggttctggggagccaattaagctaatttcttccagcctcttctgcaggagtcgggaaaggaaattgggtctaggtgagggatgggcaggtctgtggaaggggtaccaggtttggtgactatgggaggcagcaagggttattagttgtcccttcttctccaGAAAACAAAGCTCGCTTCTTAATCCGAGGCTGTGGACAACACAATTCCTACCGCTGCCAAGAAAAGCTCCCCACCTACATCTCAGAGTACTGGTACTCGGCCCAGTGCTGCCAGTATGACTACTGCAACTCCTGGTACAGTCCCCAGCTCCAGAGTGCCTCGCCTGAGCCCCTTGACAGGTCCCTGGCTCTGCCCCTGTCTCAGTCCCAGATCCTGTGGTTCTACCAAACCCTGAACCTGTCACTGCCCCTGCCCAGCTTCCCTGCTGGGAAGGAGCCTTCTGAAGGCCTGGACCCCCTGGCTGGCCCACCCATGAATCTGAGTTTGTCCATTGCTGACCTGCGCAGCATATACTTGTTTCTCAACAGTTCTGGACTTTTGACTCTTCCTTGGGCTGGTCCCTGACATCtttcccgtcgcagatagatt

Amino acids

MSSSEEVSWISWFCGLRGNEFFCEVSSPQPYHPPPLARQPMALSPLVNEGCLGPEV*

9.- Chimera BtCsnk2b-Ly6g5b-1049 HE864423

Nucleotides

CCGACGTGAAGATGAGCAGCTCAGAGGAGGTGTCCTGGATTTCCTGGTTCTGTGGGCTCCGTGGCAATGAATTCTTCTGTGAGGTGGATGAAGACTATATCCAGGACAAATTCAATCTCACTGGACTCAATGAGCAGGTGCCTCACTATCGACAAGCTTTAGACATGATCTTGGACCTGGAGCCTGATGAGGAGCTGGAGGACAACCCCAACCAGAGTGACCTGATTGAGCAAGCAGCTGAAATGCTCTATGGATTGATCCACGCCCGCTATATCCTCACCAACCGTGGCATTGCCCAGATGTTGGAAAAGTACCAGCAGGGAGACTTTGGATACTGTCCCCGTGTGTACTGTGAGAACCAGCCAATGCTTCCCATCGGCTTTACGGTTTCAAGATCCATCCAATGGCCTACCAGCTGCAGCTCCAAGCCGCCAGCAACTTCAAGAGCCCAGTGAAGACGATTCGCTGATTCCCCACCCCACCTGTCCCTCGCTCCTGTTTCTGAAGTCCGGACCTGCCACCTCTGCCTCTTAGAAAATCCTGCTGTAGGATGCATTTCAGGCTCGGAGAAGTGCACTATTAGCAGCTCATCCCCATGCATGGTGATCAGCATCAATTATGAAAACAAAGCTCGCTTCTTAATCCGAGGCTGTGGACAACACAATTCCTACCGCTGCCAAGAAAAGCTCCCCACCTACATCTCAGAGTACTGGTACTCGGCCCAGTGCTGCCAGTATGACTACTGCAACTCCTGGTACAGTCCCCAGCTCCAGAGTGCCTCGCCTGAGCCCCTTGACAGGTCCCTGGCTCTGCCCCTGTCTCAGTCCCAGATCCTGTGGTTCTACCAAACCCTGAACCTGTCACTGCCCCTGCCCAGCTTCCCTGCTGGGAAGGAGCCTTCTGAAGGCCTGGACCCCCTGGCTGGCCCACCCATGAATCTGAGTTTGTCCATTGCTGACCTGCGCAGCATATACTTGTTTCTCAACAGTTCTGGACTTTTGACTCTTCCTTGGGCTGGTCCCTGACATCtttcccgtcgcagatagatt

Amino acids

MSSSEEVSWISWFCGLRGNEFFCEVDEDYIQDKFNLTGLNEQVPHYRQALDMILDLEPDEELEDNPNQSDLIEQAAEMLYGLIHARYILTNRGIAQMLEKYQQGDFGYCPRVYCENQPMLPIGFTVSRSIQWPTSCSSKPPATSRAQ*

10.- Chimera BtCsnk2b-Ly6g5b-1239 HE864424

Nucleotides

CCGACGTGAAGATGAGCAGCTCAGAGGAGGTGTCCTGGATTTCCTGGTTCTGTGGGCTCCGTGGCAATGAATTCTTCTGTGAGGTGGATGAAGACTATATCCAGGACAAATTCAATCTCACTGGACTCAATGAGCAGGTGCCTCACTATCGACAAGCTTTAGACATGATCTTGGACCTGGAGCCTGATGAGGAGCTGGAGGACAACCCCAACCAGAGTGACCTGATTGAGCAAGCAGCTGAAATGCTCTATGGATTGATCCACGCCCGCTATATCCTCACCAACCGTGGCATTGCCCAGATGTTGGAAAAGTACCAGCAGGGAGACTTTGGATACTGTCCCCGTGTGTACTGTGAGAACCAGCCAATGCTTCCCATCGGCCTTTCAGACATCCCAGGTGAGGCCATGGTGAAGCTCTACTGCCCCAAGTGCATGGACGTGTACACACCCAAGTCATCGAGGCACCACCACACGGATGGCGCCTACTTCGGCACCGGTTTCCCTCACATGCTCTTCATGGTGCACCCCGAGTACCGGCCCAAGAGGCCCGCCAACCAGTTTGTGCCCAGGCTTTACGGTTTCAAGATCCATCCAATGGCCTACCAGCTGCAGCTCCAAGCCGCCAGCAACTTCAAGAGCCCAGTGAAGACGATTCGCTGATTCCCCACCCCACCTGTCCCTCGCTCCTGTTTCTGAAGTCCGGACCTGCCACCTCTGCCTCTTAGAAAATCCTGCTGTAGGATGCATTTCAGGCTCGGAGAAGTGCACTATTAGCAGCTCATCCCCATGCATGGTGATCAGCATCAATTATGAAAACAAAGCTCGCTTCTTAATCCGAGGCTGTGGACAACACAATTCCTACCGCTGCCAAGAAAAGCTCCCCACCTACATCTCAGAGTACTGGTACTCGGCCCAGTGCTGCCAGTATGACTACTGCAACTCCTGGTACAGTCCCCAGCTCCAGAGTGCCTCGCCTGAGCCCCTTGACAGGTCCCTGGCTCTGCCCCTGTCTCAGTCCCAGATCCTGTGGTTCTACCAAACCCTGAACCTGTCACTGCCCCTGCCCAGCTTCCCTGCTGGGAAGGAGCCTTCTGAAGGCCTGGACCCCCTGGCTGGCCCACCCATGAATCTGAGTTTGTCCATTGCTGACCTGCGCAGCATATACTTGTTTCTCAACAGTTCTGGACTTTTGACTCTTCCTTGGGCTGGTCCCTGACATCtttcccgtcgcagatagatt

Amino acids

MSSSEEVSWISWFCGLRGNEFFCEVDEDYIQDKFNLTGLNEQVPHYRQALDMILDLEPDEELEDNPNQSDLIEQAAEMLYGLIHARYILTNRGIAQMLEKYQQGDFGYCPRVYCENQPMLPIGLSDIPGEAMVKLYCPKCMDVYTPKSSRHHHTDGAYFGTGFPHMLFMVHPEYRPKRPANQFVPRLYGFKIHPMAYQLQLQAASNFKSPVKTIR*

1.- Chimera RnCsnk2b-Ly6g5b-901 HE864431

Nucleotides

CCGCGGACATAAAGATGAGTAGCTCTGAGGAGGTGTCCTGGATTTCCTGGTTCTGTGGGCTCCGTGGTAATGAATTCTTCTGTGAGGTGGATGAAGACTACATCCAGGACAAATTTAATCTTACTGGACTCAATGAGCAGGTGCCTCACTATCGACAAGCCCTAGACATGATCTTGGACCTGGAACCTGATGAAGAGCTGGAAGACAACCCCAACCAGAGTGACTTGATTGAGCAGGCGGCCGAGATGCTCTATGGGTTGATCCACGCCCGCTACATCCTCACCAACCGGGGCATTGCACAAATGTTGGAAAAGTACCAGCAAGGAGACTTTGGCTACTGTCCTCGAGTATACTGTGAGAACCAGCCGATGCTTCCCATCGGCCTTTCGGACATCCCAGGAGAGGCCATGGTGAAGCTCTACTGCCCCAAGTGCATGGACGTGTACACACCCAAGTCCCCTCTCCTCTAGATGTTAAGGTTCGCTTCCACGTACGGGGCTGTGGACAGCACCACTCCTTCCGGTGTCAAGAAAATCACGTCATCTACTACTCAGACTACTGGTATAGGGTTAATTGCTGCCAGTATGATTACTGCAACTCCTGGTCCAGTGCCCAGCACCAGAGCACTCTGCCTGGGCCCCCAGGAAACCATCTGGGTGTGCCCCTCTCTGAGTCTCAGATAAAACAGTTCTACCAGGCCCTGCACCTCCCTCTGTTTCAGCCTGACCTCCACACTCATAAGGTGTCTGAGGGCCCGGACTCTCTCATTCTGCCCCCGGGGCTGGGCTTGTCCATTGCCGACCTGCGCAAAATATACTTGTTCCTCAACAGTTCAGGACTTCTGGTTCTTCCCCAGGCTAGACCCTGACATGTCCCATCCCCCTTCCCAGATTCTGCTCTG

Amino acids

MSSSEEVSWISWFCGLRGNEFFCEVDEDYIQDKFNLTGLNEQVPHYRQALDMILDLEPDEELEDNPNQSDLIEQAAEMLYGLIHARYILTNRGIAQMLEKYQQGDFGYCPRVYCENQPMLPIGLSDIPGEAMVKLYCPKCMDVYTPKSPLL*

2.- Chimera RnCsnk2b-Ly6g5b-2050 HE864432

Nucleotides

ccgcggacataaagATGagtagctctgaggaggtgtcctggatttcctggttctgtgggctccgtggtaatgaattcttctgtgaggtggatgaagactacatccaggacaaatttaatcttactggactcaatgagcaggtgcctcactatcgacaagccctagacatgatcttggacctggaacctgatgaagagctggaagacaaccccaaccagagtgacttgattgagcaggcggccgagatgctctatgggttgatccacgcccgctacatcctcaccaaccggggcattgcacaaatgttggaaaagtaccagcaaggagactttggctactgtcctcgagtatactgtgagaaccagccgatgcttcccatcggcctttcggacatcccaggagaggccatggtgaagctctactgccccaagtgcatggacgtgtacacacccaagtcctctaggcaccaccacacggatggcgcatacttcggcactggtttccctcacatgctcttcatggtgcatcccgagtaccggcccaagcggccggccaaccagtttgtgcccaggctctacggtttcaagatccatccaatggcctaccagctgcagctccaagccgccagcaacttcaagagcccagtcaagacgattcgcTGAgtgccctcccacctcctctgcctgtgacaccaccgtccctccgctgccaccctttcaggaagtctatggtttttagtttaaattaaaggaattgttactgtggtggggatatgaaataaaggaagaaaaggcTATGAGCTTGTCTCCTGCTGCGTGCTGCTAGGGAAGGGCTGAGGAACGGAAGGGGTGGTTGCTGGACTCCAGGGACCGCAAGGTCTAGTCTGTCCTCCCTAGGTTGGGTCGGGTCAAGTACCAGACTGAGAAGAAAGGTTTCAAAGGCTGGAGAAGACCTGCTCCCTTTGGGCTCTGGCTGTGTCTGCTGTGGGGGCTTCATGGGAAGTTCCCTCAGCTGTGCTGCTAAAGGGGCTCTACCCCTCAGCAAATAGAGCTGTAGAGGAGGCAAAGAGTCCCAGTTGCTCAGTGGTGTCTGTGTGCCCCCTGCTGGGAGAGCCGAGACACAGAGACACATCTGGAAAGGTTGGGGAGGCTTGTCTCTGGCCAACCTCTCCCCGAATAATGGGGACCAACCCTGCTGTGTAGGTGGACAGCAGGCAGTGCCAGAGACAGACTAAGCCGTGGTCCCTGAGGTGGGGGTATGGGCCTTTCAAGGCAGTGTCTGGGATGAAGACACCTGATAAAGCCAGCTGGTACTCGGCTGTGGCCCACATGGGAAACCAGACAGTGTGGGTGAGGACGGGCCATTCTTCCTGCCAATAGATAACTTCCTCCTCTGGTCAGGACCCTGCCTCAGACAGGAGTGCACCTCTGCACCCACAGCAGTTTCTGCTCAGACTGCCTGTCTAACCCATTCTGCCTCTGAAGATCAAGGAGTGTGATCCCAGGAAGGTGGGGCTGTGGGCTACTCCACGGGAGTTGCTCCTCTCCCCTGAAATTCCATAATGagggcctgtgtgcttgtccatgtgctgaccatggtgggctttgccttggggaaggGtgttaaggttcgcttccacgtacggggctgtggacagcaccactccttccggtgtcaagaaaatcacgtcatctactactcagactactggtatagggttaattgctgccagtatgattactgcaactcctggtccagtgcccagcaccagagcactctgcctgggcccccaggaaaccatctgggtgtgcccctctctgagtctcagataaaacagttctaccaggccctgcacctccctctgtttcagcctgacctccacactcataaggtgtctgagggcccggactctctcattctgcccccggggctgggcttgtccattgccgacctgcgcaaaatatacttgttcctcaacagttcaggacttctggttcttccccaggctagacccTGAcatgtcccatcccccttcccagattctgctctg

Amino acids

MSSSEEVSWISWFCGLRGNEFFCEVDEDYIQDKFNLTGLNEQVPHYRQALDMILDLEPDEELEDNPNQSDLIEQAAEMLYGLIHARYILTNRGIAQMLEKYQQGDFGYCPRVYCENQPMLPIGLSDIPGEAMVKLYCPKCMDVYTPKSSRHHHTDGAYFGTGFPHMLFMVHPEYRPKRPANQFVPRLYGFKIHPMAYQLQLQAASNFKSPVKTIR*

3.- Chimera RnCsnk2b-Ly6g5b-2275 HE864433

Nucleotides

ccgcggacataaagATGagtagctctgaggaggtgtcctggatttcctggttctgtgggctccgtggtaatgaattcttctgtgaggtggatgaagactacatccaggacaaatttaatcttactggactcaatgagcaggtgcctcactatcgacaagccctagacatgatcttggacctggaacctgatgaagagctggaagacaaccccaaccagagtgacttgattgagcaggcggccgagatgctctatgggttgatccacgcccgctacatcctcaccaaccggggcattgcacaaatgttggaaaagtaccagcaaggagactttggctactgtcctcgagtatactgtgagaaccagccgatgcttcccatcggcctttcggacatcccaggagaggccatggtgaagctctactgccccaagtgcatggacgtgtacacacccaagtcctctaggcaccaccacacggatggcgcatacttcggcactggtttccctcacatgctcttcatggtgcatcccgagtaccggcccaagcggccggccaaccagtttgtgcccaggctctacggtttcaagatccatccaatggcctaccagctgcagctccaagccgccagcaacttcaagagcccagtcaagacgattcgcTGAgtgccctcccacctcctctgcctgtgacaccaccgtccctccgctgccaccctttcaggaagtctatggtttttagtttaaattaaaggaattgttactgtggtggggatatgaaataaaggaagaaaaggcTATGAGCTTGTCTCCTGCTGCGTGCTGCTAGGGAAGGGCTGAGGAACGGAAGGGGTGGTTGCTGGACTCCAGGGACCGCAAGGTCTAGTCTGTCCTCCCTAGGTTGGGTCGGGTCAAGTACCAGACTGAGAAGAAAGGTTTCAAAGGCTGGAGAAGACCTGCTCCCTTTGGGCTCTGGCTGTGTCTGCTGTGGGGGCTTCATGGGAAGTTCCCTCAGCTGTGCTGCTAAAGGGGCTCTACCCCTCAGCAAATAGAGCTGTAGAGGAGGCAAAGAGTCCCAGTTGCTCAGTGGTGTCTGTGTGCCCCCTGCTGGGAGAGCCGAGACACAGAGACACATCTGGAAAGGTTGGGGAGGCTTGTCTCTGGCCAACCTCTCCCCGAATAATGGGGACCAACCCTGCTGTGTAGGTGGACAGCAGGCAGTGCCAGAGACAGACTAAGCCGTGGTCCCTGAGGTGGGGGTATGGGCCTTTCAAGGCAGTGTCTGGGATGAAGACACCTGATAAAGCCAGCTGGTACTCGGCTGTGGCCCACATGGGAAACCAGACAGTGTGGGTGAGGACGGGCCATTCTTCCTGCCAATAGATAACTTCCTCCTCTGGTCAGGACCCTGCCTCAGACAGGAGTGCACCTCTGCACCCACAGCAGTTTCTGCTCAGACTGCCTGTCTAACCCATTCTGCCTCTGAAGATCAAGGAGTGTGATCCCAGGAAGGTGGGGCTGTGGGCTACTCCACGGGAGTTGCTCCTCTCCCCTGAAATTCCATAatgagggcctgtgtgcttgtccatgtgctgaccatggtgggctttgccttggggaaggGTAAGTAAGGCCAGGGTCTGGAAAGAGTGGGGAGAGCTGGTCCTGGGTGTGCTTGAAAGACCTCCTGGTTCCGTCTCCTTTGTTCTCTGCGCCAAGctccggtcgccagcgtccgtacctgccacctgtgcttcttagaagacccttcgataggctgcatttctgggtcagaaaagtgcaccatcagctcttcctcgccATGcatggtgatcaccatctatcagaatgttaaggttcgcttccacgtacggggctgtggacagcaccactccttccggtgtcaagaaaatcacgtcatctactactcagactactggtatagggttaattgctgccagtatgattactgcaactcctggtccagtgcccagcaccagagcactctgcctgggcccccaggaaaccatctgggtgtgcccctctctgagtctcagataaaacagttctaccaggccctgcacctccctctgtttcagcctgacctccacactcataaggtgtctgagggcccggactctctcattctgcccccggggctgggcttgtccattgccgacctgcgcaaaatatacttgttcctcaacagttcaggacttctggttcttccccaggctagacccTGAcatgtcccatcccccttcccagattctgctctg

Amino acids

MSSSEEVSWISWFCGLRGNEFFCEVDEDYIQDKFNLTGLNEQVPHYRQALDMILDLEPDEELEDNPNQSDLIEQAAEMLYGLIHARYILTNRGIAQMLEKYQQGDFGYCPRVYCENQPMLPIGLSDIPGEAMVKLYCPKCMDVYTPKSSRHHHTDGAYFGTGFPHMLFMVHPEYRPKRPANQFVPRLYGFKIHPMAYQLQLQAASNFKSPVKTIR*

4.- Chimera RnCsnk2b-Ly6g5b-2531 HE864434

Nucleotides

ccgcggacataaagATGagtagctctgaggaggtgtcctggatttcctggttctgtgggctccgtggtaatgaattcttctgtgaggtggatgaagactacatccaggacaaatttaatcttactggactcaatgagcaggtgcctcactatcgacaagccctagacatgatcttggacctggaacctgatgaagagctggaagacaaccccaaccagagtgacttgattgagcaggcggccgagatgctctatgggttgatccacgcccgctacatcctcaccaaccggggcattgcacaaatgttggaaaagtaccagcaaggagactttggctactgtcctcgagtatactgtgagaaccagccgatgcttcccatcggcctttcggacatcccaggagaggccatggtgaagctctactgccccaagtgcatggacgtgtacacacccaagtcctctaggcaccaccacacggatggcgcatacttcggcactggtttccctcacatgctcttcatggtgcatcccgagtaccggcccaagcggccggccaaccagtttgtgcccaggctctacggtttcaagatccatccaatggcctaccagctgcagctccaagccgccagcaacttcaagagcccagtcaagacgattcgcTGAgtgccctcccacctcctctgcctgtgacaccaccgtccctccgctgccaccctttcaggaagtctatggtttttagtttaaattaaaggaattgttactgtggtggggatatgaaataaaggaagaaaaggcTATGAGCTTGTCTCCTGCTGCGTGCTGCTAGGGAAGGGCTGAGGAACGGAAGGGGTGGTTGCTGGACTCCAGGGACCGCAAGGTCTAGTCTGTCCTCCCTAGGTTGGGTCGGGTCAAGTACCAGACTGAGAAGAAAGGTTTCAAAGGCTGGAGAAGACCTGCTCCCTTTGGGCTCTGGCTGTGTCTGCTGTGGGGGCTTCATGGGAAGTTCCCTCAGCTGTGCTGCTAAAGGGGCTCTACCCCTCAGCAAATAGAGCTGTAGAGGAGGCAAAGAGTCCCAGTTGCTCAGTGGTGTCTGTGTGCCCCCTGCTGGGAGAGCCGAGACACAGAGACACATCTGGAAAGGTTGGGGAGGCTTGTCTCTGGCCAACCTCTCCCCGAATAATGGGGACCAACCCTGCTGTGTAGGTGGACAGCAGGCAGTGCCAGAGACAGACTAAGCCGTGGTCCCTGAGGTGGGGGTATGGGCCTTTCAAGGCAGTGTCTGGGATGAAGACACCTGATAAAGCCAGCTGGTACTCGGCTGTGGCCCACATGGGAAACCAGACAGTGTGGGTGAGGACGGGCCATTCTTCCTGCCAATAGATAACTTCCTCCTCTGGTCAGGACCCTGCCTCAGACAGGAGTGCACCTCTGCACCCACAGCAGTTTCTGCTCAGACTGCCTGTCTAACCCATTCTGCCTCTGAAGATCAAGGAGTGTGATCCCAGGAAGGTGGGGCTGTGGGCTACTCCACGGGAGTTGCTCCTCTCCCCTGAAATTCCATAatgagggcctgtgtgcttgtccatgtgctgaccatggtgggctttgccttggggaaggGTAAGTAAGGCCAGGGTCTGGAAAGAGTGGGGAGAGCTGGTCCTGGGTGTGCTTGAAAGACCTCCTGGTTCCGTCTCCTTTGTTCTCTGCGCCAAGctccggtcgccagcgtccgtacctgccacctgtgcttcttagaagacccttcgataggctgcatttctgggtcagaaaagtgcaccatcagctcttcctcgccatgcatggtgatcaccatctatcagaGTGAGCCAAGCCCCAGGAAAGGGCTGACGGTAGAGCAGTGTGGCTTGGTCTCCTCAACATAAGGGTGGTTTGGGCCACTCAAGGACGAGAGTTATCTTTGAAGGGTGGAGGGAGGGTGTGCAGACAAGTGAGCTGGTTATTTTTTTCTTTTCCCCCTCCACGAAATAAAACGAGGTCTGCGTGAGTGCGGGAAGAGTTAGGTTCGGGGACCGTGGGAAGCTGTTAACCAGGATTATAACTGTCCCCTCTCCTCTAGatgttaaggttcgcttccacgtacggggctgtggacagcaccactccttccggtgtcaagaaaatcacgtcatctactactcagactactggtatagggttaattgctgccagtatgattactgcaactcctggtccagtgcccagcaccagagcactctgcctgggcccccaggaaaccatctgggtgtgcccctctctgagtctcagataaaacagttctaccaggccctgcacctccctctgtttcagcctgacctccacactcataaggtgtctgagggcccggactctctcattctgcccccggggctgggcttgtccattgccgacctgcgcaaaatatacttgttcctcaacagttcaggacttctggttcttccccaggctagacccTGAcatgtcccatcccccttcccagattctgctctg

Amino acids

MSSSEEVSWISWFCGLRGNEFFCEVDEDYIQDKFNLTGLNEQVPHYRQALDMILDLEPDEELEDNPNQSDLIEQAAEMLYGLIHARYILTNRGIAQMLEKYQQGDFGYCPRVYCENQPMLPIGLSDIPGEAMVKLYCPKCMDVYTPKSSRHHHTDGAYFGTGFPHMLFMVHPEYRPKRPANQFVPRLYGFKIHPMAYQLQLQAASNFKSPVKTIR*

1.- Chimera MumCsnk2b-Ly6g5b-713 HE864440

Nucleotides

CCGCGGACATAAAGATGAGTAGCTCTGAGGAGGTGTCCTGGATTTCCTGGTTCTGTGGGCTCCGTGGTAATGAATTCTTCTGTGAGGTGGATGAAGACTACATCCAGGACAAATTTAATCTTACTGGACTCAATGAGCAGGTGCCTCACTATCGACAAGCTCTGGACATGATCTTAGACCTGGAACCTGATGAAGAGCTGGAAGACAACCCCAACCAGAGCGACTTGATCGAACAGGCAGCTGAGATGCTTTATGGGTTGATCCACGCCCGCTACATCCTCACCAACCGAGGCATCGCACAAATGATACTACTGTTCGCTTCCACGTACGGGGCTGTGGACAGCATCATTCCTACCGATGTCAAGAAAGGCATGTGATCTACCAATCAGACTACTTGTATAAGGCTGATTGCTGCCAGTACGATTACTGCAACTCCTGGTCCAGTGCTCAGCACCAGAGCACCCTGCGTGGGTCCCCAGGAAGCCATCTGGGCATGCCCCTGTCAGCGTCTCAGATAAAACAGTTTTACCAGGCCCTGAACCTCTCTCTGCCTCAGCCTGGCTTCCATGCTCATAAGGTGTCTGAGGGCCTGGAGTCTCTCATTCTGCCCCCGGAGCTGGGCTTGTCCATTGCCGACCTTCGCCAAATATACTTGTTCCTCAACAGTTCAGGACTTCTGGTCCTTCCCTGGGATAGACCCTGATGTGTTCCCC

Amino acids

MSSSEEVSWISWFCGLRGNEFFCEVDEDYIQDKFNLTGLNEQVPHYRQALDMILDLEPDEELEDNPNQSDLIEQAAEMLYGLIHARYILTNRGIAQMILLFASTYGAVDSIIPTDVKKGM*

2.- Chimera MumCsnk2b-Ly6g5b-979 HE864441

Nucleotides

CCGCGGACATAAAGATGAGTAGCTCTGAGGAGGTGTCCTGGATTTCCTGGTTCTGTGGGCTCCGTGGTAATGAATTCTTCTGTGAGGTGGATGAAGACTACATCCAGGACAAATTTAATCTTACTGGACTCAATGAGCAGGTGCCTCACTATCGACAAGCTCTGGACATGATCTTAGACCTGGAACCTGATGAAGAGCTGGAAGACAACCCCAACCAGAGCGACTTGATCGAACAGGCAGCTGAGATGCTTTATGGGTTGATCCACGCCCGCTACATCCTCACCAACCGAGGCATCGCACAAATGTTGGAAAAGTACCAGCAGGGAGACTTTGGCTACTGTCCTCGTGTATACTGTGAGAACCAGCCAATGCTTCCTATCGGCCTTTCAGACATCCCAGGCGAGGCCATGGTGAAACTCTACTGCCCCAAGTGCATGGACGTGTACACACCCAAGTCCTCCAGACACCACCACACGGACGGCGCATACTTCGGCACTGGTTTCCCTCACATGCTCTTCATGGTGCATCCAGAGTACCGGCCCAAGCGACCTGCCAACCAGTTTGTACCCAGATACTACTGTTCGCTTCCACGTACGGGGCTGTGGACAGCATCATTCCTACCGATGTCAAGAAAGGCATGTGATCTACCAATCAGACTACTTGTATAAGGCTGATTGCTGCCAGTACGATTACTGCAACTCCTGGTCCAGTGCTCAGCACCAGAGCACCCTGCGTGGGTCCCCAGGAAGCCATCTGGGCATGCCCCTGTCAGCGTCTCAGATAAAACAGTTTTACCAGGCCCTGAACCTCTCTCTGCCTCAGCCTGGCTTCCATGCTCATAAGGTGTCTGAGGGCCTGGAGTCTCTCATTCTGCCCCCGGAGCTGGGCTTGTCCATTGCCGACCTTCGCCAAATATACTTGTTCCTCAACAGTTCAGGACTTCTGGTCCTTCCCTGGGATAGACCCTGATGTGTTCCCC

Amino acids

MSSSEEVSWISWFCGLRGNEFFCEVDEDYIQDKFNLTGLNEQVPHYRQALDMILDLEPDEELEDNPNQSDLIEQAAEMLYGLIHARYILTNRGIAQMLEKYQQGDFGYCPRVYCENQPMLPIGLSDIPGEAMVKLYCPKCMDVYTPKSSRHHHTDGAYFGTGFPHMLFMVHPEYRPKRPANQFVPRYYCSLPRTGLWTASFLPMSRKACDLPIRLLV*

3.- Chimera MumCsnk2b-Ly6g5b-1108 HE864442

Nucleotides

CCGCGGACATAAAGATGAGTAGCTCTGAGGAGGTGTCCTGGATTTCCTGGTTCTGTGGGCTCCGTGGTAATGAATTCTTCTGTGAGGTGGATGAAGACTACATCCAGGACAAATTTAATCTTACTGGACTCAATGAGCAGGTGCCTCACTATCGACAAGCTCTGGACATGATCTTAGACCTGGAACCTGATGAAGAGCTGGAAGACAACCCCAACCAGAGCGACTTGATCGAACAGGCAGCTGAGATGCTTTATGGGTTGATCCACGCCCGCTACATCCTCACCAACCGAGGCATCGCACAAATGTTGGAAAAGTACCAGCAGGGAGACTTTGGCTACTGTCCTCGTGTATACTGTGAGAACCAGCCAATGCTTCCTATCGGCCTTTCAGACATCCCAGGCGAGGCCATGGTGAAACTCTACTGCCCCAAGTGCATGGACGTGTACACACCCAAGTCCTCCAGACACCACCACACGGACGGCGCATACTTCGGCACTGGTTTCCCTCACATGCTCTTCATGGTGCATCCAGAGTACCGGCCCAAGCGACCTGCCAACCAGTTTGTACCCAGCTCCAGTTGCCAGAGTCCGTACCTGCCACCTGTGCCTCTTAGAAGACCCTTCGCTAGGCTGCATTTCTGGCTCAGAAAAGTGCACCATCAGCCTTCCATCGCCGTGTATGGTGATCACCATCTATAAAAATACTACTGTTCGCTTCCACGTACGGGGCTGTGGACAGCATCATTCCTACCGATGTCAAGAAAGGCATGTGATCTACCAATCAGACTACTTGTATAAGGCTGATTGCTGCCAGTACGATTACTGCAACTCCTGGTCCAGTGCTCAGCACCAGAGCACCCTGCGTGGGTCCCCAGGAAGCCATCTGGGCATGCCCCTGTCAGCGTCTCAGATAAAACAGTTTTACCAGGCCCTGAACCTCTCTCTGCCTCAGCCTGGCTTCCATGCTCATAAGGTGTCTGAGGGCCTGGAGTCTCTCATTCTGCCCCCGGAGCTGGGCTTGTCCATTGCCGACCTTCGCCAAATATACTTGTTCCTCAACAGTTCAGGACTTCTGGTCCTTCCCTGGGATAGACCCTGATGTGTTCCCC

Amino acids

MSSSEEVSWISWFCGLRGNEFFCEVDEDYIQDKFNLTGLNEQVPHYRQALDMILDLEPDEELEDNPNQSDLIEQAAEMLYGLIHARYILTNRGIAQMLEKYQQGDFGYCPRVYCENQPMLPIGLSDIPGEAMVKLYCPKCMDVYTPKSSRHHHTDGAYFGTGFPHMLFMVHPEYRPKRPANQFVPSSSCQSPYLPPVPLRRPFARLHFWLRKVHHQPSIAVYGDHHL*
