# Supplementary material for: Intron retention and transcript chimerism conserved across mammals: Ly6g5b and Csnk2b-Ly6g5b as examples
Source: BMC Genomics. 2013 Mar 22;14:199. doi: 10.1186/1471-2164-14-199 (PMC3626593; doi:10.1186/1471-2164-14-199)
Supplement: Additional file 2: Table S1 — Table that contains all the different spliced isoforms detected in all the species studied in this work indicating whether they were previously described (deposited on databases) and, in the case they were found on databases the access number of the corresponding sequence or of the ESTs are indicated. [file 1471-2164-14-199-S2.doc]

| ***Homo sapiens*** | | | |
| --- | --- | --- | --- |
| **CSNK2B** | | | |
| ***Sequence name*** | ***Described before*** | ***Access number*** | |
|  |  | ***Sequence (nr/nt)*** | ***EST*** |
| HsCSNK2B-132 | NO |  |  |
| HsCSNK2B-247 | NO |  |  |
| HsCSNK2B-318 | NO |  |  |
| HsCSNK2B-660 | YES | [NM_001320](http://www.ncbi.nlm.nih.gov/entrez/viewer.fcgi?db=nuccore&val=26787971) | [gb\|CD049897.1\|](http://www.ncbi.nlm.nih.gov/entrez/query.fcgi?cmd=Retrieve&db=Nucleotide&list_uids=30486280&dopt=GenBank&RID=T8DH8VGE01R&log$=nucltop&blast_rank=1) [gb\|BQ068404.1\|](http://www.ncbi.nlm.nih.gov/entrez/query.fcgi?cmd=Retrieve&db=Nucleotide&list_uids=19897450&dopt=GenBank&RID=T8DH8VGE01R&log$=nucltop&blast_rank=2)  [gb\|BI087444.1\|](http://www.ncbi.nlm.nih.gov/entrez/query.fcgi?cmd=Retrieve&db=Nucleotide&list_uids=14505774&dopt=GenBank&RID=T8DH8VGE01R&log$=nucltop&blast_rank=3) [emb\|CT001472.1\|](http://www.ncbi.nlm.nih.gov/entrez/query.fcgi?cmd=Retrieve&db=Nucleotide&list_uids=68295355&dopt=GenBank&RID=T8DH8VGE01R&log$=nucltop&blast_rank=4)  [emb\|CR990719.1\|](http://www.ncbi.nlm.nih.gov/entrez/query.fcgi?cmd=Retrieve&db=Nucleotide&list_uids=68284604&dopt=GenBank&RID=T8DH8VGE01R&log$=nucltop&blast_rank=5) [emb\|BX363938.2\|](http://www.ncbi.nlm.nih.gov/entrez/query.fcgi?cmd=Retrieve&db=Nucleotide&list_uids=46291550&dopt=GenBank&RID=T8DH8VGE01R&log$=nucltop&blast_rank=6)  [gb\|BU149588.1\|](http://www.ncbi.nlm.nih.gov/entrez/query.fcgi?cmd=Retrieve&db=Nucleotide&list_uids=22663120&dopt=GenBank&RID=T8DH8VGE01R&log$=nucltop&blast_rank=7) [gb\|BQ897909.1\|](http://www.ncbi.nlm.nih.gov/entrez/query.fcgi?cmd=Retrieve&db=Nucleotide&list_uids=22289923&dopt=GenBank&RID=T8DH8VGE01R&log$=nucltop&blast_rank=8)  [gb\|BQ643279.1\|](http://www.ncbi.nlm.nih.gov/entrez/query.fcgi?cmd=Retrieve&db=Nucleotide&list_uids=21767451&dopt=GenBank&RID=T8DH8VGE01R&log$=nucltop&blast_rank=9) [gb\|BQ065124.1\|](http://www.ncbi.nlm.nih.gov/entrez/query.fcgi?cmd=Retrieve&db=Nucleotide&list_uids=19894170&dopt=GenBank&RID=T8DH8VGE01R&log$=nucltop&blast_rank=10)  [gb\|BQ053701.1\|](http://www.ncbi.nlm.nih.gov/entrez/query.fcgi?cmd=Retrieve&db=Nucleotide&list_uids=19813028&dopt=GenBank&RID=T8DH8VGE01R&log$=nucltop&blast_rank=11) [gb\|BM915528.1](http://www.ncbi.nlm.nih.gov/entrez/query.fcgi?cmd=Retrieve&db=Nucleotide&list_uids=19365907&dopt=GenBank&RID=T8DH8VGE01R&log$=nucltop&blast_rank=12) [gb\|BM554459.1\|](http://www.ncbi.nlm.nih.gov/entrez/query.fcgi?cmd=Retrieve&db=Nucleotide&list_uids=18794074&dopt=GenBank&RID=T8DH8VGE01R&log$=nucltop&blast_rank=13) [gb\|BE799225.1\|](http://www.ncbi.nlm.nih.gov/entrez/query.fcgi?cmd=Retrieve&db=Nucleotide&list_uids=10220423&dopt=GenBank&RID=T8DH8VGE01R&log$=nucltop&blast_rank=14) [gb\|BE799188.1\|](http://www.ncbi.nlm.nih.gov/entrez/query.fcgi?cmd=Retrieve&db=Nucleotide&list_uids=10220386&dopt=GenBank&RID=T8DH8VGE01R&log$=nucltop&blast_rank=15) [emb\|CR737821.1\|](http://www.ncbi.nlm.nih.gov/entrez/query.fcgi?cmd=Retrieve&db=Nucleotide&list_uids=51586896&dopt=GenBank&RID=T8DH8VGE01R&log$=nucltop&blast_rank=16) [emb\|BX379662.2\|](http://www.ncbi.nlm.nih.gov/entrez/query.fcgi?cmd=Retrieve&db=Nucleotide&list_uids=46557555&dopt=GenBank&RID=T8DH8VGE01R&log$=nucltop&blast_rank=17) [gb\|CD049380.1\|](http://www.ncbi.nlm.nih.gov/entrez/query.fcgi?cmd=Retrieve&db=Nucleotide&list_uids=30485265&dopt=GenBank&RID=T8DH8VGE01R&log$=nucltop&blast_rank=18) [gb\|BQ957054.1\|](http://www.ncbi.nlm.nih.gov/entrez/query.fcgi?cmd=Retrieve&db=Nucleotide&list_uids=22372532&dopt=GenBank&RID=T8DH8VGE01R&log$=nucltop&blast_rank=19) [gb\|BQ936869.1\|](http://www.ncbi.nlm.nih.gov/entrez/query.fcgi?cmd=Retrieve&db=Nucleotide&list_uids=22352252&dopt=GenBank&RID=T8DH8VGE01R&log$=nucltop&blast_rank=20) [gb\|BM808196.1\|](http://www.ncbi.nlm.nih.gov/entrez/query.fcgi?cmd=Retrieve&db=Nucleotide&list_uids=19125019&dopt=GenBank&RID=T8DH8VGE01R&log$=nucltop&blast_rank=21) [gb\|BI766841.1\|](http://www.ncbi.nlm.nih.gov/entrez/query.fcgi?cmd=Retrieve&db=Nucleotide&list_uids=15758419&dopt=GenBank&RID=T8DH8VGE01R&log$=nucltop&blast_rank=22) [gb\|BQ897257.1\|](http://www.ncbi.nlm.nih.gov/entrez/query.fcgi?cmd=Retrieve&db=Nucleotide&list_uids=22289271&dopt=GenBank&RID=T8DH8VGE01R&log$=nucltop&blast_rank=23) [gb\|BQ686927.1\|](http://www.ncbi.nlm.nih.gov/entrez/query.fcgi?cmd=Retrieve&db=Nucleotide&list_uids=21812243&dopt=GenBank&RID=T8DH8VGE01R&log$=nucltop&blast_rank=24) [emb\|BX354986.2\|](http://www.ncbi.nlm.nih.gov/entrez/query.fcgi?cmd=Retrieve&db=Nucleotide&list_uids=46306406&dopt=GenBank&RID=T8DH8VGE01R&log$=nucltop&blast_rank=25) [emb\|BX363937.2\|](http://www.ncbi.nlm.nih.gov/entrez/query.fcgi?cmd=Retrieve&db=Nucleotide&list_uids=46289682&dopt=GenBank&RID=T8DH8VGE01R&log$=nucltop&blast_rank=26) [gb\|BQ278396.1\|](http://www.ncbi.nlm.nih.gov/entrez/query.fcgi?cmd=Retrieve&db=Nucleotide&list_uids=20488604&dopt=GenBank&RID=T8DH8VGE01R&log$=nucltop&blast_rank=27) [gb\|BM009485.1\|](http://www.ncbi.nlm.nih.gov/entrez/query.fcgi?cmd=Retrieve&db=Nucleotide&list_uids=16523839&dopt=GenBank&RID=T8DH8VGE01R&log$=nucltop&blast_rank=28) [gb\|BI667289.1\|](http://www.ncbi.nlm.nih.gov/entrez/query.fcgi?cmd=Retrieve&db=Nucleotide&list_uids=15581522&dopt=GenBank&RID=T8DH8VGE01R&log$=nucltop&blast_rank=29) [gb\|BU187324.1\|](http://www.ncbi.nlm.nih.gov/entrez/query.fcgi?cmd=Retrieve&db=Nucleotide&list_uids=22701308&dopt=GenBank&RID=T8DH8VGE01R&log$=nucltop&blast_rank=30) [gb\|BQ433003.1\|](http://www.ncbi.nlm.nih.gov/entrez/query.fcgi?cmd=Retrieve&db=Nucleotide&list_uids=21172079&dopt=GenBank&RID=T8DH8VGE01R&log$=nucltop&blast_rank=31) [gb\|BM809095.1\|](http://www.ncbi.nlm.nih.gov/entrez/query.fcgi?cmd=Retrieve&db=Nucleotide&list_uids=19125918&dopt=GenBank&RID=T8DH8VGE01R&log$=nucltop&blast_rank=32) [gb\|BM562580.1\|](http://www.ncbi.nlm.nih.gov/entrez/query.fcgi?cmd=Retrieve&db=Nucleotide&list_uids=18808791&dopt=GenBank&RID=T8DH8VGE01R&log$=nucltop&blast_rank=33) [gb\|BI222427.1\|](http://www.ncbi.nlm.nih.gov/entrez/query.fcgi?cmd=Retrieve&db=Nucleotide&list_uids=14675871&dopt=GenBank&RID=T8DH8VGE01R&log$=nucltop&blast_rank=34) [gb\|BE902052.1\|](http://www.ncbi.nlm.nih.gov/entrez/query.fcgi?cmd=Retrieve&db=Nucleotide&list_uids=10391846&dopt=GenBank&RID=T8DH8VGE01R&log$=nucltop&blast_rank=35) [gb\|BQ052701.1\|](http://www.ncbi.nlm.nih.gov/entrez/query.fcgi?cmd=Retrieve&db=Nucleotide&list_uids=19812041&dopt=GenBank&RID=T8DH8VGE01R&log$=nucltop&blast_rank=36) [gb\|CD050161.1\|](http://www.ncbi.nlm.nih.gov/entrez/query.fcgi?cmd=Retrieve&db=Nucleotide&list_uids=30486833&dopt=GenBank&RID=T8DH8VGE01R&log$=nucltop&blast_rank=37) [gb\|BQ672055.1\|](http://www.ncbi.nlm.nih.gov/entrez/query.fcgi?cmd=Retrieve&db=Nucleotide&list_uids=21782889&dopt=GenBank&RID=T8DH8VGE01R&log$=nucltop&blast_rank=38) [gb\|BQ644790.1\|](http://www.ncbi.nlm.nih.gov/entrez/query.fcgi?cmd=Retrieve&db=Nucleotide&list_uids=21768962&dopt=GenBank&RID=T8DH8VGE01R&log$=nucltop&blast_rank=39) [gb\|BI561198.1\|](http://www.ncbi.nlm.nih.gov/entrez/query.fcgi?cmd=Retrieve&db=Nucleotide&list_uids=15448512&dopt=GenBank&RID=T8DH8VGE01R&log$=nucltop&blast_rank=40) [gb\|BQ109206.1\|](http://www.ncbi.nlm.nih.gov/entrez/query.fcgi?cmd=Retrieve&db=Nucleotide&list_uids=20158860&dopt=GenBank&RID=T8DH8VGE01R&log$=nucltop&blast_rank=41) [gb\|BI087366.1\|](http://www.ncbi.nlm.nih.gov/entrez/query.fcgi?cmd=Retrieve&db=Nucleotide&list_uids=14505696&dopt=GenBank&RID=T8DH8VGE01R&log$=nucltop&blast_rank=42) [emb\|BX324443.2\|](http://www.ncbi.nlm.nih.gov/entrez/query.fcgi?cmd=Retrieve&db=Nucleotide&list_uids=46274275&dopt=GenBank&RID=T8DH8VGE01R&log$=nucltop&blast_rank=43) [gb\|BG766661.1\|](http://www.ncbi.nlm.nih.gov/entrez/query.fcgi?cmd=Retrieve&db=Nucleotide&list_uids=14077314&dopt=GenBank&RID=T8DH8VGE01R&log$=nucltop&blast_rank=44) [gb\|BE741535.1\|](http://www.ncbi.nlm.nih.gov/entrez/query.fcgi?cmd=Retrieve&db=Nucleotide&list_uids=10155527&dopt=GenBank&RID=T8DH8VGE01R&log$=nucltop&blast_rank=45) [gb\|BE618864.1\|](http://www.ncbi.nlm.nih.gov/entrez/query.fcgi?cmd=Retrieve&db=Nucleotide&list_uids=9889802&dopt=GenBank&RID=T8DH8VGE01R&log$=nucltop&blast_rank=46) [gb\|BG388624.1\|](http://www.ncbi.nlm.nih.gov/entrez/query.fcgi?cmd=Retrieve&db=Nucleotide&list_uids=13282070&dopt=GenBank&RID=T8DH8VGE01R&log$=nucltop&blast_rank=47) [gb\|BG480624.1\|](http://www.ncbi.nlm.nih.gov/entrez/query.fcgi?cmd=Retrieve&db=Nucleotide&list_uids=13412903&dopt=GenBank&RID=T8DH8VGE01R&log$=nucltop&blast_rank=48) [emb\|BX364388.2\|](http://www.ncbi.nlm.nih.gov/entrez/query.fcgi?cmd=Retrieve&db=Nucleotide&list_uids=46307740&dopt=GenBank&RID=T8DH8VGE01R&log$=nucltop&blast_rank=49) [gb\|BE748721.1\|](http://www.ncbi.nlm.nih.gov/entrez/query.fcgi?cmd=Retrieve&db=Nucleotide&list_uids=10162713&dopt=GenBank&RID=T8DH8VGE01R&log$=nucltop&blast_rank=50) [gb\|BM010516.1\|](http://www.ncbi.nlm.nih.gov/entrez/query.fcgi?cmd=Retrieve&db=Nucleotide&list_uids=16524870&dopt=GenBank&RID=T8DH8VGE01R&log$=nucltop&blast_rank=51) [gb\|BM554000.1\|](http://www.ncbi.nlm.nih.gov/entrez/query.fcgi?cmd=Retrieve&db=Nucleotide&list_uids=18793230&dopt=GenBank&RID=T8DH8VGE01R&log$=nucltop&blast_rank=52) [gb\|BE740450.1\|](http://www.ncbi.nlm.nih.gov/entrez/query.fcgi?cmd=Retrieve&db=Nucleotide&list_uids=10154442&dopt=GenBank&RID=T8DH8VGE01R&log$=nucltop&blast_rank=53) [gb\|BG425664.1\|](http://www.ncbi.nlm.nih.gov/entrez/query.fcgi?cmd=Retrieve&db=Nucleotide&list_uids=13332170&dopt=GenBank&RID=T8DH8VGE01R&log$=nucltop&blast_rank=54) [gb\|BU500603.1\|](http://www.ncbi.nlm.nih.gov/entrez/query.fcgi?cmd=Retrieve&db=Nucleotide&list_uids=22801760&dopt=GenBank&RID=T8DH8VGE01R&log$=nucltop&blast_rank=55) [gb\|BG827338.1\|](http://www.ncbi.nlm.nih.gov/entrez/query.fcgi?cmd=Retrieve&db=Nucleotide&list_uids=14174925&dopt=GenBank&RID=T8DH8VGE01R&log$=nucltop&blast_rank=56) [gb\|DN997730.1\|](http://www.ncbi.nlm.nih.gov/entrez/query.fcgi?cmd=Retrieve&db=Nucleotide&list_uids=66257557&dopt=GenBank&RID=T8DH8VGE01R&log$=nucltop&blast_rank=57) [gb\|BF690392.1\|](http://www.ncbi.nlm.nih.gov/entrez/query.fcgi?cmd=Retrieve&db=Nucleotide&list_uids=11975800&dopt=GenBank&RID=T8DH8VGE01R&log$=nucltop&blast_rank=58) [gb\|BE790602.1\|](http://www.ncbi.nlm.nih.gov/entrez/query.fcgi?cmd=Retrieve&db=Nucleotide&list_uids=10211800&dopt=GenBank&RID=T8DH8VGE01R&log$=nucltop&blast_rank=59) [gb\|BE907398.1\|](http://www.ncbi.nlm.nih.gov/entrez/query.fcgi?cmd=Retrieve&db=Nucleotide&list_uids=10400917&dopt=GenBank&RID=T8DH8VGE01R&log$=nucltop&blast_rank=60) [gb\|BQ643177.1\|](http://www.ncbi.nlm.nih.gov/entrez/query.fcgi?cmd=Retrieve&db=Nucleotide&list_uids=21767349&dopt=GenBank&RID=T8DH8VGE01R&log$=nucltop&blast_rank=61) [gb\|BI857525.1\|](http://www.ncbi.nlm.nih.gov/entrez/query.fcgi?cmd=Retrieve&db=Nucleotide&list_uids=15998272&dopt=GenBank&RID=T8DH8VGE01R&log$=nucltop&blast_rank=62) [gb\|BI598730.1\|](http://www.ncbi.nlm.nih.gov/entrez/query.fcgi?cmd=Retrieve&db=Nucleotide&list_uids=15491669&dopt=GenBank&RID=T8DH8VGE01R&log$=nucltop&blast_rank=63) [emb\|BX354985.2\|](http://www.ncbi.nlm.nih.gov/entrez/query.fcgi?cmd=Retrieve&db=Nucleotide&list_uids=46304545&dopt=GenBank&RID=T8DH8VGE01R&log$=nucltop&blast_rank=64) [gb\|BM809286.1\|](http://www.ncbi.nlm.nih.gov/entrez/query.fcgi?cmd=Retrieve&db=Nucleotide&list_uids=19126109&dopt=GenBank&RID=T8DH8VGE01R&log$=nucltop&blast_rank=65) [gb\|BG324412.1\|](http://www.ncbi.nlm.nih.gov/entrez/query.fcgi?cmd=Retrieve&db=Nucleotide&list_uids=13130849&dopt=GenBank&RID=T8DH8VGE01R&log$=nucltop&blast_rank=66) [gb\|BE618379.1\|](http://www.ncbi.nlm.nih.gov/entrez/query.fcgi?cmd=Retrieve&db=Nucleotide&list_uids=9889317&dopt=GenBank&RID=T8DH8VGE01R&log$=nucltop&blast_rank=67) [gb\|BG289474.1\|](http://www.ncbi.nlm.nih.gov/entrez/query.fcgi?cmd=Retrieve&db=Nucleotide&list_uids=13045353&dopt=GenBank&RID=T8DH8VGE01R&log$=nucltop&blast_rank=68) [gb\|BE745233.1\|](http://www.ncbi.nlm.nih.gov/entrez/query.fcgi?cmd=Retrieve&db=Nucleotide&list_uids=10159225&dopt=GenBank&RID=T8DH8VGE01R&log$=nucltop&blast_rank=69) [gb\|BU595112.1\|](http://www.ncbi.nlm.nih.gov/entrez/query.fcgi?cmd=Retrieve&db=Nucleotide&list_uids=23246871&dopt=GenBank&RID=T8DH8VGE01R&log$=nucltop&blast_rank=70) [gb\|BQ423186.1\|](http://www.ncbi.nlm.nih.gov/entrez/query.fcgi?cmd=Retrieve&db=Nucleotide&list_uids=21118501&dopt=GenBank&RID=T8DH8VGE01R&log$=nucltop&blast_rank=71) [gb\|BE794590.1\|](http://www.ncbi.nlm.nih.gov/entrez/query.fcgi?cmd=Retrieve&db=Nucleotide&list_uids=10215788&dopt=GenBank&RID=T8DH8VGE01R&log$=nucltop&blast_rank=72) [gb\|BE745737.1\|](http://www.ncbi.nlm.nih.gov/entrez/query.fcgi?cmd=Retrieve&db=Nucleotide&list_uids=10159729&dopt=GenBank&RID=T8DH8VGE01R&log$=nucltop&blast_rank=73) [gb\|BG760290.1\|](http://www.ncbi.nlm.nih.gov/entrez/query.fcgi?cmd=Retrieve&db=Nucleotide&list_uids=14070943&dopt=GenBank&RID=T8DH8VGE01R&log$=nucltop&blast_rank=74) [gb\|BG829513.1\|](http://www.ncbi.nlm.nih.gov/entrez/query.fcgi?cmd=Retrieve&db=Nucleotide&list_uids=14177196&dopt=GenBank&RID=T8DH8VGE01R&log$=nucltop&blast_rank=75) [gb\|BG752112.1\|](http://www.ncbi.nlm.nih.gov/entrez/query.fcgi?cmd=Retrieve&db=Nucleotide&list_uids=14062765&dopt=GenBank&RID=T8DH8VGE01R&log$=nucltop&blast_rank=76) [gb\|BE730656.1\|](http://www.ncbi.nlm.nih.gov/entrez/query.fcgi?cmd=Retrieve&db=Nucleotide&list_uids=10144648&dopt=GenBank&RID=T8DH8VGE01R&log$=nucltop&blast_rank=77) [gb\|BU517210.1\|](http://www.ncbi.nlm.nih.gov/entrez/query.fcgi?cmd=Retrieve&db=Nucleotide&list_uids=22824736&dopt=GenBank&RID=T8DH8VGE01R&log$=nucltop&blast_rank=78) [gb\|BU181313.1\|](http://www.ncbi.nlm.nih.gov/entrez/query.fcgi?cmd=Retrieve&db=Nucleotide&list_uids=22695297&dopt=GenBank&RID=T8DH8VGE01R&log$=nucltop&blast_rank=79) [gb\|BE732626.1\|](http://www.ncbi.nlm.nih.gov/entrez/query.fcgi?cmd=Retrieve&db=Nucleotide&list_uids=10146630&dopt=GenBank&RID=T8DH8VGE01R&log$=nucltop&blast_rank=80) [gb\|BE902235.1\|](http://www.ncbi.nlm.nih.gov/entrez/query.fcgi?cmd=Retrieve&db=Nucleotide&list_uids=10392220&dopt=GenBank&RID=T8DH8VGE01R&log$=nucltop&blast_rank=81) [gb\|BE314406.1\|](http://www.ncbi.nlm.nih.gov/entrez/query.fcgi?cmd=Retrieve&db=Nucleotide&list_uids=9135495&dopt=GenBank&RID=T8DH8VGE01R&log$=nucltop&blast_rank=82)  [gb\|BF975103.1\|](http://www.ncbi.nlm.nih.gov/entrez/query.fcgi?cmd=Retrieve&db=Nucleotide&list_uids=12342318&dopt=GenBank&RID=T8DH8VGE01R&log$=nucltop&blast_rank=83) [gb\|BE302855.1\|](http://www.ncbi.nlm.nih.gov/entrez/query.fcgi?cmd=Retrieve&db=Nucleotide&list_uids=9186603&dopt=GenBank&RID=T8DH8VGE01R&log$=nucltop&blast_rank=84) [gb\|AI190314.1\|](http://www.ncbi.nlm.nih.gov/entrez/query.fcgi?cmd=Retrieve&db=Nucleotide&list_uids=3741523&dopt=GenBank&RID=T8DH8VGE01R&log$=nucltop&blast_rank=85) [gb\|CN431456.1\|](http://www.ncbi.nlm.nih.gov/entrez/query.fcgi?cmd=Retrieve&db=Nucleotide&list_uids=47419050&dopt=GenBank&RID=T8DH8VGE01R&log$=nucltop&blast_rank=86) [emb\|BX379661.2\|](http://www.ncbi.nlm.nih.gov/entrez/query.fcgi?cmd=Retrieve&db=Nucleotide&list_uids=46555774&dopt=GenBank&RID=T8DH8VGE01R&log$=nucltop&blast_rank=87) [gb\|BF338390.1\|](http://www.ncbi.nlm.nih.gov/entrez/query.fcgi?cmd=Retrieve&db=Nucleotide&list_uids=11284790&dopt=GenBank&RID=T8DH8VGE01R&log$=nucltop&blast_rank=88) [gb\|BG913370.1\|](http://www.ncbi.nlm.nih.gov/entrez/query.fcgi?cmd=Retrieve&db=Nucleotide&list_uids=14293846&dopt=GenBank&RID=T8DH8VGE01R&log$=nucltop&blast_rank=89) [gb\|BG421874.1\|](http://www.ncbi.nlm.nih.gov/entrez/query.fcgi?cmd=Retrieve&db=Nucleotide&list_uids=13328380&dopt=GenBank&RID=T8DH8VGE01R&log$=nucltop&blast_rank=90) [gb\|BE891433.1\|](http://www.ncbi.nlm.nih.gov/entrez/query.fcgi?cmd=Retrieve&db=Nucleotide&list_uids=10350762&dopt=GenBank&RID=T8DH8VGE01R&log$=nucltop&blast_rank=91) [gb\|BQ929648.1\|](http://www.ncbi.nlm.nih.gov/entrez/query.fcgi?cmd=Retrieve&db=Nucleotide&list_uids=22344679&dopt=GenBank&RID=T8DH8VGE01R&log$=nucltop&blast_rank=92) [gb\|BQ686681.1\|](http://www.ncbi.nlm.nih.gov/entrez/query.fcgi?cmd=Retrieve&db=Nucleotide&list_uids=21811997&dopt=GenBank&RID=T8DH8VGE01R&log$=nucltop&blast_rank=93) [gb\|BU182212.1\|](http://www.ncbi.nlm.nih.gov/entrez/query.fcgi?cmd=Retrieve&db=Nucleotide&list_uids=22696196&dopt=GenBank&RID=T8DH8VGE01R&log$=nucltop&blast_rank=94) [gb\|BG024416.1\|](http://www.ncbi.nlm.nih.gov/entrez/query.fcgi?cmd=Retrieve&db=Nucleotide&list_uids=12409969&dopt=GenBank&RID=T8DH8VGE01R&log$=nucltop&blast_rank=95) [gb\|BE548414.1\|](http://www.ncbi.nlm.nih.gov/entrez/query.fcgi?cmd=Retrieve&db=Nucleotide&list_uids=9777059&dopt=GenBank&RID=T8DH8VGE01R&log$=nucltop&blast_rank=96) [gb\|BI833971.1\|](http://www.ncbi.nlm.nih.gov/entrez/query.fcgi?cmd=Retrieve&db=Nucleotide&list_uids=15945521&dopt=GenBank&RID=T8DH8VGE01R&log$=nucltop&blast_rank=97) [gb\|BM804367.1\|](http://www.ncbi.nlm.nih.gov/entrez/query.fcgi?cmd=Retrieve&db=Nucleotide&list_uids=19121190&dopt=GenBank&RID=T8DH8VGE01R&log$=nucltop&blast_rank=98) [gb\|CN431455.1\|](http://www.ncbi.nlm.nih.gov/entrez/query.fcgi?cmd=Retrieve&db=Nucleotide&list_uids=47419049&dopt=GenBank&RID=T8DH8VGE01R&log$=nucltop&blast_rank=99) [gb\|BQ428316.1\|](http://www.ncbi.nlm.nih.gov/entrez/query.fcgi?cmd=Retrieve&db=Nucleotide&list_uids=21167392&dopt=GenBank&RID=T8DH8VGE01R&log$=nucltop&blast_rank=100) [gb\|BE799188.1\|](http://www.ncbi.nlm.nih.gov/entrez/query.fcgi?cmd=Retrieve&db=Nucleotide&list_uids=10220386&dopt=GenBank&RID=T8DH8VGE01R&log$=nucltop&blast_rank=15) |
| HsCSNK2B-806 | NO |  |  |
| **LY6G5B** | | | |
| ***Sequence name*** | ***Described before*** | ***Access number*** | |
|  |  | ***Sequence (nr/nt)*** | ***EST*** |
| HsLY6G5B-288 | NO |  |  |
| HsLY6G5B-452 | NO |  |  |
| HsLY6G5B-690 | YES | [NM_021221](http://www.ncbi.nlm.nih.gov/entrez/viewer.fcgi?db=nuccore&val=50845398) | [gb\|CF264683.1\|](http://www.ncbi.nlm.nih.gov/entrez/query.fcgi?cmd=Retrieve&db=Nucleotide&list_uids=33605062&dopt=GenBank&RID=T8ENVKUM011&log$=nuclalign&blast_rank=1) |
| HsLY6G5B-837 | YES | [AJ245417](http://www.ncbi.nlm.nih.gov/entrez/viewer.fcgi?db=nuccore&val=5701853). | [emb\|CT001189.1\|](http://www.ncbi.nlm.nih.gov/entrez/query.fcgi?cmd=Retrieve&db=Nucleotide&list_uids=68295072&dopt=GenBank&RID=T8EU18PY011&log$=nuclalign&blast_rank=7) [gb\|BF820976.1\|](http://www.ncbi.nlm.nih.gov/entrez/query.fcgi?cmd=Retrieve&db=Nucleotide&list_uids=12159852&dopt=GenBank&RID=T8EU18PY011&log$=nuclalign&blast_rank=12) |
| **Chimera CSNK2B- LY6G5B** | | | |
| ***Sequence name*** | ***Described before*** | ***Access number*** | |
|  |  | ***Sequence (nr/nt)*** | ***EST*** |
| HsChimera CSNK2B-LY6G5B-182 | NO |  |  |
| HsChimera CSNK2B-LY6G5B-532 | NO |  |  |
| HsChimera CSNK2B-LY6G5B-560 | NO |  |  |
| HsChimera CSNK2B-LY6G5B-562 | NO |  |  |
| HsChimera CSNK2B-LY6G5B-696 | NO |  |  |
| HsChimera CSNK2B-LY6G5B-991 | NO |  |  |
| HsChimera CSNK2B-LY6G5B-1072 | NO |  |  |
| HsChimera CSNK2B-LY6G5B-1103 | NO |  |  |
| HsChimera CSNK2B-LY6G5B-1181 | YES | [CR598133](http://www.ncbi.nlm.nih.gov/entrez/viewer.fcgi?db=nuccore&val=50478940) | [emb\|BX363222.2\|](http://www.ncbi.nlm.nih.gov/entrez/query.fcgi?cmd=Retrieve&db=Nucleotide&list_uids=46289654&dopt=GenBank&RID=T8F5PEYM011&log$=nuclalign&blast_rank=1) |
| HsChimera CSNK2B-LY6G5B-1327 | NO |  |  |

| ***Macaca mulatta*** | | | |
| --- | --- | --- | --- |
| **Csnk2b** | | | |
| ***Sequence name*** | ***Described before*** | ***Access number*** | |
|  |  | ***Sequence (nr/nt)*** | ***EST*** |
| MamCsnk2b-205 | NO |  |  |
| MamCsnk2b-238 | NO |  |  |
| MamCsnk2b-274 | NO |  |  |
| MamCsnk2b-660 | YES | [XM_001112478](http://www.ncbi.nlm.nih.gov/entrez/viewer.fcgi?db=nuccore&val=109070471) [XM_001112540](http://www.ncbi.nlm.nih.gov/entrez/viewer.fcgi?db=nuccore&val=109070469) | [DR770050](http://www.ncbi.nlm.nih.gov/entrez/viewer.fcgi?db=nuccore&val=71110388) [DV769649](http://www.ncbi.nlm.nih.gov/entrez/viewer.fcgi?db=nuccore&val=82696311) |
| **Ly6g5b** | | | |
| ***Sequence name*** | ***Described before*** | ***Access number*** | |
|  |  | ***Sequence (nr/nt)*** | ***EST*** |
| MamLy6g5b-325 | NO |  |  |
| MamLy6g5b-837 | NO |  |  |
| **Chimera Csnk2b-Ly6g5b** | | | |
| ***Sequence name*** | ***Described before*** | ***Access number*** | |
|  |  | ***Sequence (nr/nt)*** | ***EST*** |
| MamChimera Csnk2b-Ly6g5b-992 | NO |  |  |
| MamChimera Csnk2b-Ly6g5b-1141 | NO |  |  |
| MamChimera Csnk2b-Ly6g5b-1218 | NO |  |  |
| MamChimera Csnk2b-Ly6g5b-1331 | NO |  |  |
| MamChimera Csnk2b-Ly6g5b-2338 | NO |  |  |

| ***Sus scrofa*** | | | |
| --- | --- | --- | --- |
| **Csnk2b** | | | |
| ***Sequence name*** | ***Described before*** | ***Access number*** | |
|  |  | ***Sequence (nr/nt)*** | ***EST*** |
| SsCsnk2b-204 | NO |  |  |
| SsCsnk2b-324 | NO |  |  |
| SsCsnk2b-476 | NO |  |  |
| SsCsnk2b-666 | YES | [EU282347.1](http://www.ncbi.nlm.nih.gov/entrez/query.fcgi?cmd=Retrieve&db=Nucleotide&list_uids=162138219&dopt=GenBank&RID=URM8X2YB013&log$=nucltop&blast_rank=1) | [gb\|CN154983.1\|](http://www.ncbi.nlm.nih.gov/entrez/query.fcgi?cmd=Retrieve&db=Nucleotide&list_uids=46169413&dopt=GenBank&RID=URM97NU9012&log$=nucltop&blast_rank=1) [gb\|DN111998.1\|](http://www.ncbi.nlm.nih.gov/entrez/query.fcgi?cmd=Retrieve&db=Nucleotide&list_uids=59790664&dopt=GenBank&RID=URM97NU9012&log$=nucltop&blast_rank=2)  [gb\|DN111633.1\|](http://www.ncbi.nlm.nih.gov/entrez/query.fcgi?cmd=Retrieve&db=Nucleotide&list_uids=59790299&dopt=GenBank&RID=URM97NU9012&log$=nucltop&blast_rank=3)  [gb\|DY406067.1\|](http://www.ncbi.nlm.nih.gov/entrez/query.fcgi?cmd=Retrieve&db=Nucleotide&list_uids=87207370&dopt=GenBank&RID=URM97NU9012&log$=nucltop&blast_rank=4)  [gb\|DY417390.1\|](http://www.ncbi.nlm.nih.gov/entrez/query.fcgi?cmd=Retrieve&db=Nucleotide&list_uids=87218693&dopt=GenBank&RID=URM97NU9012&log$=nucltop&blast_rank=5)  [gb\|DY404732.1\|](http://www.ncbi.nlm.nih.gov/entrez/query.fcgi?cmd=Retrieve&db=Nucleotide&list_uids=87206035&dopt=GenBank&RID=URM97NU9012&log$=nucltop&blast_rank=6)  [gb\|CN167142.1\|](http://www.ncbi.nlm.nih.gov/entrez/query.fcgi?cmd=Retrieve&db=Nucleotide&list_uids=46181572&dopt=GenBank&RID=URM97NU9012&log$=nucltop&blast_rank=7)  [dbj\|DB809985.1\|](http://www.ncbi.nlm.nih.gov/entrez/query.fcgi?cmd=Retrieve&db=Nucleotide&list_uids=112756629&dopt=GenBank&RID=URM97NU9012&log$=nucltop&blast_rank=13) |
| **Ly6g5b** | | | |
| ***Sequence name*** | ***Described before*** | ***Access number*** | |
|  |  | ***Sequence (nr/nt)*** | ***EST*** |
| SsLy6g5b-219 | YES |  | [emb\|BX673504.2\|](http://www.ncbi.nlm.nih.gov/entrez/query.fcgi?cmd=Retrieve&db=Nucleotide&list_uids=90242004&dopt=GenBank&RID=UU6JKTU2016&log$=nucltop&blast_rank=1) |
| SsLy6g5b-325 | NO |  |  |
| SsLy6g5b-714 | NO |  |  |
| **Chimera Csnk2b-Ly6g5b** | | | |
| ***Sequence name*** | ***Described before*** | ***Access number*** | |
|  |  | ***Sequence (nr/nt)*** | ***EST*** |
| SsChimera Csnk2b-Ly6g5b-538 | NO |  |  |
| SsChimera Csnk2b-Ly6g5b-728 | NO |  |  |
| SsChimera Csnk2b-Ly6g5b-927 | NO |  |  |

| ***Bos taurus*** | | | |
| --- | --- | --- | --- |
| **Csnk2b** | | | |
| ***Sequence name*** | ***Described before*** | ***Access number*** | |
|  |  | ***Sequence (nr/nt)*** | ***EST*** |
| BtCsnk2b-191 | NO |  |  |
| BtCsnk2b-242 | NO |  |  |
| BtCsnk2b-281 | NO |  |  |
| BtCsnk2b-473 | YES |  | [gb\|EH156085.1\|](http://www.ncbi.nlm.nih.gov/entrez/query.fcgi?cmd=Retrieve&db=Nucleotide&list_uids=119555090&dopt=GenBank&RID=UUTZCSKR012&log$=nucltop&blast_rank=1) |
| BtCsnk2b-663 | YES | [BC110170.1](http://www.ncbi.nlm.nih.gov/entrez/query.fcgi?cmd=Retrieve&db=Nucleotide&list_uids=82571787&dopt=GenBank&RID=UUU7N0S6016&log$=nucltop&blast_rank=1) | [gb\|EV742136.1\|](http://www.ncbi.nlm.nih.gov/entrez/query.fcgi?cmd=Retrieve&db=Nucleotide&list_uids=154537610&dopt=GenBank&RID=UUU81PNH013&log$=nucltop&blast_rank=1) [gb\|EV709359.1\|](http://www.ncbi.nlm.nih.gov/entrez/query.fcgi?cmd=Retrieve&db=Nucleotide&list_uids=154487872&dopt=GenBank&RID=UUU81PNH013&log$=nucltop&blast_rank=2) [gb\|DV786200.1\|](http://www.ncbi.nlm.nih.gov/entrez/query.fcgi?cmd=Retrieve&db=Nucleotide&list_uids=82639076&dopt=GenBank&RID=UUU81PNH013&log$=nucltop&blast_rank=3) [gb\|DV783204.1\|](http://www.ncbi.nlm.nih.gov/entrez/query.fcgi?cmd=Retrieve&db=Nucleotide&list_uids=82636080&dopt=GenBank&RID=UUU81PNH013&log$=nucltop&blast_rank=4) [gb\|DT845120.1\|](http://www.ncbi.nlm.nih.gov/entrez/query.fcgi?cmd=Retrieve&db=Nucleotide&list_uids=75794411&dopt=GenBank&RID=UUU81PNH013&log$=nucltop&blast_rank=5) [gb\|DN511954.1\|](http://www.ncbi.nlm.nih.gov/entrez/query.fcgi?cmd=Retrieve&db=Nucleotide&list_uids=60722144&dopt=GenBank&RID=UUU81PNH013&log$=nucltop&blast_rank=6) [gb\|DT833930.1\|](http://www.ncbi.nlm.nih.gov/entrez/query.fcgi?cmd=Retrieve&db=Nucleotide&list_uids=75783221&dopt=GenBank&RID=UUU81PNH013&log$=nucltop&blast_rank=7) [gb\|CN442324.1\|](http://www.ncbi.nlm.nih.gov/entrez/query.fcgi?cmd=Retrieve&db=Nucleotide&list_uids=46421588&dopt=GenBank&RID=UUU81PNH013&log$=nucltop&blast_rank=8) [gb\|CN438686.1\|](http://www.ncbi.nlm.nih.gov/entrez/query.fcgi?cmd=Retrieve&db=Nucleotide&list_uids=46417950&dopt=GenBank&RID=UUU81PNH013&log$=nucltop&blast_rank=9) [gb\|EV751926.1\|](http://www.ncbi.nlm.nih.gov/entrez/query.fcgi?cmd=Retrieve&db=Nucleotide&list_uids=154547832&dopt=GenBank&RID=UUU81PNH013&log$=nucltop&blast_rank=10) [gb\|DN532563.1\|](http://www.ncbi.nlm.nih.gov/entrez/query.fcgi?cmd=Retrieve&db=Nucleotide&list_uids=60980078&dopt=GenBank&RID=UUU81PNH013&log$=nucltop&blast_rank=11) [gb\|CK953736.1\|](http://www.ncbi.nlm.nih.gov/entrez/query.fcgi?cmd=Retrieve&db=Nucleotide&list_uids=45468116&dopt=GenBank&RID=UUU81PNH013&log$=nucltop&blast_rank=12) [gb\|DN512629.1\|](http://www.ncbi.nlm.nih.gov/entrez/query.fcgi?cmd=Retrieve&db=Nucleotide&list_uids=60722819&dopt=GenBank&RID=UUU81PNH013&log$=nucltop&blast_rank=13) [gb\|DV790762.1\|](http://www.ncbi.nlm.nih.gov/entrez/query.fcgi?cmd=Retrieve&db=Nucleotide&list_uids=82643657&dopt=GenBank&RID=UUU81PNH013&log$=nucltop&blast_rank=14) [gb\|CF767959.1\|](http://www.ncbi.nlm.nih.gov/entrez/query.fcgi?cmd=Retrieve&db=Nucleotide&list_uids=37717178&dopt=GenBank&RID=UUU81PNH013&log$=nucltop&blast_rank=15)  [gb\|DN822570.1\|](http://www.ncbi.nlm.nih.gov/entrez/query.fcgi?cmd=Retrieve&db=Nucleotide&list_uids=62522158&dopt=GenBank&RID=UUU81PNH013&log$=nucltop&blast_rank=20)  [emb\|CR848939.2\|](http://www.ncbi.nlm.nih.gov/entrez/query.fcgi?cmd=Retrieve&db=Nucleotide&list_uids=61695543&dopt=GenBank&RID=UUU81PNH013&log$=nucltop&blast_rank=26) [gb\|CX951985.1\|](http://www.ncbi.nlm.nih.gov/entrez/query.fcgi?cmd=Retrieve&db=Nucleotide&list_uids=58765512&dopt=GenBank&RID=UUU81PNH013&log$=nucltop&blast_rank=27)  [gb\|DN642154.1\|](http://www.ncbi.nlm.nih.gov/entrez/query.fcgi?cmd=Retrieve&db=Nucleotide&list_uids=61947555&dopt=GenBank&RID=UUU81PNH013&log$=nucltop&blast_rank=53) |
| **Ly6g5b** | | | |
| ***Sequence name*** | ***Described before*** | ***Access number*** | |
|  |  | ***Sequence (nr/nt)*** | ***EST*** |
| BtLy6g5b-178 | NO |  |  |
| BtLy6g5b-284 | NO |  |  |
| BtLy6g5b-766 | NO |  |  |
| **Chimera Csnk2b-Ly6g5b** | | | |
| ***Sequence name*** | ***Described before*** | ***Access number*** | |
|  |  | ***Sequence (nr/nt)*** | ***EST*** |
| BtCsnk2b-Ly6g5b-201 | NO |  |  |
| BtCsnk2b-Ly6g5b-306 | NO |  |  |
| BtCsnk2b-Ly6g5b-368 | NO |  |  |
| BtCsnk2b-Ly6g5b-460 | NO |  |  |
| BtCsnk2b-Ly6g5b-640 | NO |  |  |
| BtCsnk2b-Ly6g5b-725 | NO |  |  |
| BtCsnk2b-Ly6g5b-737 | NO |  |  |
| BtCsnk2b-Ly6g5b-805 | NO |  |  |
| BtCsnk2b-Ly6g5b-1049 | NO |  |  |
| BtCsnk2b-Ly6g5b-1239 | NO |  |  |

| ***Rattus norvegicus*** | | | |
| --- | --- | --- | --- |
| **Csnk2b** | | | |
| ***Sequence name*** | ***Described before*** | ***Access number*** | |
|  |  | ***Sequence (nr/nt)*** | ***EST*** |
| RnCsnk2b-280 | NO |  |  |
| RnCsnk2b-662 | YES | [NM_031021.2](http://www.ncbi.nlm.nih.gov/entrez/query.fcgi?cmd=Retrieve&db=Nucleotide&list_uids=78214348&dopt=GenBank&RID=UWW9W0V0012&log$=nucltop&blast_rank=1)  [L15619.1](http://www.ncbi.nlm.nih.gov/entrez/query.fcgi?cmd=Retrieve&db=Nucleotide&list_uids=415717&dopt=GenBank&RID=UWW9W0V0012&log$=nucltop&blast_rank=2)  [BC078807.1](http://www.ncbi.nlm.nih.gov/entrez/query.fcgi?cmd=Retrieve&db=Nucleotide&list_uids=51261190&dopt=GenBank&RID=UWW9W0V0012&log$=nucltop&blast_rank=3) | [gb\|EV778558.1\|](http://www.ncbi.nlm.nih.gov/entrez/query.fcgi?cmd=Retrieve&db=Nucleotide&list_uids=154650325&dopt=GenBank&RID=UWW9TCER012&log$=nucltop&blast_rank=1)  [gb\|CK595937.1\|](http://www.ncbi.nlm.nih.gov/entrez/query.fcgi?cmd=Retrieve&db=Nucleotide&list_uids=41109005&dopt=GenBank&RID=UWW9TCER012&log$=nucltop&blast_rank=2) |
| **Ly6g5b** | | | |
| ***Sequence name*** | ***Described before*** | ***Access number*** | |
|  |  | ***Sequence (nr/nt)*** | ***EST*** |
| RnLy6g5b-283 | NO |  |  |
| RnLy6g5b-529 | NO |  |  |
| RnLy6g5b-658 | YES | [NM_001001934.1](http://www.ncbi.nlm.nih.gov/entrez/query.fcgi?cmd=Retrieve&db=Nucleotide&list_uids=50845390&dopt=GenBank&RID=UWXDNHYA013&log$=nucltop&blast_rank=1) |  |
| RnLy6g5b-754 | NO |  |  |
| **Chimera Csnk2b-Ly6g5b** | | | |
| ***Sequence name*** | ***Described before*** | ***Access number*** | |
|  |  | ***Sequence (nr/nt)*** | ***EST*** |
| RnCsnk2b-Ly6g5b-901 | NO |  |  |
| RnCsnk2b-Ly6g5b-2050 | NO |  |  |
| RnCsnk2b-Ly6g5b-2275 | NO |  |  |
| RnCsnk2b-Ly6g5b-2531 | NO |  |  |

| ***Mus musculus*** | | | |
| --- | --- | --- | --- |
| **Csnk2b** | | | |
| ***Sequence name*** | ***Described before*** | ***Access number*** | |
|  |  | ***Sequence (nr/nt)*** | ***EST*** |
| MumCsnk2b-132 | NO |  |  |
| MumCsnk2b-670 | YES | [NM_009975.2](http://www.ncbi.nlm.nih.gov/entrez/query.fcgi?cmd=Retrieve&db=Nucleotide&list_uids=118129820&dopt=GenBank&RID=UX6TRS6501R&log$=nucltop&blast_rank=1)  [BC003775.1](http://www.ncbi.nlm.nih.gov/entrez/query.fcgi?cmd=Retrieve&db=Nucleotide&list_uids=13277764&dopt=GenBank&RID=UX6TRS6501R&log$=nucltop&blast_rank=2) | [gb\|DV660992.1\|](http://www.ncbi.nlm.nih.gov/entrez/query.fcgi?cmd=Retrieve&db=Nucleotide&list_uids=89022163&dopt=GenBank&RID=UX6UHGK101R&log$=nucltop&blast_rank=1)  [gb\|DV047426.1\|](http://www.ncbi.nlm.nih.gov/entrez/query.fcgi?cmd=Retrieve&db=Nucleotide&list_uids=76374709&dopt=GenBank&RID=UX6UHGK101R&log$=nucltop&blast_rank=2)  [gb\|CB203415.1\|](http://www.ncbi.nlm.nih.gov/entrez/query.fcgi?cmd=Retrieve&db=Nucleotide&list_uids=28239773&dopt=GenBank&RID=UX6UHGK101R&log$=nucltop&blast_rank=3)  [gb\|BU513633.1\|](http://www.ncbi.nlm.nih.gov/entrez/query.fcgi?cmd=Retrieve&db=Nucleotide&list_uids=22821159&dopt=GenBank&RID=UX6UHGK101R&log$=nucltop&blast_rank=4)  [gb\|CX209909.1\|](http://www.ncbi.nlm.nih.gov/entrez/query.fcgi?cmd=Retrieve&db=Nucleotide&list_uids=56865201&dopt=GenBank&RID=UX6UHGK101R&log$=nucltop&blast_rank=5)  [gb\|CF584257.1\|](http://www.ncbi.nlm.nih.gov/entrez/query.fcgi?cmd=Retrieve&db=Nucleotide&list_uids=35197519&dopt=GenBank&RID=UX6UHGK101R&log$=nucltop&blast_rank=6)  [gb\|BI903481.1\|](http://www.ncbi.nlm.nih.gov/entrez/query.fcgi?cmd=Retrieve&db=Nucleotide&list_uids=16165409&dopt=GenBank&RID=UX6UHGK101R&log$=nucltop&blast_rank=7)  [gb\|BF608305.1\|](http://www.ncbi.nlm.nih.gov/entrez/query.fcgi?cmd=Retrieve&db=Nucleotide&list_uids=13504797&dopt=GenBank&RID=UX6UHGK101R&log$=nucltop&blast_rank=8)  [gb\|CB192884.1\|](http://www.ncbi.nlm.nih.gov/entrez/query.fcgi?cmd=Retrieve&db=Nucleotide&list_uids=28215043&dopt=GenBank&RID=UX6UHGK101R&log$=nucltop&blast_rank=9)  [gb\|BG968506.1\|](http://www.ncbi.nlm.nih.gov/entrez/query.fcgi?cmd=Retrieve&db=Nucleotide&list_uids=14356155&dopt=GenBank&RID=UX6UHGK101R&log$=nucltop&blast_rank=10)  [gb\|BI407153.1\|](http://www.ncbi.nlm.nih.gov/entrez/query.fcgi?cmd=Retrieve&db=Nucleotide&list_uids=15168076&dopt=GenBank&RID=UX6UHGK101R&log$=nucltop&blast_rank=11)  [gb\|CA465541.1\|](http://www.ncbi.nlm.nih.gov/entrez/query.fcgi?cmd=Retrieve&db=Nucleotide&list_uids=24921893&dopt=GenBank&RID=UX6UHGK101R&log$=nucltop&blast_rank=12)  [gb\|BI409048.1\|](http://www.ncbi.nlm.nih.gov/entrez/query.fcgi?cmd=Retrieve&db=Nucleotide&list_uids=15169971&dopt=GenBank&RID=UX6UHGK101R&log$=nucltop&blast_rank=13)  [dbj\|BY703400.1\|](http://www.ncbi.nlm.nih.gov/entrez/query.fcgi?cmd=Retrieve&db=Nucleotide&list_uids=27114507&dopt=GenBank&RID=UX6UHGK101R&log$=nucltop&blast_rank=14)  [gb\|BI407317.1\|](http://www.ncbi.nlm.nih.gov/entrez/query.fcgi?cmd=Retrieve&db=Nucleotide&list_uids=15168240&dopt=GenBank&RID=UX6UHGK101R&log$=nucltop&blast_rank=15)  [gb\|BI414961.1\|](http://www.ncbi.nlm.nih.gov/entrez/query.fcgi?cmd=Retrieve&db=Nucleotide&list_uids=15175884&dopt=GenBank&RID=UX6UHGK101R&log$=nucltop&blast_rank=16)  [gb\|BI412998.1\|](http://www.ncbi.nlm.nih.gov/entrez/query.fcgi?cmd=Retrieve&db=Nucleotide&list_uids=15173921&dopt=GenBank&RID=UX6UHGK101R&log$=nucltop&blast_rank=17)  [gb\|AI323164.1\|](http://www.ncbi.nlm.nih.gov/entrez/query.fcgi?cmd=Retrieve&db=Nucleotide&list_uids=4057593&dopt=GenBank&RID=UX6UHGK101R&log$=nucltop&blast_rank=18)  [gb\|BG967337.1\|](http://www.ncbi.nlm.nih.gov/entrez/query.fcgi?cmd=Retrieve&db=Nucleotide&list_uids=14354974&dopt=GenBank&RID=UX6UHGK101R&log$=nucltop&blast_rank=19)  [gb\|BI414146.1\|](http://www.ncbi.nlm.nih.gov/entrez/query.fcgi?cmd=Retrieve&db=Nucleotide&list_uids=15175069&dopt=GenBank&RID=UX6UHGK101R&log$=nucltop&blast_rank=20)  [gb\|CK793254.1\|](http://www.ncbi.nlm.nih.gov/entrez/query.fcgi?cmd=Retrieve&db=Nucleotide&list_uids=42805250&dopt=GenBank&RID=UX6UHGK101R&log$=nucltop&blast_rank=21)  [gb\|BI105292.1\|](http://www.ncbi.nlm.nih.gov/entrez/query.fcgi?cmd=Retrieve&db=Nucleotide&list_uids=14556185&dopt=GenBank&RID=UX6UHGK101R&log$=nucltop&blast_rank=22)  [gb\|BF235875.1\|](http://www.ncbi.nlm.nih.gov/entrez/query.fcgi?cmd=Retrieve&db=Nucleotide&list_uids=11148908&dopt=GenBank&RID=UX6UHGK101R&log$=nucltop&blast_rank=23)  [gb\|DV040071.1\|](http://www.ncbi.nlm.nih.gov/entrez/query.fcgi?cmd=Retrieve&db=Nucleotide&list_uids=76367335&dopt=GenBank&RID=UX6UHGK101R&log$=nucltop&blast_rank=24)  [gb\|CB574950.1\|](http://www.ncbi.nlm.nih.gov/entrez/query.fcgi?cmd=Retrieve&db=Nucleotide&list_uids=29494480&dopt=GenBank&RID=UX6UHGK101R&log$=nucltop&blast_rank=25)  [gb\|CA466326.1\|](http://www.ncbi.nlm.nih.gov/entrez/query.fcgi?cmd=Retrieve&db=Nucleotide&list_uids=24922678&dopt=GenBank&RID=UX6UHGK101R&log$=nucltop&blast_rank=26)  [gb\|CK020993.1\|](http://www.ncbi.nlm.nih.gov/entrez/query.fcgi?cmd=Retrieve&db=Nucleotide&list_uids=38546917&dopt=GenBank&RID=UX6UHGK101R&log$=nucltop&blast_rank=27)  [gb\|BQ961163.1\|](http://www.ncbi.nlm.nih.gov/entrez/query.fcgi?cmd=Retrieve&db=Nucleotide&list_uids=22376641&dopt=GenBank&RID=UX6UHGK101R&log$=nucltop&blast_rank=28)  [gb\|BG873607.1\|](http://www.ncbi.nlm.nih.gov/entrez/query.fcgi?cmd=Retrieve&db=Nucleotide&list_uids=14224147&dopt=GenBank&RID=UX6UHGK101R&log$=nucltop&blast_rank=29)  [gb\|CB574669.1\|](http://www.ncbi.nlm.nih.gov/entrez/query.fcgi?cmd=Retrieve&db=Nucleotide&list_uids=29494199&dopt=GenBank&RID=UX6UHGK101R&log$=nucltop&blast_rank=32)  [gb\|BF533641.1\|](http://www.ncbi.nlm.nih.gov/entrez/query.fcgi?cmd=Retrieve&db=Nucleotide&list_uids=11621004&dopt=GenBank&RID=UX6UHGK101R&log$=nucltop&blast_rank=34)  [gb\|CF618691.1\|](http://www.ncbi.nlm.nih.gov/entrez/query.fcgi?cmd=Retrieve&db=Nucleotide&list_uids=37236823&dopt=GenBank&RID=UX6UHGK101R&log$=nucltop&blast_rank=35)  [gb\|AI323548.1\|](http://www.ncbi.nlm.nih.gov/entrez/query.fcgi?cmd=Retrieve&db=Nucleotide&list_uids=4057977&dopt=GenBank&RID=UX6UHGK101R&log$=nucltop&blast_rank=43)  [gb\|CO800116.1\|](http://www.ncbi.nlm.nih.gov/entrez/query.fcgi?cmd=Retrieve&db=Nucleotide&list_uids=50988296&dopt=GenBank&RID=UX6UHGK101R&log$=nucltop&blast_rank=44) |
| MumCsnk2b-780 | NO |  |  |
| **Ly6g5b** | | | |
| ***Sequence name*** | ***Described before*** | ***Access number*** | |
|  |  | ***Sequence (nr/nt)*** | ***EST*** |
| MumLy6g5b-611 | YES | [BC131964.1](http://www.ncbi.nlm.nih.gov/entrez/query.fcgi?cmd=Retrieve&db=Nucleotide&list_uids=124297572&dopt=GenBank&RID=UX829DT6016&log$=nucltop&blast_rank=2)  [BC131966.1](http://www.ncbi.nlm.nih.gov/entrez/query.fcgi?cmd=Retrieve&db=Nucleotide&list_uids=124298057&dopt=GenBank&RID=UX829DT6016&log$=nucltop&blast_rank=1)  [AJ315553.1](http://www.ncbi.nlm.nih.gov/entrez/query.fcgi?cmd=Retrieve&db=Nucleotide&list_uids=21622574&dopt=GenBank&RID=UX829DT6016&log$=nucltop&blast_rank=4)  [NM_148939.2](http://www.ncbi.nlm.nih.gov/entrez/query.fcgi?cmd=Retrieve&db=Nucleotide&list_uids=50845392&dopt=GenBank&RID=UX829DT6016&log$=nucltop&blast_rank=3) |  |
| MumLy6g5b-707 | YES | [BC145150.1](http://www.ncbi.nlm.nih.gov/entrez/query.fcgi?cmd=Retrieve&db=Nucleotide&list_uids=219519812&dopt=GenBank&RID=UX8EKCGD012&log$=nucltop&blast_rank=1)  [AJ315554.1](http://www.ncbi.nlm.nih.gov/entrez/query.fcgi?cmd=Retrieve&db=Nucleotide&list_uids=21622576&dopt=GenBank&RID=UX8EKCGD012&log$=nucltop&blast_rank=2) |  |
| **Chimera Csnk2b-Ly6g5b** | | | |
| ***Sequence name*** | ***Described before*** | ***Access number*** | |
|  |  | ***Sequence (nr/nt)*** | ***EST*** |
| MumCsnk2b-Ly6g5b-713 | NO |  |  |
| MumCsnk2b-Ly6g5b-979 | NO |  |  |
| MumCsnk2b-Ly6g5b-1108 | NO |  |  |
